# Transcriptomic variation of pharmacogenes in multiple human tissues and lymphoblastoid cell lines

**DOI:** 10.1038/tpj.2015.93

**Published:** 2016-02-09

**Authors:** A Chhibber, C E French, S W Yee, E R Gamazon, E Theusch, X Qin, A Webb, A C Papp, A Wang, C Q Simmons, A Konkashbaev, A S Chaudhry, K Mitchel, D Stryke, T E Ferrin, S T Weiss, D L Kroetz, W Sadee, D A Nickerson, R M Krauss, A L George, E G Schuetz, M W Medina, N J Cox, S E Scherer, K M Giacomini, S E Brenner

**Affiliations:** 1 Department of Bioengineering and Therapeutic Sciences, University of California, San Francisco, San Francisco, CA, USA; 2 Departments of Molecular and Cell Biology, University of California, Berkeley, CA, USA; 3 Division of Genetic Medicine, Department of Medicine, Vanderbilt University, Nashville, TN, USA; 4 Academic Medical Center, University of Amsterdam, Amsterdam, The Netherlands; 5 Children's Hospital Oakland Research Institute, Oakland, CA, USA; 6 Human Genome Sequencing Center, Baylor College of Medicine, Houston, TX, USA; 7 Department of Biomedical Informatics, College of Medicine, The Ohio State University Wexner Medical Center, Columbus, OH, USA; 8 Center for Pharmacogenomics; College of Medicine, The Ohio State University Wexner Medical Center, Columbus, OH, USA; 9 Department of Pharmaceutical Sciences, St. Jude Children's Research Hospital, Memphis, TN, USA; 10 Department of Pharmaceutical Chemistry, University of California, San Francisco, San Francisco, CA, USA; 11 Channing Division of Network Medicine, Department of Medicine, Brigham and Women's Hospital, Boston, MA, USA; 12 Departments of Pharmacology, Psychiatry, and Human Genetics/Internal Medicine, College of Medicine; Colleges of Pharmacy and Environmental Health Sciences, The Ohio State University, Columbus, OH, USA; 13 Department of Genome Sciences, University of Washington, Seattle, WA, USA; 14 Department of Pharmacology, Northwestern University Feinberg School of Medicine, Chicago, IL, USA; 15 Department of Plant and Microbial Biology, University of California, Berkeley, CA, USA

## Abstract

Variation in the expression level and activity of genes involved in drug disposition and action (‘pharmacogenes') can affect drug response and toxicity, especially when in tissues of pharmacological importance. Previous studies have relied primarily on microarrays to understand gene expression differences, or have focused on a single tissue or small number of samples. The goal of this study was to use RNA-sequencing (RNA-seq) to determine the expression levels and alternative splicing of 389 Pharmacogenomics Research Network pharmacogenes across four tissues (liver, kidney, heart and adipose) and lymphoblastoid cell lines, which are used widely in pharmacogenomics studies. Analysis of RNA-seq data from 139 different individuals across the 5 tissues (20–45 individuals per tissue type) revealed substantial variation in both expression levels and splicing across samples and tissue types. Comparison with GTEx data yielded a consistent picture. This in-depth exploration also revealed 183 splicing events in pharmacogenes that were previously not annotated. Overall, this study serves as a rich resource for the research community to inform biomarker and drug discovery and use.

## Introduction

Variation in the expression levels and splicing of drug metabolizing enzymes, transporters and targets, such as receptors and ion channels, has been associated with inter-individual differences in optimal drug dose, drug efficacy and adverse drug events.^1, 2^ Thus, a comprehensive study of variation in the transcriptome profiles of pharmacologically relevant tissues promises to yield important insights into the molecular basis of variation in drug response. Technological advances in quantifying the transcriptome and the rapid development of high-throughput screening methodologies have led to the identification and characterization of many biomarkers of drug response.^3, 4^ These innovations have transformed the way we design and analyze pharmacogenomics studies and are increasingly informing development of approaches to clinical practice.

Transcriptome sequencing, or RNA-sequencing (RNA-seq), is facilitating analyses at the transcript level with an unprecedented resolution. As the technology has developed, longer reads and higher throughput have allowed for detailed evaluation of whole transcriptomes across many samples.^5^ Analytical approaches have emerged, including Cufflinks^6^ and DESeq^7^ for gene expression analysis and DEXSeq,^8^ MISO^9^ and JuncBASE^10^ for splicing analysis. However, the use of next-generation sequencing technology for pharmacogenomics research has been limited.^4, 11^ Although community-wide efforts such as the Genotype Tissue Expression Project^12^ are facilitating studies of expression quantitative trait loci, there has not been an application of RNA-seq to large sample sets across diverse human tissues with a focus on genes involved in drug disposition and tissues of greater pharmacological relevance and action.

In pharmacogenomics, polymorphisms that affect expression levels or result in alternative splicing of drug metabolizing enzymes are known to have large effects on drug disposition and response. For example, UGT1A1*28 (rs8175347), with seven thymine–adenine^13^ repeats in the promoter region, leads to reduced transcription rates of this enzyme and profound toxicity in patients receiving the topoisomerase inhibitor, irinotecan.^14, 15^ Likewise, alternative splicing of *CYP2D6* occurs frequently in human populations and is responsible for reduced activity of the enzyme.^16^ Given these large and clinically important effects in drug-metabolizing enzymes, a systematic study of the transcriptome with a focus on pharmacogenes is clearly needed. Although several research groups have performed transcriptome profiling and alternative splicing event analyses in human cell lines and tissues,^17, 18, 19^ these studies are limited to single tissue types or use pooled samples. Thus, information about inter-individual variation in gene expression and splicing from a given tissue type or inter-tissue variation is limited, despite the value of such studies in identifying biomarkers for differential drug response or toxicity.

Given these limitations, the National Institutes of Health-supported Pharmacogenomics Research Network (PGRN) initiated a transcriptome sequencing project to catalog variation in gene expression and splicing across individuals in tissues and genes of pharmacologic importance. Tissues studied include liver, a key organ for drug metabolism,^20, 21^ kidney, the site of excretion for many drugs,^22^ as well as heart and adipose tissue, where pharmacogenes can affect local drug distribution and action.^23^ Lymphoblastoid cell lines (LCLs) were also included, as they have been widely used as a cell-based model for a variety of pharmacogenomics studies.^24, 25, 26^ In this article, we characterized the variability in the expression and splicing of 389 PGRN pharmacogenes across individuals and between four human tissue types and LCLs, and identified novel alternative splicing events in these samples. Furthermore, we provide this information for community use, in the form of expression and splicing profiles for 139 individuals. This resource will be valuable for future pharmacogenomics studies as both a discovery and validation platform.

## Materials and methods

### Selection of pharmacogenes

Protein coding genes were defined as those with a start codon in the Gencode v12 (ref. 13) annotation. A subset of these was defined as ‘PGRN pharmacogenes'. Our list of 389 pharmacogenes was compiled from PharmGKB,^27^ a curated knowledge base about the impact of genetic variation on drug response, PharmaADME,^28^ the US Food and Drug Administration (FDA) Pharmacogenomics Biomarkers^29^ and the literature.^24, 30, 31, 32, 33^ Genes that are annotated in at least two of these resources or publications were selected as PGRN pharmacogenes. These include 160 enzymes, 84 transporters, 15 ion channels, 27 receptors, 24 nuclear receptors and other transcription factors, as well as 22 other genes, including G-protein coupled receptors that are drug targets and have an important role in drug disposition, response or toxicity (Supplementary Table S1).

### Tissue collection, RNA isolation and preparation of RNA-seq library

Tissue from 24 liver, 20 kidney (cortex), 25 heart (left ventricle), 25 adipose (subcutaneous) samples and 45 LCLs were obtained from PGRN research groups: the Pharmacogenomics of Anticancer Agents Research in Children provided liver tissues, Pharmacogenomics of Membrane Transporters provided kidney samples, Pharmacogenomics and Risk of Cardiovascular Disease provided adipose tissue and LCLs, and Pharmacogenomics of Arrhythmia Therapy provided heart tissue. Demographic information on the samples is described in Supplementary Table S2.

Total RNA was extracted for each sample, selected for mRNA by poly-A selection, and then fragmented to a mean length of ~120 to 180 base pairs. Strand-specific complementary DNA libraries were prepared and sequenced on an Illumina HiSeq 2000 (San Diego, CA, USA) at depths of 45–171 million paired-end 100 bp reads per sample.

### Alignment and transcriptome analysis

Raw reads were mapped to the human genome sequence (hg19)^34^ using Tophat v2.0.6^35^ and PCR duplicates were removed. Some samples had a low percentage of unique reads likely due to limited starting material. Transcript structure assembly was performed with Cufflinks (v.2.0.2)^6^ on each sample for each tissue type. To control for differing sequencing depths between tissue types, and the variable number of samples analyzed for each tissue type, gene expression analysis was performed on a subset of the data: 20 million reads per sample and 18 samples per tissue type. Gene expression values (in Fragments per Kilobase of Exon Mapped, FPKM) were calculated by summing per-isoform FPKM values generated by Cuffdiff (v2.2.1)^6^ for each sample or by tissue type. Throughout, gene estimates are used unless isoforms are specifically mentioned.

To discover novel splice events and analyze differential splicing, the subsampled reads were run through the JuncBASE v0.6^10^ pipeline. JuncBASE uses junction reads from an RNA-seq experiment to calculate inclusion and exclusion of individual splicing events. These are measured as percent spliced in (PSI). Such measures are generally more reliable than isoform reconstruction as they require less inference.

### Validation

To validate selected splice events that were not found in the gene annotations, we created primers specific to the novel event and looked for amplification by PCR using pooled liver complementary DNA (Supplementary Figure S1). To validate the PSI estimates derived from RNA-seq, PSI values for two common and previously annotated splice variants in *HMGCR13(−)*^13^ and *LDLR4(−)*,^13^ were quantified by quantitative PCR in LCLs (*n*=39) from the same RNA that was used to prepare the RNA-seq libraries. The PSI values for these two events in LCLs calculated by quantitative PCR and RNA-seq were positively correlated with *R*^2^-values of 0.43 and 0.5, respectively (Supplementary Figure S2).

To validate the patterns of pharmacogene expression and splicing identified in this study, we analyzed data from the Genotype Tissue Expression Project (v4).^36^ Expression (RPKM, mapped reads per kilobase per million mapped reads) values per individual per gene were downloaded from the Genotype Tissue Expression Project portal (http://gtexportal.org) to study the variability in gene expression and patterns of expression across tissues. Aligned reads were downloaded from SRA/dbGaP and run through the JuncBASE pipeline in the same way as was done for the PGRN data to compare differential splicing patterns between the two data sets and novel junctions identified in the PGRN data set.

Further details regarding all methodology are included in the Supplementary methods.

## Results

### The PGRN RNA-seq project

**Figure 1:**
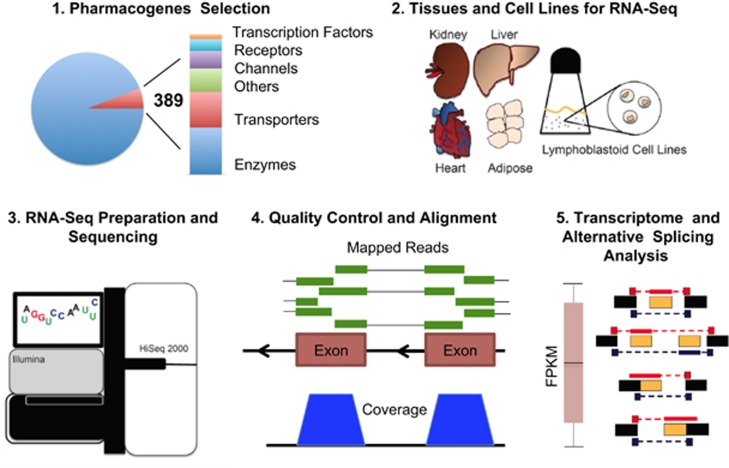
Overview of the Pharmacogenomics Research Network (PGRN) RNA-seq project. (1) 389 ‘PGRN pharmacogenes' were selected representing genes that have a key role in drug disposition. (2) RNA from multiple samples for human liver, heart, kidney, adipose tissue and lymphoblastoid cell lines was collected. (3) Complementary DNA libraries were prepared from these samples and sequenced using an Illumina HiSeq 2000. (4) Rigorous pre- and post- alignment quality control procedures were applied to the data. (5) Gene expression was quantified and splicing events identified for the PGRN pharmacogenes across samples and tissue types. This information is provided as a resource to the pharmacogenomics community.

The PGRN RNA-seq project was designed to provide in-depth investigation of the transcriptomes of pharmacologically relevant human tissues with a focus on genes of particular interest to the pharmacogenomics community (Figure 1). In order to study inter-individual variability in expression and splicing of PGRN pharmacogenes (Supplementary Table S1), we generated transcriptome sequencing data from 24 liver, 20 kidney, 25 heart and 25 adipose samples and 45 LCLs (Supplementary Table S2). For each sample, reads were mapped to the human genome,^35^ resulting in 10–97 million mapped reads per sample. To control for this substantial difference in sequencing depth and sample number between tissues, 18 samples for each tissue were selected and subsampled down to 20 million reads/sample for further expression and splicing analyses, resulting in a total of 90 samples. Gene expression and splicing results are available for download for all samples (http://pharmacogenetics.ucsf.edu/expression/rnaseqdata.html). The expression profiles of all pharmacogenes across tissues and individuals is included in Supplementary Figure S3. A brief overview of alternative splicing and gene expression of all protein-coding genes can be found in the Supplementary Results.

### Analysis of PGRN pharmacogene gene expression

**Figure 2:**
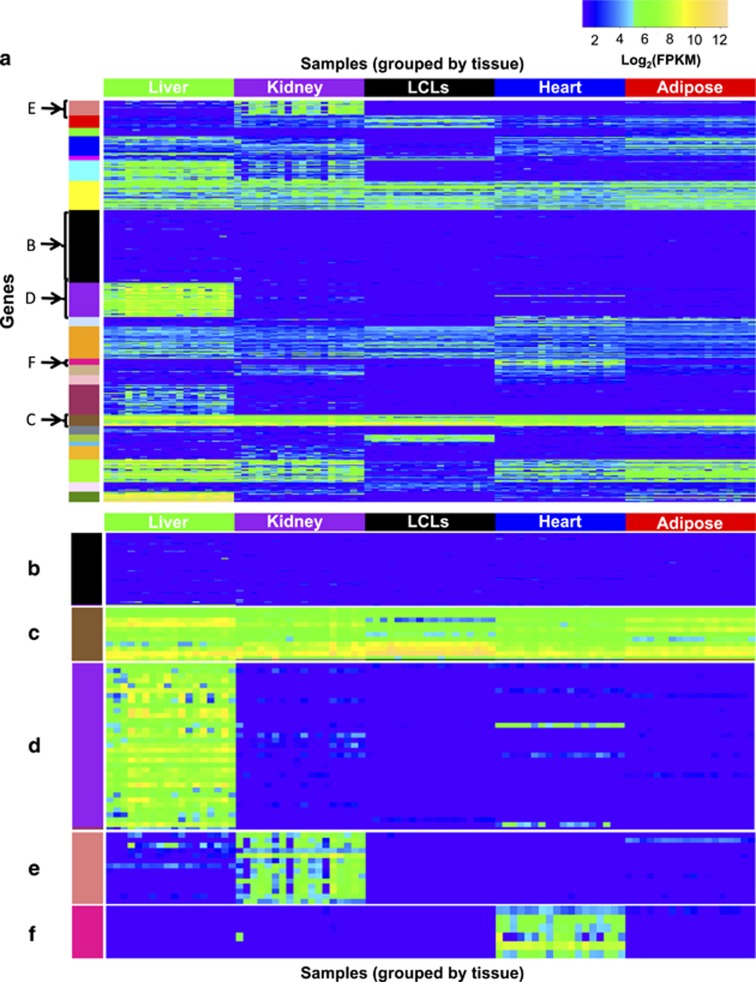
(**a**) Heatmap of the 389 Pharmacogenomics Research Network pharmacogenes' expression (Fragments per Kilobase of Exon Mapped, FPKM) across 90 samples. Samples are arranged horizontally, grouped by tissue. Pharmacogenes are arranged vertically, grouped by clusters identified by k-means clustering; clusters are indicated by colors along the left side of the heatmap. Selected clusters show (**b**) genes expressed at low levels across all samples (*ABCB5, ABCC12, ABCC8, ADH7, ADRB3, ALDH3A1, BDNF, CACNA1S, CFTR, CHRM3, CHST13, CHST4, CHST5, CHST6, CHST8, CRHR1, CYP11B1, CYP11B2, CYP26A1, CYP26C1, CYP2A13, CYP2F1, CYP2S1, CYP4F8, CYP4Z1, CYP7A1, DRD1, DRD2, DRD3, DRD4, DRD5, ESR2, FMO6P, GNB3, GRM3, GSTA3, GSTA5, GSTT2, HTR1A, HTR2A, IL28B, KCNE2, MMP3, OPRM1, P2RY1, PNMT, PRSS53, RYR1, SCN3B, SLC10A2, SLC22A13, SLC22A14, SLC22A16, SLC22A4, SLC28A2, SLC28A3, SLC6A3, SLC6A4, SLCO1A2, SLCO6A1, SULT1A3, SULT4A1, TPH1, TPH2, TPSG1, UGT1A10, UGT1A5, UGT1A8, UGT2B11* and *UGT2B28)* (**c**) genes highly expressed across all samples (*ADD1, ADH5, ALDH2, CYB5A, CYB5R3, GSTK1, GSTO1, GSTP1, HLA-B, RPL13* and *SOD2*) or genes expressed at higher levels in (**d**) liver (*ABCB4, ABCC2, ADH1A, ADH4, APOA4, APOB, CYP2A6, CYP2B6, CYP2C18, CYP2C8, CYP2C9, CYP2D6, CYP2J2, CYP3A4, CYP3A5, CYP4F11, CYP8B1, F2, F5, MAT1A, NAT2, PON1, PON3, SERPINA7, SLC22A1, SLCO1B1, SLCO1B3, SULT2A1, UGT1A1, UGT1A4, UGT2B10, UGT2B15* and *UGT2B4)*, (**e**) kidney (*ABP1, FMO1, GSTA2, GSTO2, HSD11B2, SLC13A1, SLC13A3, SLC22A11, SLC22A12, SLC22A2, SLC22A6, SLC22A8, SULT1C2* and *UGT8*), or (**f**) heart (*ADRB1, CACNA1C, KCNH2, NPPB, RYR2* and *SCN5A*). Gene names are listed in order from top to bottom in each cluster in the figure. Plot drawn using R package gplots. LCL, lymphoblastoid cell line.^74^

We found that 161 (of 389) of our PGRN pharmacogenes were expressed at FPKM ⩾1 in at least one sample across all 5 tissue types in our data set and 87 pharmacogenes were expressed at FPKM ⩾1 in all samples of all 5 tissue types (Supplementary Table S3A). As a group, PGRN pharmacogenes were significantly enriched for variable gene expression between individuals, and were among the top 10 most variably expressed gene sets (classified by gene ontology biological process^37^) in the physiological tissues (Supplementary Table S4). We also observed subsets of pharmacogenes that showed similar patterns of expression across the different tissues (k-means clustering of gene expression of 389 pharmacogenes, Figure 2a). For example, some pharmacogenes were expressed consistently at low levels across all tissues and samples (for example, ABCC12 and ESR2, Figure 2b). In contrast, 11 pharmacogenes were very highly expressed, although to different levels, across all tissues and LCLs (Figure 2c); these include genes involved in mitochondrial structure or function (*ADH5*, *ALDH2*, *CYB5R3* and *SOD2*) and glutathione transferase activity (*GSTK1*, *GSTO1* and *GSTP1*).^37^

**Figure 3:**
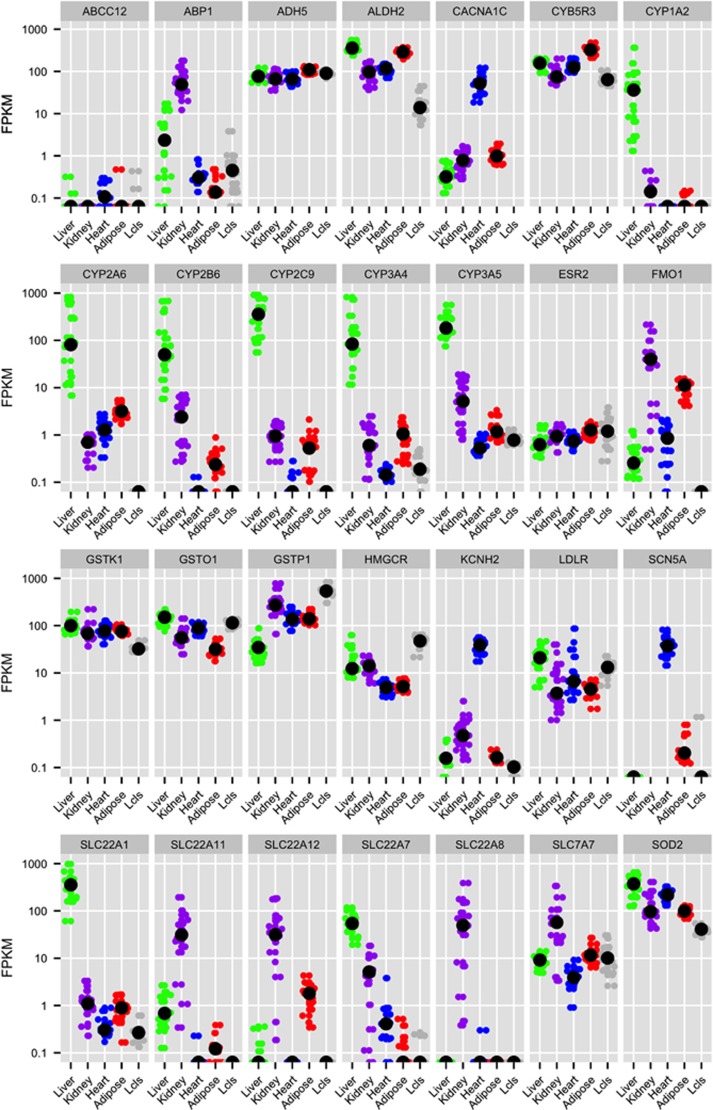
Gene expression (Fragments per Kilobase of Exon Mapped, FPKM) by sample across each tissue type and lymphoblastoid cell lines (LCLs) for selected cytochrome P450 (CYP) enzymes, solute carrier family (SLC) transporters, and other pharmacogenes discussed in this article from subsampled data (18 samples per tissue type, 20 million reads per sample). The black dot indicates median FPKM per gene and tissue type. See Supplementary Figure 3 for plots for all pharmacogenes. Plots drawn using R package ggplot2.^75^

Not surprisingly, PGRN pharmacogenes are generally more highly expressed in liver compared with the other tissues (Supplementary Figure S4). Many genes coding for xenobiotic metabolizing enzymes and transporters were highly and specifically expressed in the liver, an organ important for drug metabolism (Figure 2d, Supplementary Table S5A). Pharmacogenes expressed at highest abundance in the kidney, the major organ for secretion and reabsorption, include a number of solute carrier transporters (*SLC* genes; Figure 2e), which have important roles in drug secretion or reabsorption,^38^ as well as enzymes such as *ABP1* and *FMO1*. In addition, pharmacogene expression levels in the liver and kidney varied greatly among individuals. For example, the expression levels of a number of CYPs in the liver and SLC transporters in the kidney varied by over 100- and 1000-fold, respectively (Figure 3).

The list of PGRN pharmacogenes included 119 (out of 389) genes that are currently drug targets or are under clinical development as potential targets for various diseases.^39^ These drug target genes may be expressed abundantly in tissues not primarily involved in drug disposition. For example, a small number of pharmacogenes were highly expressed solely in the heart (Figure 2f). These genes are all involved with cardiac contractility and include, for example, ion channels involved in cardiac conductance (*SCN5A*, *CACNA1C* and *KCNH2*) that are targeted by many drugs.^40, 41, 42^ Most pharmacogenes expressed (FPKM ⩾1) in adipose tissue were expressed in other tissues as well (Figure 2a). The strongest correlation of pharmacogene expression profiles among tissues were detected between adipose and heart (*r*=0.83), as is true for all protein-coding genes expression between adipose and heart (*r*=0.90; Supplementary Table S6).

Compared with the four physiological tissues, LCLs showed lower overall expression levels of pharmacogenes: proportionally fewer pharmacogenes were expressed in at least one LCL sample or expressed in all LCL samples compared with all protein-coding genes (*χ*^2^-test: 48 vs 64%, *P*<0.0001 and 30 vs 48%, *P*<0.0001 in at least one sample or all samples, respectively, Supplementary Table 3). Pharmacogenes expressed at lower levels in LCLs than in the tissues assayed include genes important for drug disposition—for example, genes coding for enzymes (cytochrome P450s, UGTs and SULTs), SLC transporters, ion channels and receptors (Supplementary Table S5B).

### Analysis of PGRN pharmacogene splicing

We found that 278 of the 389 pharmacogenes (72%) showed clear evidence of being alternatively spliced (⩾2 isoforms) in our data set. Receptor and channel genes are the least alternatively spliced (<50%, Supplementary Table S7), although, likely due to the small numbers of genes, only receptors are significantly depleted (Bonferroni-corrected *P*<0.05, hypergeometric test). Another 66 pharmacogenes had inconclusive evidence of being alternatively spliced either because the alternative splice event is very rare, or because of low gene expression. The other 45 pharmacogenes are substantially expressed (FPKM>10) in at least one sample but have no evidence of alternative splice events in this data set.

**Figure 4:**
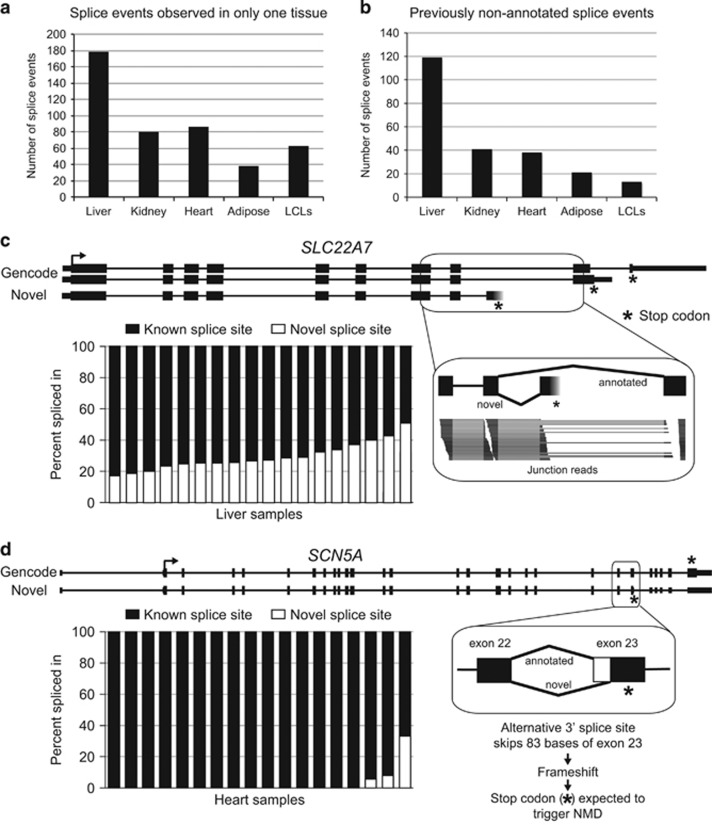
(**a**) Splice events in Pharmacogenomics Research Network pharmacogenes with PSI (percent spliced in) ⩾5 and coverage ⩾1 reads/100 bp in at least one sample of one tissue and no coverage in any of the four other tissues. (**b**) Splice events in pharmacogenes not present in current gene annotations with coverage ⩾5 reads/100 bp in at least one sample. These splice events were identified in 68, 31, 18, 16, and 10 pharmacogenes in liver, kidney, heart, adipose tissue and lymphoblastoid cell lines (LCLs), respectively. (**c**) An alternative last exon in *SLC22A7*, not previously annotated, was observed in liver samples and would alter the C-terminal end of the protein. Chart: fraction of transcripts from *SLC22A7* that contain the novel (white) or known (black) splice event in each liver sample. Inset: reads crossing the alternative junctions in a liver sample. (**d**) A novel alternative 3' splice site in *SCN5A* was identified that results in an 83-base deletion of the coding sequence of *SCN5A*, creating a premature stop codon expected to trigger nonsense-mediated mRNA decay. Chart: fraction of transcripts from *SCN5A* that contain the novel (white) or known (black) splice event in each heart sample.

Differential alternative splicing between pairs of tissues was evident for dozens of PGRN pharmacogenes (Wilcoxon test, False Discovery Rate<0.05; difference in median PSI >5 Supplementary Table S8), with LCLs showing the greatest differences in splicing events compared with the other tissues. We also found dozens of inferred splice events that were only observed in one of our five tissue types, often because the gene was not expressed in other tissues but also possibly because only alternative splice events were used in those tissue types (Figure 4a). When we control for gene expression differences between tissues by requiring the potentially alternatively spliced region to have high total read coverage in a number of samples for the four other tissues, we see only a very small fraction of genes (0–5%) have tissue-specific splice events (Supplementary Table S9).

Notably, a total of 183 alternative splicing events (in 102 out of 389 genes) included splice junctions not previously annotated, but which were present with a robust coverage of at least 5 reads/100 bp in at least one sample (Figure 4b). The greatest number of previously non-annotated pharmacogene splicing events was observed in the liver samples, likely because many of those genes are very highly expressed in that tissue, making it easier to observe these often low expressed events. One of the novel splicing events observed in liver was an alternative last exon of *SLC22A7*, a gene that encodes a transporter of endogenous compounds and prescription drugs (Figure 4c). This newly found alternative event was validated by PCR (Supplementary Figure S1), is predicted to produce a protein with a truncated C terminus, and was substantially and variably expressed in the liver samples. A novel splicing event observed in heart was in *SCN5A*, a gene encoding a sodium channel important in maintaining normal cardiac rhythm (Figure 4d). Observed in three heart samples, this novel alternative 3′ splice site in exon 23 excludes 83 bases and generates a downstream premature termination codon that is expected to cause the transcript to be degraded by the nonsense-mediated mRNA decay pathway.

## Discussion

Over the last several years, there have been many studies using RNA-seq to quantify gene expression and to identify novel alternative splicing events in many tissue and cell types.^43, 44, 45, 46, 47, 48^ Here, we applied this approach to characterize the expression of 389 genes of pharmacologic importance (genes involved in drug disposition, response or toxicity) in multiple human tissue types and LCLs. Unlike many other transcriptome profiling studies using RNA-seq, this report presents findings for multiple samples across tissues, allowing the capture of inter-individual variation in expression levels in addition to comparison of expression and splicing across different tissues. Further, results in multiple subjects act as biological replicates for a given tissue type, allowing for a more accurate representation of tissue-specific splicing and expression. By incorporating inter-individual variation in our study of several human tissues, our data represent an important addition to our understanding of human transcriptomics. This data set is available at http://pharmacogenetics.ucsf.edu/expression/rnaseqdata.html (and at doi:10.6078/D1RG66).

In comparing global analyses of protein-coding and pharmacogene expression, we observed several interesting patterns. Prominently, the majority of PGRN pharmacogenes were expressed at lower levels in LCLs compared with the four physiological tissues studied, in contrast to expression levels across all protein-coding genes. As an actively and aggressively proliferating cell type, gene expression in LCLs is tuned to growth, and thus relative expression of genes involved in other cellular processes may be suppressed. Furthermore, it is possible that peripheral B-lymphocytes, the primary cells from which LCLs are derived, also show significantly different patterns of expression from the other four physiological tissues included in this study. These results suggest that consideration of the phenotype and gene of interest is important when using LCLs as a proxy for other tissues in pharmacogenetic studies, as well as when using tissues as proxies for each other. Overall, more pharmacogenes were expressed at higher levels in the liver compared with other tissues. Although this result is not unexpected given the importance of the liver in drug metabolism and transport and the bias toward liver-specific genes in the field of pharmacogenomics, it also demonstrates the importance of conducting studies in samples of the relevant tissue type where possible. We also observed high correlation in gene expression values between adipose and heart tissues among both protein-coding genes and the subset of PGRN pharmacogenes. This result is consistent with the finding that adipose derived stem cells have been shown to spontaneously differentiate into cardiomyocytes and that both adipose and cardiac tissues derive from the mesoderm.^49, 50^

We also observed interesting patterns of alternative splicing in this study, including the discovery of splicing events not previously annotated and significant differential splicing between LCLs and other tissue types. Since splicing detection is dependent on sequencing coverage and the number of samples analyzed, we investigated the effects of subsampling down the number of reads and samples to make them equivalent between tissues (Supplementary Results, Supplementary Figure S6). Using only 18 samples per tissue, we were still able to detect 95% of splice events we would be able to observe with all samples in our data set. Subsampling the reads limits the detection of rare splicing events and that particularly affects novel splice events, as they generally have low PSI values and, thus, low read coverage (Supplementary Figure S7), or occur in only a small number of samples. As rare splice events may represent physiologically relevant alternative splicing, the splicing results from using all of our data (all reads, all samples) are also available to download.

Among the splicing events identified, we observe both previously characterized as well as novel alternative splicing events. For example, an alternative 3′ splice site in the drug target *SCN5A* generates a premature termination codon predicted to trigger the nonsense-mediated mRNA decay pathway (NMD). *SCN5A* encodes the main cardiac voltage-gated sodium channel important in maintaining normal cardiac conduction. A number of drugs target sodium channels, including antiarrhythmics and non-antiarrhythmic sodium channel blockers. Changes in structure, activity and expression of drug targets, such as that encoded by SCN5A, can alter the efficacy of drugs designed to target these proteins.^51, 52^ This event may be indicative of a novel role for alternative splicing coupled with NMD in the regulation of this gene.^53^ In addition, a novel truncated isoform of the transporter *SLC22A7* was identified. The gene *SLC22A7* is expressed in both kidney and liver and is important for transport of endogenous compounds^54^ and a number of prescription drugs.^55, 56, 57^

We also observed substantial variability in gene expression, particularly among drug transporters and drug metabolizing enzymes. In the liver, several cytochrome P450 (CYP) enzymes showed significant variability in expression levels between individuals; such variability can drive differences in drug metabolism across individuals, leading to variation in drug efficacy and susceptibility to toxicity.^58^ One example includes CYP3A4, which is responsible for activation and deactivation of a number of drugs by oxidation in the liver. Induction of CYP3A4 by concomitant medications or dietary supplements is well-established, and is considered a major source of variation in drug response.^59^ The enormous inter-individual variation in the expression levels of CYP3A4 we observe in the liver samples may be due to differences in diet, including dietary supplements, or medications among the individuals, in addition to genetic variation. Like drug metabolism, renal elimination of drugs is also variable across individuals in part due to the variation in renal secretion and reabsorption; this variation can be driven by differences in expression levels of renal transporters across individuals. We observed profound differences in the expression levels of renal secretory and reabsorptive transporters, particularly the SLCs. For example, expression of the uric acid transporter *SLC22A12* varied almost 1000-fold between individuals in the kidney (Figure 3). As a target for drugs that treat hyperuricemia,^60^ the expression level of *SLC22A12* could be an important determinant of drug response.

As is true of any study using human organs, while only healthy tissues were used for mRNA extraction, the patients themselves may have had a disease affecting other organs or may have been taking medications. In particular for the study of pharmacogenes, the variability in xenobiotic exposure is a concern, as such exposure is known to alter pharmacogene expression^61, 62^ and splicing^63, 64, 65, 66, 67^ profiles. The fact that the variability in splicing and expression both within and between tissues was similar to that identified in an analogous analysis of an independently derived RNA-seq data set (from the GTEX project, see Supplementary Results) suggests that the patterns of splicing and expression detected are not driven by a single overrepresented disease, phenotype or environmental exposure in our data set. However, in both data sets the variability detected may be driven in part by differences in health status or exposures between individuals. Other potential sources of variability in our dataset include subtle differences in cellular composition of the tissue samples or sample collection protocols, as well as patient age and sex;^68, 69^ for example, a few pharmacogenes appeared to show higher expression levels in samples from pediatric patients. Given the small sample sizes and skewed sex and age distributions in some of the tissue types, this study was not optimal for investigating variation due to these two factors. Finally, despite substantial variability in expression in some pharmacogenes between individuals, other pharmacogenes showed very consistent expression between tissues and/or across individuals (e.g., ADH5 and GSTK1), suggesting that the extensive variability observed was not driven by noise in the experimental process.

Pharmacogenomic studies have largely focused on the effects of genetic polymorphisms in pharmacogenes on drug response and drug toxicity.^70, 71^ Our data suggest that genes involved in drug disposition and toxicity can be variably spliced and expressed among individuals and across tissues. Furthermore, given that splicing can affect expression, localization and function of genes,^72, 73^ our results suggest that splicing may be a relatively unexplored source of variability in drug response, toxicity and efficacy. Transcriptome profiling (including both expression and splicing) of pharmacogenes may be a valuable tool for identification of mechanisms and possible prediction of drug response variability. As the first in-depth analysis of transcript structure and expression of genes that have a key role in drug disposition, this PGRN RNA-seq resource will be valuable for biomarker and drug target discovery and validation.

## Supplementary Material

Supplementary Informations
