# Supplementary material for: Transcriptomic variation of pharmacogenes in multiple human tissues and lymphoblastoid cell lines
Source: Pharmacogenomics J. 2016 Feb 9;17(2):137–45. doi: 10.1038/tpj.2015.93 (PMC4980276; doi:10.1038/tpj.2015.93)
Supplement: Supplementary Informations [file tpj201593x1.pdf]

Supplemental Methods

Tissue Collection and RNA Isolation

Liver, kidney, and heart tissue samples come from frozen tissue samples (post-mortem or from surgical resection) from tissue banks as previously described[1-3] (Supplemental Table 2). Where tissues were collected from surgical resection for tumor biopsy, normal tissues adjacent to the tumor were isolated. Kidney samples come from a mix of normal tissue extracted during biopsy for diagnosis of renal carcinoma or taken post-mortem from individuals with no known renal dysfunction; samples were flash frozen (except 1 which was formalin fixed and paraffin embedded). Heart samples come from patients with no known cardiac disorder. Disease states of the patients who donated the liver samples are not known, but the organs themselves were healthy. Heart and liver samples were all flash frozen.

Adipose tissues and LCLs were collected from subjects who participated in clinical trials (Cholesterol and Pharmacogenetic Study, NCT00451828 and Dietary Protein and Insulin Sensitivity Study, NCT00508937) as previously described [1, 4]. All participants in the trials provided written informed consent, and protocols were reviewed by the appropriate institutional review boards at the University of California, Los Angeles, School of Medicine (Los Angeles, CA) or San Francisco General Hospital (San Francisco, CA) (Cholesterol and Pharmacogenetic Study) and Children's Hospital & Research Center (Oakland,CA) (Dietary Protein and Insulin Sensitivity Study). Adipose tissue biopsy specimens were collected from human participants in a dietary intervention clinical trial (NCT00508937) (unpublished) during the control diet (55% carbohydrate, 30% fat). Participants were non-smoking and overweight to obese with BMI between 25 and 40 kg/m^2 but otherwise healthy. Adipose tissue was collected by needle biopsy from the subcutaneous flanking region after injection of 1% lidocaine hydrochloride with adrenaline and sodium bicarbonate.

Biopsy samples were washed with phosphate-buffered saline, flash frozen in liquid nitrogen, and stored at -80°C.

Immortalized lymphoblastoid cell lines (LCLs) were derived from blood samples isolated from participants of the Cholesterol and Pharmacogenetics (CAP) clinical trial (NCT00451828), and grown at 37°C with 5% CO_2 in RPMI 1640 media supplemented with 10% FBS, 500 U/ml penicillin/streptomycin, and 2 nmol/L GlutaMAX (Life Technologies). All participants had elevated baseline total serum cholesterol levels (160 to 400 mg/dl) who were not taking any lipid lowering medication, corticosteroids, immunosuppressive drugs, or any drugs known to affect the CYP3A4 system or had known liver disease or dysfunction, uncontrolled hypertriglyceridemia, blood pressure, or diabetes mellitus, abnormal renal or thyroid function, any other recent major illness, or known alcohol or drug abuse.

Methods for RNA isolation from heart, liver, kidney tissue, and lymphoblastoid cell lines were previously described [1-3, 5-7]; any changes from published methods are noted below.

RNA isolation from human adipose tissue

Total RNA was isolated from 0.5-1.0g frozen adipose tissue using the TRIzol Plus RNA Purification System (Ambion). Tissue disruption was performed using the Thermo Savant FastPrep FP120 Homogenizer with each sample run twice at 5 m/s for 40 seconds and stored on ice between homogenizations. An on-column digestion of DNA during RNA purification was performed using the RNase-Free DNase Set (Qiagen). Total RNA was quantified by spectroscopy by using an ND-1000 NanoDrop spectrophotometer (NanoDrop Technologies). Total RNA quality was assessed by using the RNA 6000 Nano Kit on a 2100 Bioanalyzer (Agilent). All samples recovered >6µg of total RNA and achieved an RNA Integrity Number (RIN) of at least 6.5 or higher.

RNA isolation from human lymphoblastoid cell lines

Total RNA was isolated from LCLs using the PureLink RNA Mini Kit (Life Technologies). An on-column digestion of DNA during RNA purification was performed using the RNase-Free DNase Set (Qiagen). Quantification and quality control of total RNA was performed as described above.

Preparation for RNA-sequencing Library

RNA integrity number (RIN) was quantified for all extracted RNA by Bioanalyzer (Agilent), and only samples with RIN scores ≥ 6.0 were used for library preparation. RNA-seq libraries from liver, kidney, adipose, and heart samples were prepared and sequenced at the Baylor College of Medicine Human Genome Sequencing Center (BCM-HGSC). Libraries were prepared as outlined by Zhong and colleagues[8] with modifications and added quality checks; library preparation was generally conducted for all samples from a given tissue at one time but different tissues' samples were prepared in distinct batches. RNA-seq libraries from liver, kidney, adipose, and heart samples were prepared as follows. In brief, 0.5-1.0 µg of total RNA was twice selected for mRNA by oligo (dT) and then fragmented by heating. First strand cDNA was synthesized using Superscript III reverse transcriptase and random hexamer primers. After second strand synthesis by DNA polymerase I and with dUTP in place of dTTP, double stranded cDNA was end-repaired and A-tailed prior to ligation of Illumina adaptors including DNA indices. Libraries were made strand-specific by digestion with Uracil-DNA Glycosylase prior to PCR amplification. Bead-based clean-up was incorporated after each enzymatic reaction and libraries were checked by flash gel and Bioanalyzer analysis prior to pooling, cluster formation and paired end sequencing on Illumina HiSeq sequencing instruments at five samples per lane.

RNA-seq libraries from LCLs were prepared at LabCorp (formerly Covance, Seattle, WA) and sequenced at the University of Washington Northwest Genomics Center. cDNA libraries were prepared using a similar protocol to the tissues using the TruSeq RNA Sample Prep Kit v2. The corresponding vendor protocol was used for almost all steps (including polyA selection) with a final PCR enrichment using 15 cycles. The protocol was modified to increase fragment size and to yield final libraries containing only the first strand-synthesized product by: (a) reduction of elute-prime-frag treatment time from 8 minutes to 4 minutes (to increase library insert size), (b) second strand synthesis using a mastermix containing dUTP (in place of dTTP) and RNAse H (to remove RNA), and (c) following the final adapter ligation cleanup, libraries were digested with uracil DNA deglycosylase (to remove dUTP-containing second strand product).

Quality Control and Alignment

Raw reads were mapped to the human genome sequence (hg19)[9] using Tophat v2.0.6[10], allowing for four mismatches per 100bp read, a maximum of 6 edit distances per read, and one mismatch in the splice anchor region. Insert sizes were estimated using the Picard Tools[11] (v1.79) tools SortSam.jar and CollectInsertSizeMetrics.jar on Bowtie2(v2.0.0-beta7)[12]-generated sam files from a subset of 500,000 paired-end reads per sample (bowtie2 options -q --very-fast --phred33).

Using Picard Tools, the alignment files were sorted by genomic coordinates, read-group data was added, and duplicate reads were marked and removed. For some samples, where initial quality control suggested low read counts, stored libraries were sequenced again, and reads from all runs for a sample were merged together. In addition, several quality metrics were calculated using RNA-SeQC[13]. Selected metrics are shown in Supplemental Figure S8. All QC metrics fall within acceptable ranges, though a one-way analysis of variance F-test

indicates significant differences between tissue types for all metrics. Investigating all pairwise comparisons by Tukey's HSD test reveals the largest magnitude difference between LCL libraries and other tissues (Supplemental Table S10). Given that library preparation for LCLs was conducted at a different site using slightly different protocols, this result is not unexpected. Between physiological tissues, slight but significant differences were observed between groups in intragenic, intergenic, and rRNA rates (difference of means < 0.01), coverage, and mismatch rates (difference of means < 0.01). These subtle differences may be caused by differences in RNA extraction and library preparation stages. While these steps were conducted using the same protocols for the four physiological tissues, technicians and dates for preparation varied. The upper-quartile normalization and subsampling procedures applied correct for such differences between samples.

While others have attempted to use statistical methods to correct for any hidden biases in the data[14, 15], in this study any potential technical confounders (sample source, extraction method, technician, etc.) are conflated with sample type and any attempt to correct for such biases in our data would also remove biologically meaningful tissue-specific effects. However, to ensure there were no systematic biases in our samples due to sample preparation or sample source, we checked variability of expression values and absolute expression values for a set of seven genes that have previously been determined to show consistent expression patterns across tissues using the same metrics for variability of expression employed by the original study [16]. In our dataset, these seven genes showed low variability in expression values across individuals (standard deviation of $\log_2$(FPKM) across all samples in a given tissue type < 0.5) and consistent expression levels between the physiological tissues (fold change of tissue-specific expression relative to overall geometric mean of expression < 2) (Supplemental Table S11). The overall distribution of protein coding gene expression levels was also similar between the four physiological tissues (Supplemental Figure S4A,C). Further, as expected, principal components

analysis on protein coding gene FPKMs (FPKM>1) by sample showed clear separation between the sample types (Supplemental Figure S5A). Finally, while most of the physiological tissue samples were flash frozen, one kidney tissue sample was formalin fixed and paraffin embedded (FFPE) for storage. A principal components analysis on PGRN pharmacogene FPKMs in kidney samples suggested that the tissue storage method (flash frozen vs FFPE) did not explain a significant portion of variability in pharmacogene expression.

Transcriptome Analyses

Transcript structure assembly was performed with Cufflinks (v.2.0.2)[17] on each sample for each tissue type. The Gencode v12 annotation[18] was used as a reference to guide assembly (--GTF-guide). Additional parameters included upper-quartile normalization (--upper-quartile-norm), library type (--library-type fr-firststrand), and maximum bundle length (--max-bundle-length 7500000). Cuffmerge (v2.0.2) was then used to merge all the cufflinks assemblies and the reference annotation into one large set of transcript structures including those both known and novel. For pharmacogenes, to increase confidence in the discovery of transcript structures not previously annotated, splice junctions not present in the reference annotation were required to pass a Shannon entropy score [19] threshold of 2 (Supplemental Figure S9). The entropy score is

$$-\sum_{i=1}^{n} p(x_i)\log_2 p(x_i)$$

where $p(x_i)$ is the proportion of junction reads starting at position *i* and *n* is the total number of different starting positions (offsets) for reads crossing this junction. Our scores were calculated using all the reads in this study and an entropy score of 2 means that the reads crossing the junction start at four different positions.

The mapped reads for each sample were subsampled down to 20 million using multiple runs of the Picard tool DownsampleSam. Only 18 kidney samples had enough reads, thus 18 samples were chosen from the other four tissue types at

random (except adipose tissue, where instead samples from the African-American and Asian patients were excluded, leaving just samples from Caucasian patients and those with unknown ethnicity because the individuals for the other tissues were all Caucasian).

To calculate the expression level of each gene in each tissue type, Cuffdiff (v2.2.1)[17] was run with default parameters and the option --library-type fr-firststrand on the subsampled read files with 18 samples per tissue type used as 'replicates' and the merged set of transcript structures used as the reference annotation. Gene expression for each tissue was calculated by summing all isoforms for a given gene in a given tissue from the isoforms.fpkm_tracking file generated by Cuffdiff. This file combines all individuals to give one value per isoform per tissue reported for each gene. Genes with any isoform with a status of "HIDATA" do not have a calculated FPKM value. Two pharmacogenes, ALB (albumin) and SERPINA1 (serpin peptidase inhibitor, clade A), in the liver had HIDATA values. Gene expression for each individual was calculated by summing all isoforms for a given gene for each individual from the isoforms.read_group_tracking file generated by Cuffdiff. This file has, for each individual, one value per isoform reported for each gene.

Differential expression between tissues was calculated using Cuffdiff (v2.1.1)[17] and DESeq (v2.13)[20]. Differential expression was extracted from the gene_exp.diff file generated by Cuffdiff. To prepare read files for DESeq, read counts were generated with HTSeq (v0.5.4p3)[20] using reverse stranded orientation (-s reverse) and counting mode "intersection-strict" on subsampled bam files. The Cuffmerge GTF described above for Cuffdiff analysis was used as reference GTF. For the DESeq analysis, all commands were run with defaults on all arguments, except for estimateDispersions, which was run with method="per-condition" and fitType="local". Differentially expressed genes were defined as those with qvalue less than 0.1 in both DESeq and Cuffdiff and greater than or equal to 2-fold change in expression between tissues.

Clustering of gene expression values across samples was conducted using *K*-means clustering as implemented in BiCAT[21] based on the Euclidean distance between $\log_2$ transformed FPKM values (FPKM<1 set to 1) with up to 1000 iterations and 23 clusters. Euclidean distance was chosen as the goal was to group genes by overall expression level across samples. The number of clusters (k) was selected by studying the within group sum of squares at increasing numbers of clusters. Because cluster assignment can be sensitive to choice of k, cluster assignment of genes highlighted in Figure 2B-2F was also evaluated with alternate choice of k (selected values between 6 and 20). For clusters highlighted in 2 B, C, E, and F, all genes that clustered together at k=23 also clustered together with smaller numbers of clusters. For the cluster highlighted in 2D (genes expressed at higher levels in liver than the other 4 tissues), in some cases genes were split between two different clusters representing genes very highly expressed in liver and those more moderately expressed in liver (but still higher than in the other four tissues).

To evaluate variability in gene expression values, the coefficient of variation (CV) by tissue for all genes with median FPKM>1 was calculated. Enrichment of variability in gene expression in gene sets was calculated using the Gene Set Enrichment Analysis (GSEA v.2.1.0)[22, 23] PreRanked tool (classic algorithm), with CV of each gene in a particular tissue type used as gene ranking. Gene ontology biological process sets (from the Molecular Signatures Database v4.0 provided along with the GSEA tool) with set size between 15 and 500 genes and pharmacogenes were tested. Significance of enrichment was calculated by permuting gene-rank associations 1000 times.

Splice junctions were identified using the JuncBASE package[19] on reads that overlapped protein-coding genes. Only splice junctions with a Shannon entropy score greater than 2 using all subsampled reads were used. Junctions were called non-annotated if they were not present in the Gencode v12 annotation nor

in the Ensemble annotation. Using additional code generated by the authors, the reported JuncBASE results were transformed into groups of mutually exclusive junction sets, each with defined length-normalized read counts (reads/100bp) and ‘percent spliced in’ (PSI) values. Complex events (e.g. cassette exon + alternate 3’ splice site) with ambiguously mapped reads were filtered out. Intron retention events made up a large fraction of the reported splice events but are difficult to confidently annotate as alternative splicing as opposed to pre-mRNA contamination or other technical artifacts and so were also filtered out. The novel last exon event of SLC22A7 was not reported by JuncBASE. The PSI values were calculated using only the junction read counts for the novel junction and the known junction.

Code availability

Scripts used to process the output from JuncBASE are deposited at https://github.com/cefrnch/PGRN_JuncBASE_postprocessing.

Validation of exon junctions and PSI estimates

For PCR validation of exon junctions, primers were designed to detect the junctions and cloned into TOPO® TA vector (pCR™2.1-TOPO® vector) (Invitrogen, CA) to confirm the sequence. Commercially available purified total RNA from normal human tissues (Ambion®, CA) were used for cDNA synthesis by Superscript III cDNA synthesis kit using Oligo(dT) (Invitrogen, CA) and GoTaq® DNA polymerase (Promega, MI) for PCR amplification. For qPCR validation of PSI values, cDNA was synthesized using the ABI cDNA Archive kit and *HMGCR13(-), HMGCR13(+), LDLR4(-)* and *LDLR4(+)* were quantified by TaqMan assay in triplicate as previously described[24]. PSI values were calculated from the qPCR and RNA-seq measurements as the ratio of *HMGCR13(-)/13(+)* and *LDLR4(-)/4(+)*, quantile normalized, and tested for correlation using a general linear model in JMP 9.0 (SAS Institute).

Comparison with Additional RNA-Seq Studies

To validate the patterns of pharmacogene expression and splicing identified in this study, we analyzed data from the Genotype Tissue Expression (GTEx) Project[25].

Expression (RPKM, mapped reads per kilobase per million mapped reads) values per individual, per gene for the GTEx analysis v4 were downloaded from the GTEx portal (http://gtexportal.org). Tissue types were matched to those used in the PGRN RNASeq analysis - adipose (subcutaneous, 128 samples), cells (ebv transformed lymphocytes, 54 samples), heart (left ventricle, 95 samples), liver (34 samples), and kidney (cortex, 8 samples). Both datasets used Tophat for alignment with Gencode(v12) as a transcript model. Downstream processing steps differed between the two datasets.

For each tissue type, mean, median, and coefficient of variance of RPKMs were calculated for each pharmacogene. Variability of gene expression in gene sets (using coefficient of variation as a gene ranking metric in a gene set enrichment analysis as described for the PGRN dataset) was evaluated for each tissue type. Correlation between pharmacogene expression values and coefficient of variation in the PGRN and GTEx sets was calculated using spearman's rank correlation.

For each of 'Adipose - Subcutaneous', 'Heart - Left Ventricle', 'Liver', and 'Cells - EBV-transformed lymphocytes', 18 samples were chosen at random from the set of samples included in the GTEx release phs000424.v4.p1. For 'Kidney - Cortex', only 8 samples were available and all were used. The aligned reads were downloaded from SRA/dbGaP, subsampled down to 20 million reads per sample, and the 80 samples were run through the JuncBASE pipeline in the same way as was done for the PGRN data.

Supplemental Results

Global transcriptome analysis

Across the samples, 56% of all genes were expressed with FPKM ≥1 in at least one individual (Supplemental Table S3B). Of these expressed genes, 70% (7,845) were expressed with FPKM ≥1 in all 90 individuals. A small number (~2%) of protein coding genes were completely undetectable in our dataset (no FPKM reported).

We saw evidence for alternative splicing for 35,722 events in 13,833 genes (i.e., for each potential alternative splice event, multiple mutually exclusive junctions had a PSI value ≥5 and had ≥1 read/100bp). We also observed 4,192 previously not annotated splice events supported by the more restrictive read coverage threshold of 5 reads/100bp and present with PSI ≥5 in at least one sample (Supplementary Figure S7A); less conservatively, 20,128 non-annotated junction sets have read coverage of at least 1 read/100bp (Supplemental Figure S7B).

The total number of splice events observed in at least one individual with at least 5 reads/100bp depends on the number of samples considered, especially when considering fewer than 10 samples (Supplementary Figure S6C), due to the variability of gene expression and alternative splicing amongst individuals. With only one sample, we would see as few as 60% of the splice events found in at least one sample of 18 samples. However, with 18 samples per tissue type we are able to see around 95% of the splice events we would see if we used all the samples for a given tissue type in this study. As expected, the ability to detect splice events also depends on read depth. We tested four subsampling depths (10, 20, 30, and 40 million reads) and find an additional 22-34% of junction sets for a given sample (e.g. LCLs and kidney in Supplementary Figure S6A, B) at 40 million reads compared to 20 million reads.

Association of gene expression with age and gender

Association of pharmacogene expression values with age and gender in each tissue type were calculated by linear regression and Mann-Whitney test, respectively, and are reported where the false discovery rate adjusted p-value is less than or equal to 0.05. Age of patient was associated with pharmacogene expression for two genes, albeit at very low levels of expression – *CACNA1S* in the liver and *CYP2B7P1* in the kidney. *CACNA1S* expression was under FPKM 0.025 in all samples, but slightly and significantly higher in liver samples from two patients under age 20 (15 and 16 years) compared to liver samples from remaining patients (≥ age 45). Similarly, *CYP2B7P1* expression was under FPKM 0.025 in all samples, but slightly and significantly higher in kidney samples from two patients under age 10 compared to kidney samples from remaining patients (≥ age 38). No association between expression and sex of patients was identified.

Comparison with Additional RNA-Seq Studies

To validate the patterns of expression and splicing observed in this dataset with those identified in a second RNA-Seq study, expression values (RPKM) per gene/individual and junction counts per individual were extracted from the Genotype Tissue Expression (GTEx) Project for EBV transformed lymphocytes (LCLs) and kidney (cortex), liver, adipose (subcutaneous), heart (left ventricle) tissues.

For the subset of pharmacogenes highlighted in Figure 3, we examined variability in expression across individuals in the GTEx dataset (Supplemental Figure S10), and observe broadly similar trends in variability across individuals. For example, in the GTEx dataset, as in the PGRN dataset, we observe high variability in expression of the cytochrome P-450 enzymes in the liver, and consistent levels of expression across individuals in all tissues for glutathione S-transferase enzymes. In fact, our data seem more tightly clustered for these more consistent

genes, suggesting the heterogeneity from live biopsies may be less significant than post-mortem issues. Across all pharmacogenes, the Spearman correlation (Spearman rho) between coefficient of variation for PGRN and GTEx datasets was greater than 0.5 (Kidney, 0.68; Heart, 0.88; Liver, 0.51; Adipose, 0.80; LCLs, 0.52).

Further, in this secondary dataset, as in the PGRN dataset, pharmacogenes are among the 10 most variably expressed gene sets (compared to Gene Ontology biological process sets) in kidney, liver, adipose, heart, and LCLs. As in the PGRN dataset, as a group, pharmacogenes were expressed at higher levels in liver and lower levels in LCLs. Further, 107 of 133 of the set of pharmacogenes identified as having tissue-specific expression patterns (defined as ten times higher or lower expression in one of the tissues relative to all others in the PGRN dataset) (Supplemental Table S5) also showed the same tissue specific expression patterns in the GTEx dataset. Finally, for all tissue types, the correlation between pharmacogene expression values in the PGRN and GTEx datasets was greater than 90% (Spearman rho: Kidney, 0.91; Liver, 0.92; Adipose,0.92; Heart, 0.96; LCLs,0.91).

We downloaded the aligned reads for 18 GTEx samples per tissue (except kidney where there is only 8 samples total), subsampled to 20 million reads per sample, and performed the same JuncBASE splicing analysis as was done for the PGRN data. Of the 278 pharmacogenes reported as producing multiple isoforms in our data, 206 were also clearly alternatively spliced in the GTEx data (multiple mutually exclusive junctions each with at least 1 read/100bp and PSI>5 in at least one sample). Of the other 72, most were inconclusive in the GTEx data. Only 11 pharmacogenes were alternatively spliced in the GTEx samples, but were inconclusive (6) or had no evidence of alternative splicing (5) in the PGRN data. Many pharmacogenes that are significantly differentially spliced between tissues in the PGRN data are also differentially spliced in the GTEx data (Supplemental Table S12), though for the comparisons involving kidney or LCLs,

the overlap is low. For kidney, this likely is partly due to there only being 8 GTEx samples, limiting power for detecting significant splicing.

We also evaluated whether the splice events that we observed in only one tissue in the PGRN data (Figure 4A) were found in the other tissues' GTEx samples. For each tissue, 64-100% of the events that we checked were also not found in the other four tissues in the GTEx samples (Supplemental Table S13). The novel alternative last exon in *SLC22A7* (Figure 4C) was also observed in the GTEx samples, with junction coverage >40 reads/100bp in 16 samples. Additionally, of the 183 novel splice events we report in pharmacogenes, 54 were picked up by the JuncBASE analysis of GTEx data.

Supplemental Figure Legends

Supplementary Figure S1. Validation of non-annotated junctions. PCR and sequencing were performed to validate the non-annotated junctions in SLC22A7 (Lane 1) and APOA2 (Lane 3 and 5). Pooled liver cDNA (20 ng) was used in the PCR reactions. Samples in Lane 2, 4 and 6 are PCR reactions with primers alone and without cDNA. Sample in Lane 7 is PCR reaction without primers and cDNA.

Supplementary Figure S2. Validation of PSI values obtained by RNA-seq. Percent spliced in (PSI) values were quantified by RNA-seq and qPCR for *HMGCR* exon 13 skipping (A) and *LDLR* exon 4 skipping (B) from RNA derived from 39 LCLs. Both RNA-seq and qPCR PSI values were calculated as the quantile normalized ratio of the alternatively spliced/canonical transcript ratio.

Supplemental Figure S3. Gene expression (FPKM) by sample across each tissue type and LCLs for 389 PGRN pharmacogenes from subsampled data (18 samples per tissue type, 20 million reads per sample). The black dot indicates median FPKM per gene and tissue type. Plots drawn using R package ggplot2. [26]

Supplemental Figure S4. Distribution of FPKM values for (A) protein coding genes in each individual, (B) PGRN pharmacogenes in each individual, (C) protein coding genes in each tissue, (D) PGRN pharmacogenes in each tissue. Gene FPKM values are calculated as the sum of FPKM values for all isoforms of the gene. Tissue gene expression in (C) and (D) is reported by Cuffdiff when all samples for that tissue are used as replicates.

Supplemental Figure S5. Principal components analysis on gene expression (log_2(FPKM)) across individuals for (A) protein coding genes and (B) pharmacogenes. The first four principal components are shown here. Principal components analysis conducted using function prcomp in R package Stats. [27]

Supplemental Figure S6. The number of splice events observed in **(A)** LCLs or **(B)** kidney with increasing read depth and number of samples used. The x-axis is the number of samples considered (subsampled down from the total number of samples for the tissue). Each point is the number of splice events observed with read coverage greater than 5 reads/100bp in at least one of the samples used, averaged after 100 permutations of subsampling. The error bars are standard deviations for the permutations. **(C)** The number of splice events observed with 20 million reads with increasing number of samples studied, by tissue.

Supplemental Figure S7. (**A**) The distribution of the number of previously non-annotated splice events observed in at least one sample (of 90 samples) with PSI greater than the threshold. All events also have read coverage of at least 5 reads/100bp. (**B**) The distribution of the number of non-annotated splice events

observed in at least one sample (of 90 samples) with read coverage greater than the threshold.

Supplemental Figure S8. Post-alignment QC metrics by tissue generated by RNAseqQC.

Supplemental Figure S9. Distribution of Shannon entropy scores for splice junctions found in the Gencode or Ensembl annotations ('known') and those that are not ('novel'). Entropy scores take into account read coverage and number of offsets (read start positions, Supplementary Methods), and were calculated after merging all reads for all samples in our data set. An entropy score cutoff of 2 was used to define 'confident' non-annotated junctions.

Supplemental Figure S10. Gene expression (RPKM) by sample across each tissue type (adipose, n=128; heart, n=95; liver, n=34;kidney, n=8) and LCLs (n=54) for selected cytochrome P450 (CYP) enzymes, solute carrier family (SLC) transporters, and other pharmacogenes discussed in this article from the Genotype-Tissue Expression project[25] (GTEx, v4). The black dot indicates median FPKM per gene and tissue type. Plots drawn using R package ggplot2.[26]

Supplemental References

1. Simon JA, Lin F, Hulley SB, Blanche PJ, Waters D, Shiboski S*, et al*. Phenotypic predictors of response to simvastatin therapy among African-Americans and Caucasians: the Cholesterol and Pharmacogenetics (CAP) Study. *The American journal of cardiology* 2006; **97**(6)**:** 843-850.

2. Dahlin A, Geier E, Stocker SL, Cropp CD, Grigorenko E, Bloomer M*, et al*. Gene expression profiling of transporters in the solute carrier and ATP-binding cassette superfamilies in human eye substructures. *Molecular pharmaceutics* 2013; **10**(2)**:** 650-663.

3. Schadt EE, Molony C, Chudin E, Hao K, Yang X, Lum PY*, et al*. Mapping the genetic architecture of gene expression in human liver. *PLoS biology* 2008; **6**(5)**:** e107.

4. Chiu S, Williams PT, Dawson T, Bergman RN, Stefanovski D, Watkins SM*, et al*. Diets high in protein or saturated fat do not affect insulin sensitivity or plasma concentrations of lipids and lipoproteins in overweight and obese adults. *The Journal of nutrition* 2014; **144**(11)**:** 1753-1759.

5. Chaudhry AS, Thirumaran RK, Yasuda K, Yang X, Fan Y, Strom SC*, et al*. Genetic Variation in Aldo-Keto Reductase 1D1 (AKR1D1) Affects the Expression and Activity of Multiple Cytochrome P450s. *Drug metabolism and disposition: the biological fate of chemicals* 2013; **41**(8)**:** 1538-1547.

6. Lundquist AL, Manderfield LJ, Vanoye CG, Rogers CS, Donahue BS, Chang PA*, et al*. Expression of multiple KCNE genes in human heart may enable variable modulation of I(Ks). *Journal of molecular and cellular cardiology* 2005; **38**(2)**:** 277-287.

7. Innocenti F, Cooper GM, Stanaway IB, Gamazon ER, Smith JD, Mirkov S*, et al*. Identification, replication, and functional fine-mapping of expression quantitative trait loci in primary human liver tissue. *PLoS genetics* 2011; **7**(5)**:** e1002078.

8. Zhong S, Joung JG, Zheng Y, Chen YR, Liu B, Shao Y*, et al*. High-throughput illumina strand-specific RNA sequencing library preparation. *Cold Spring Harbor protocols* 2011; **2011**(8)**:** 940-949.

9. Karolchik D, Barber GP, Casper J, Clawson H, Cline MS, Diekhans M*, et al*. The UCSC Genome Browser database: 2014 update. *Nucleic acids research* 2014; **42**(Database issue)**:** D764-770.

10. Trapnell C, Pachter L, Salzberg SL. TopHat: discovering splice junctions with RNA-Seq. *Bioinformatics* 2009; **25**(9)**:** 1105-1111.

11. Picard.

12. Langmead B, Salzberg SL. Fast gapped-read alignment with Bowtie 2. *Nature methods* 2012; **9**(4)**:** 357-359.

13. DeLuca DS, Levin JZ, Sivachenko A, Fennell T, Nazaire MD, Williams C*, et al*. RNA-SeQC: RNA-seq metrics for quality control and process optimization. *Bioinformatics* 2012; **28**(11)**:** 1530-1532.

14. Mostafavi S, Battle A, Zhu X, Urban AE, Levinson D, Montgomery SB*, et al*. Normalizing RNA-sequencing data by modeling hidden covariates with prior knowledge. *PloS one* 2013; **8**(7)**:** e68141.

15. Stegle O, Parts L, Durbin R, Winn J. A Bayesian framework to account for complex non-genetic factors in gene expression levels greatly increases

power in eQTL studies. *PLoS computational biology* 2010; **6**(5)**:** e1000770.

16. Eisenberg E, Levanon EY. Human housekeeping genes, revisited. *Trends in genetics : TIG* 2013; **29**(10)**:** 569-574.

17. Trapnell C, Roberts A, Goff L, Pertea G, Kim D, Kelley DR*, et al*. Differential gene and transcript expression analysis of RNA-seq experiments with TopHat and Cufflinks. *Nature protocols* 2012; **7**(3)**:** 562-578.

18. Harrow J, Frankish A, Gonzalez JM, Tapanari E, Diekhans M, Kokocinski F*, et al*. GENCODE: the reference human genome annotation for The ENCODE Project. *Genome research* 2012; **22**(9)**:** 1760-1774.

19. Brooks AN, Yang L, Duff MO, Hansen KD, Park JW, Dudoit S*, et al*. Conservation of an RNA regulatory map between Drosophila and mammals. *Genome research* 2011; **21**(2)**:** 193-202.

20. Anders S, Huber W. Differential expression analysis for sequence count data. *Genome biology* 2010; **11**(10)**:** R106.

21. Barkow S, Bleuler S, Prelic A, Zimmermann P, Zitzler E. BicAT: a biclustering analysis toolbox. *Bioinformatics* 2006; **22**(10)**:** 1282-1283.

22. Mootha VK, Lindgren CM, Eriksson KF, Subramanian A, Sihag S, Lehar J*, et al*. PGC-1alpha-responsive genes involved in oxidative phosphorylation are coordinately downregulated in human diabetes. *Nature genetics* 2003; **34**(3)**:** 267-273.

23. Subramanian A, Tamayo P, Mootha VK, Mukherjee S, Ebert BL, Gillette MA*, et al*. Gene set enrichment analysis: a knowledge-based approach for interpreting genome-wide expression profiles. *Proceedings of the National Academy of Sciences of the United States of America* 2005; **102**(43)**:** 15545-15550.

24. Medina MW, Gao F, Naidoo D, Rudel LL, Temel RE, McDaniel AL*, et al*. Coordinately regulated alternative splicing of genes involved in cholesterol biosynthesis and uptake. *PloS one* 2011; **6**(4)**:** e19420.

25. Consortium GT. Human genomics. The Genotype-Tissue Expression (GTEx) pilot analysis: multitissue gene regulation in humans. *Science* 2015; **348**(6235)**:** 648-660.

26. Wickham H. *ggplot2: elegant graphics for data analysis*. Springer: New York, 2009.

27. Team. RC. *R: A language and environment for statistical computing*. R Foundation for Statistical Computing: Vienna, Austria, 2014.
