# Supplementary material for: Transcriptomic variation of pharmacogenes in multiple human tissues and lymphoblastoid cell lines
Source: Pharmacogenomics J. 2016 Feb 9;17(2):137–45. doi: 10.1038/tpj.2015.93 (PMC4980276; doi:10.1038/tpj.2015.93)
Supplement: Supplementary Figures [file tpj201593x2.pdf]

S1

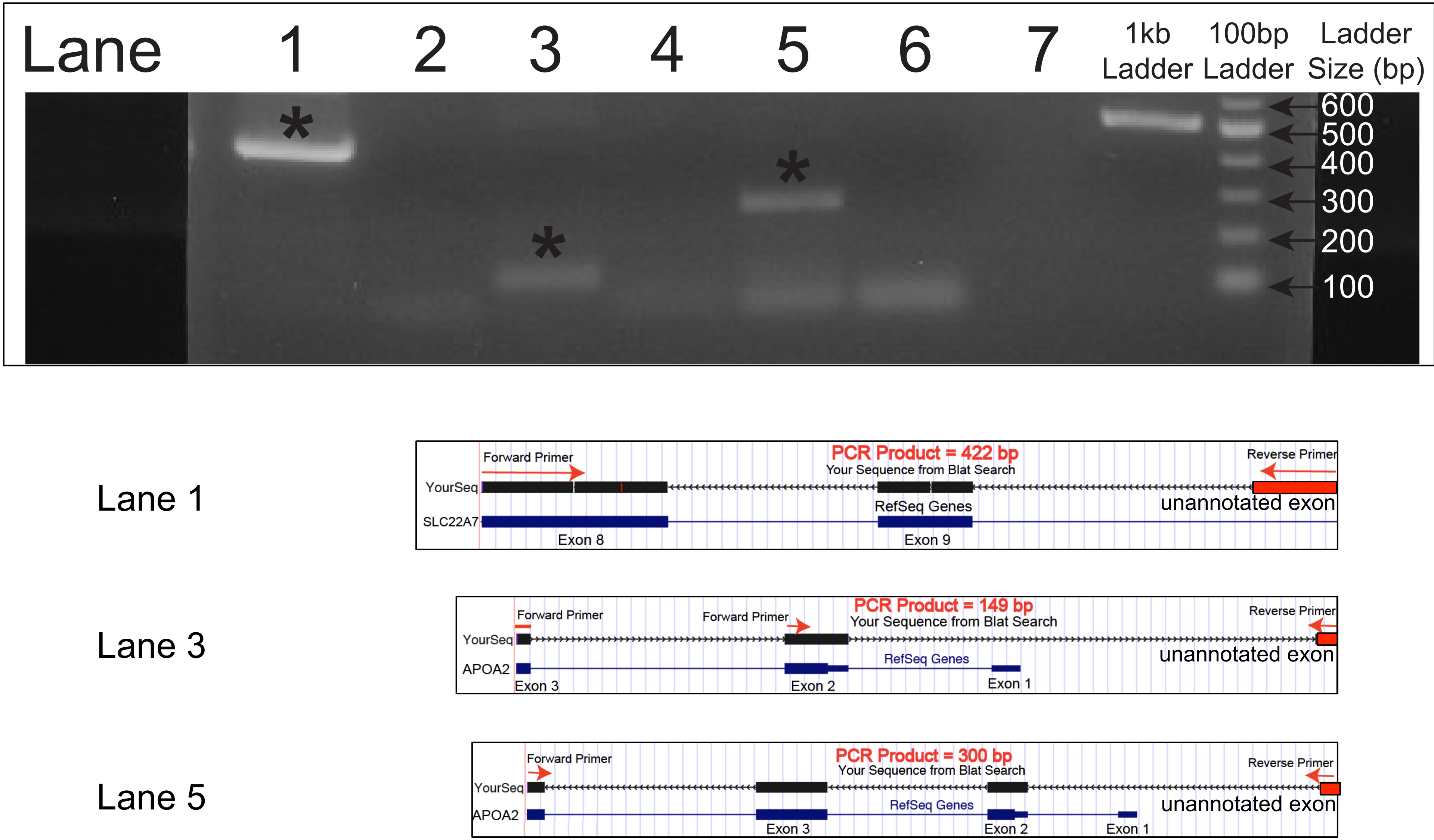

Lane
1
2
3
4
5
6
7
1kb Ladder
100bp Ladder
Ladder Size (bp)
600
500
400
300
200
100
*
*
*
Lane 1
Forward Primer
PCR Product = 422 bp
Your Sequence from Blat Search
Reverse Primer
YourSeq
RefSeq Genes
unannotated exon
SLC22A7
Exon 8
Exon 9
Lane 3
Forward Primer
Forward Primer
PCR Product = 149 bp
Your Sequence from Blat Search
Reverse Primer
YourSeq
RefSeq Genes
unannotated exon
APOA2
Exon 3
Exon 2
Exon 1
Lane 5
Forward Primer
PCR Product = 300 bp
Your Sequence from Blat Search
Reverse Primer
YourSeq
RefSeq Genes
unannotated exon
APOA2
Exon 3
Exon 2
Exon 1

S2

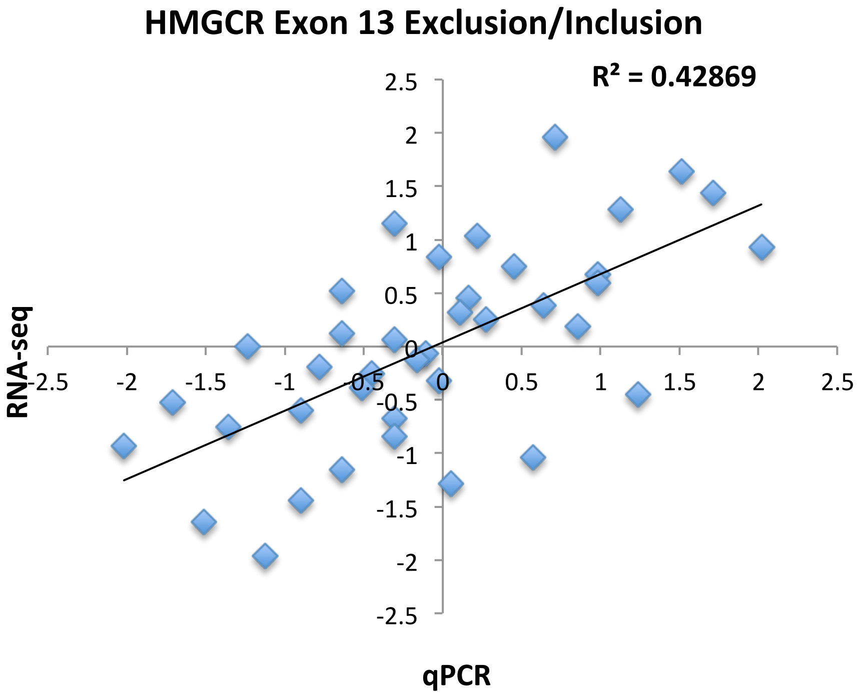
HMGCR Exon 13 Exclusion/Inclusion
R² = 0.42869
RNA-seq
qPCR
2.5
2
1.5
1
0.5
0
-0.5
-1
-1.5
-2
-2.5
-2.5
-2
-1.5
-1
-0.5
0
0.5
1
1.5
2
2.5

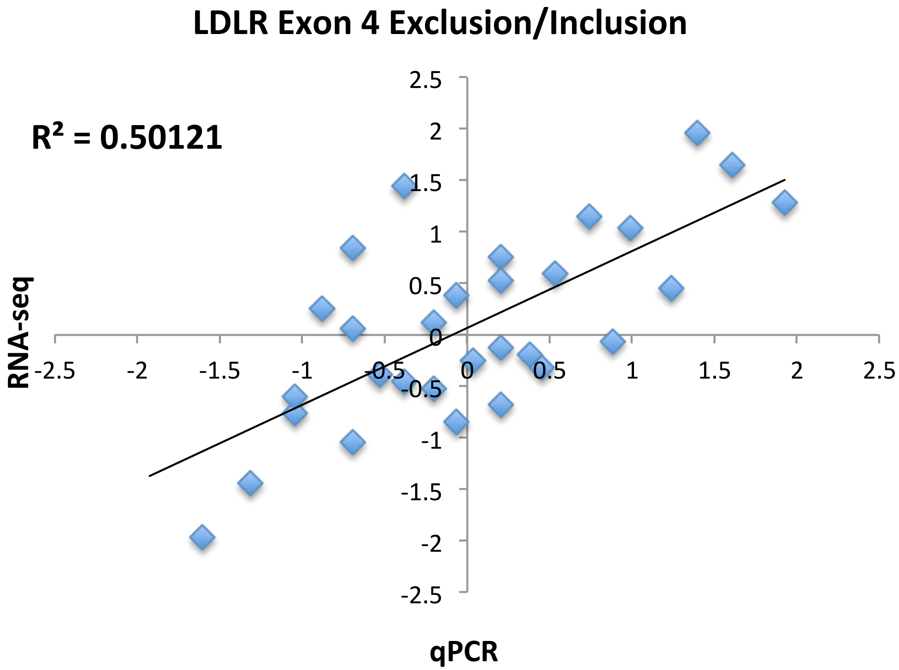
LDLR Exon 4 Exclusion/Inclusion
R² = 0.50121
RNA-seq
qPCR
2.5
2
1.5
1
0.5
0
-0.5
-1
-1.5
-2
-2.5
-2.5
-2
-1.5
-1
-0.5
0
0.5
1
1.5
2
2.5

S3

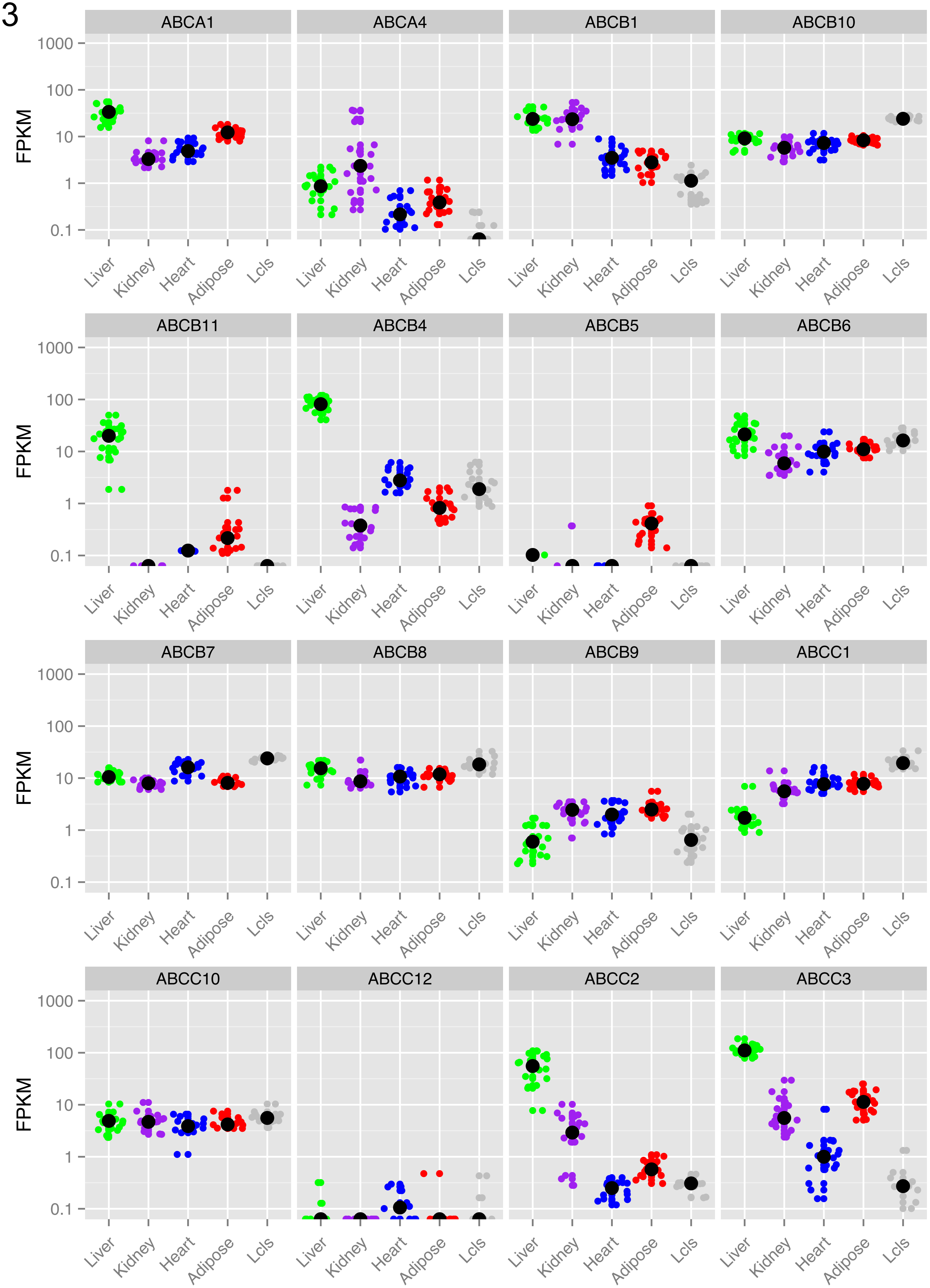
ABCA1
ABCA4
ABCB1
ABCB10
ABCB11
ABCB4
ABCB5
ABCB6
ABCB7
ABCB8
ABCB9
ABCC1
ABCC10
ABCC12
ABCC2
ABCC3
FPKM
1000
100
10
1
0.1
Liver
Kidney
Heart
Adipose
Lcls

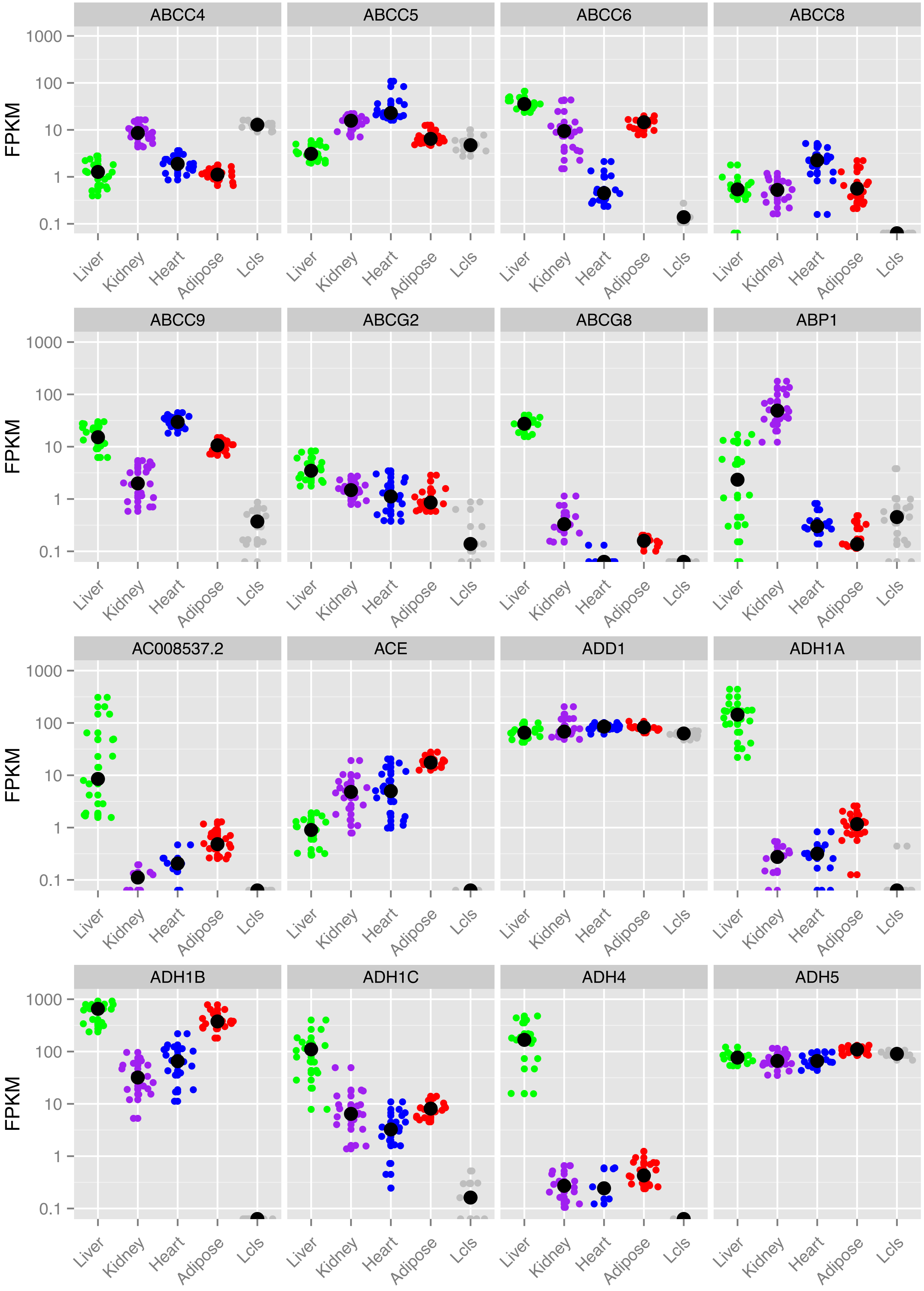

ABCC4
ABCC5
ABCC6
ABCC8
ABCC9
ABCG2
ABCG8
ABP1
AC008537.2
ACE
ADD1
ADH1A
ADH1B
ADH1C
ADH4
ADH5
FPKM
1000
100
10
1
0.1
Liver
Kidney
Heart
Adipose
Lcls

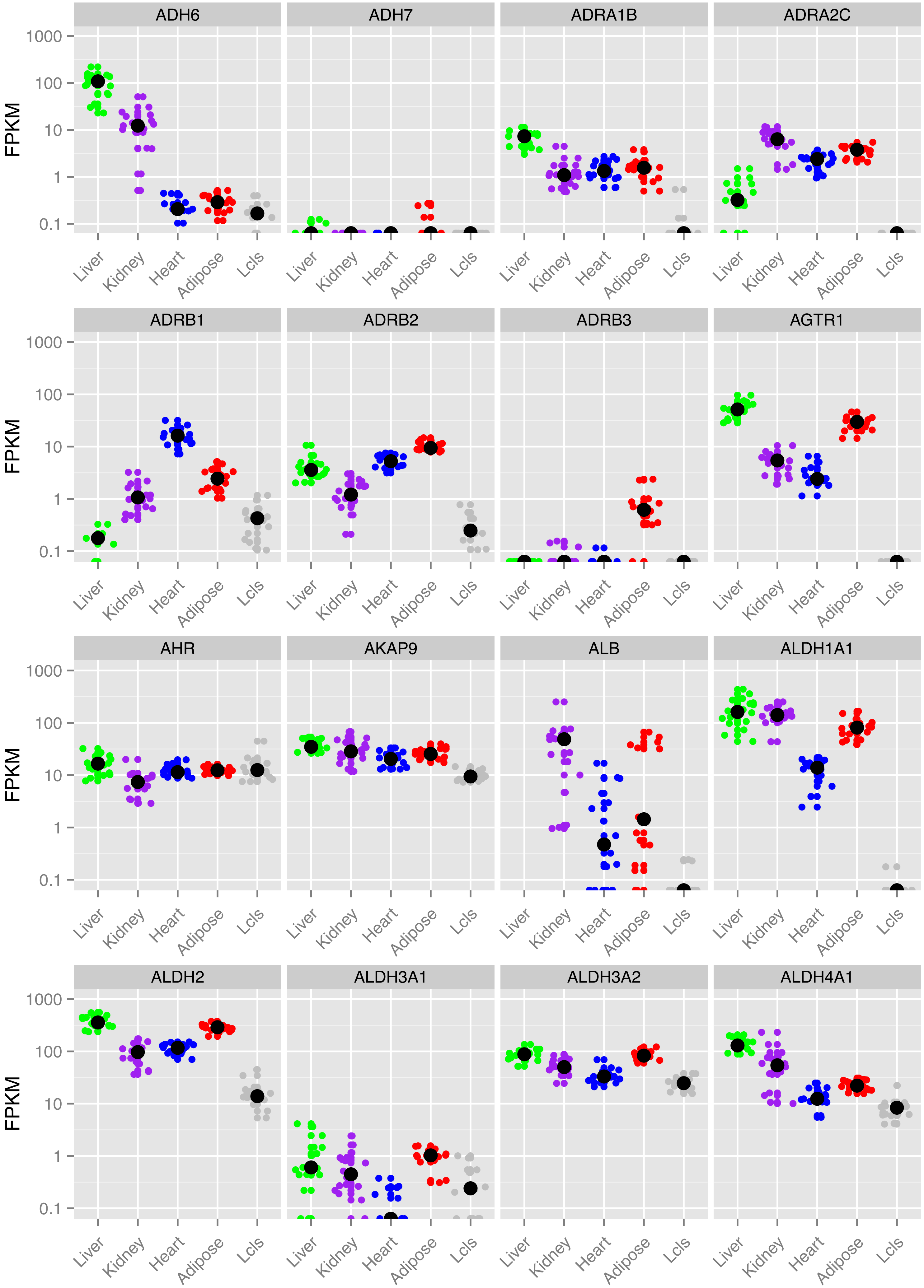
ADH6
ADH7
ADRA1B
ADRA2C
ADRB1
ADRB2
ADRB3
AGTR1
AHR
AKAP9
ALB
ALDH1A1
ALDH2
ALDH3A1
ALDH3A2
ALDH4A1
FPKM
1000
100
10
1
0.1
Liver
Kidney
Heart
Adipose
Lcls

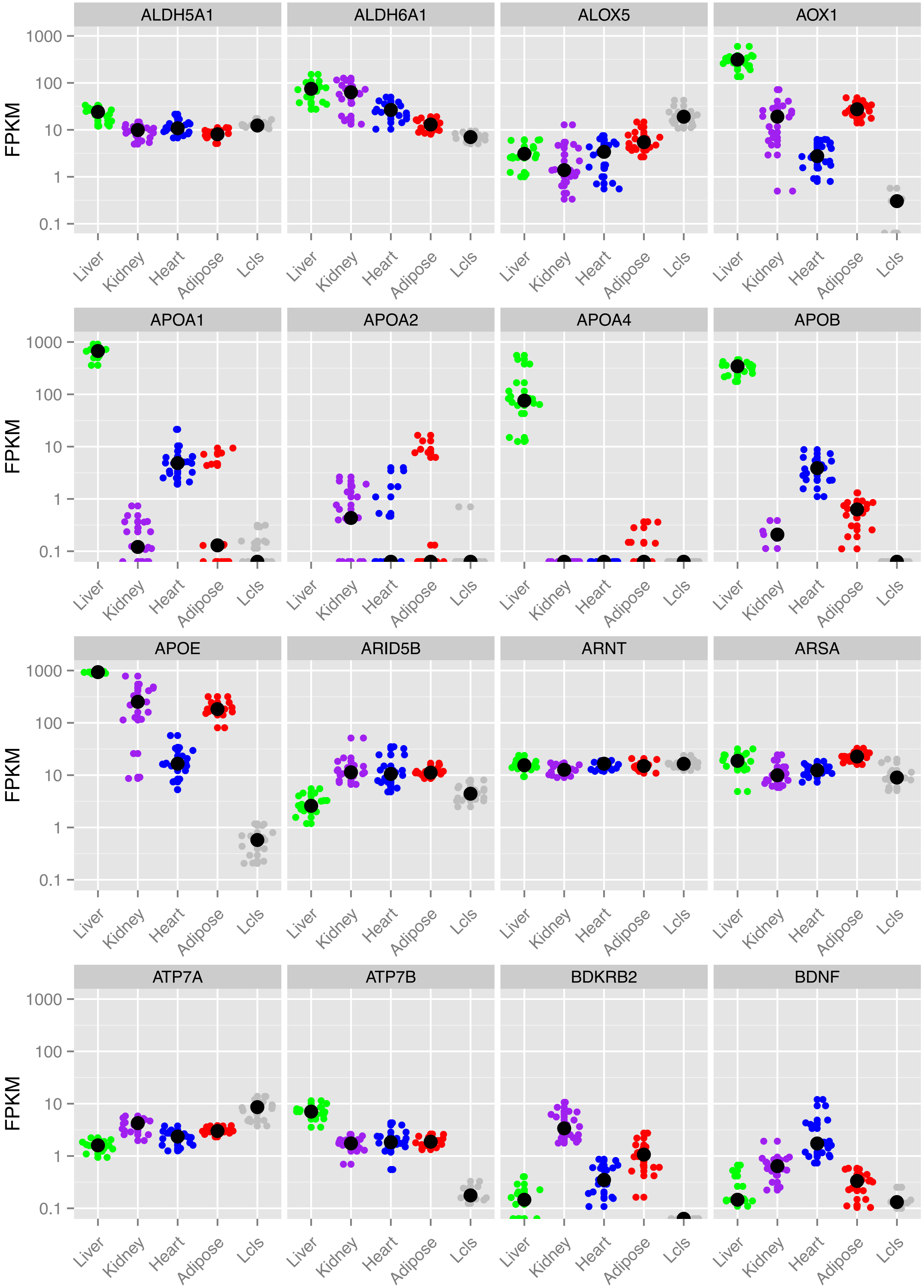
ALDH5A1
ALDH6A1
ALOX5
AOX1
APOA1
APOA2
APOA4
APOB
APOE
ARID5B
ARNT
ARSA
ATP7A
ATP7B
BDKRB2
BDNF
FPKM
1000
100
10
1
0.1
Liver
Kidney
Heart
Adipose
Lcls

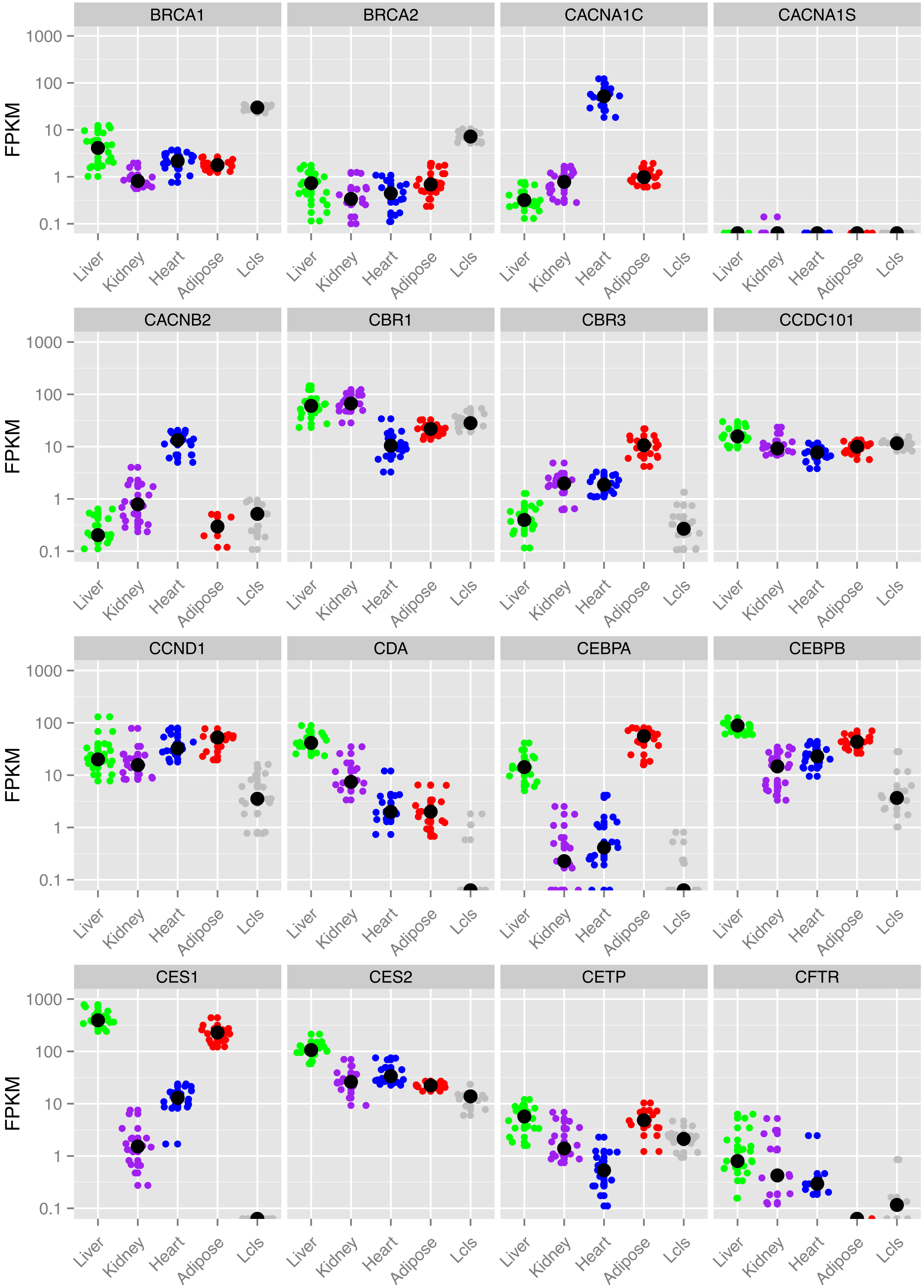
BRCA1
BRCA2
CACNA1C
CACNA1S
CACNB2
CBR1
CBR3
CCDC101
CCND1
CDA
CEBPA
CEBPB
CES1
CES2
CETP
CFTR
FPKM
1000
100
10
1
0.1
Liver
Kidney
Heart
Adipose
Lcls

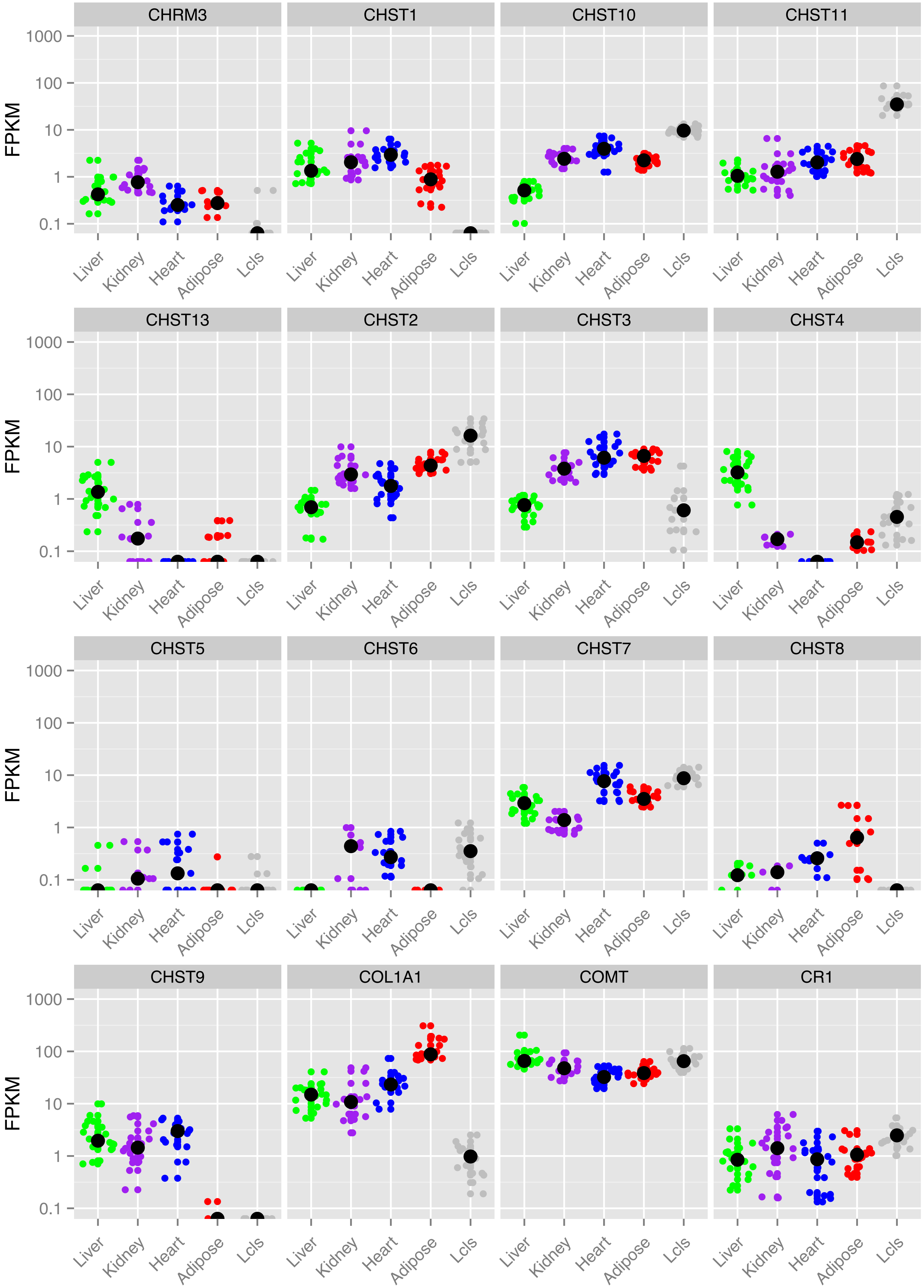
CHRM3
CHST1
CHST10
CHST11
CHST13
CHST2
CHST3
CHST4
CHST5
CHST6
CHST7
CHST8
CHST9
COL1A1
COMT
CR1
FPKM
1000
100
10
1
0.1
Liver
Kidney
Heart
Adipose
Lcls

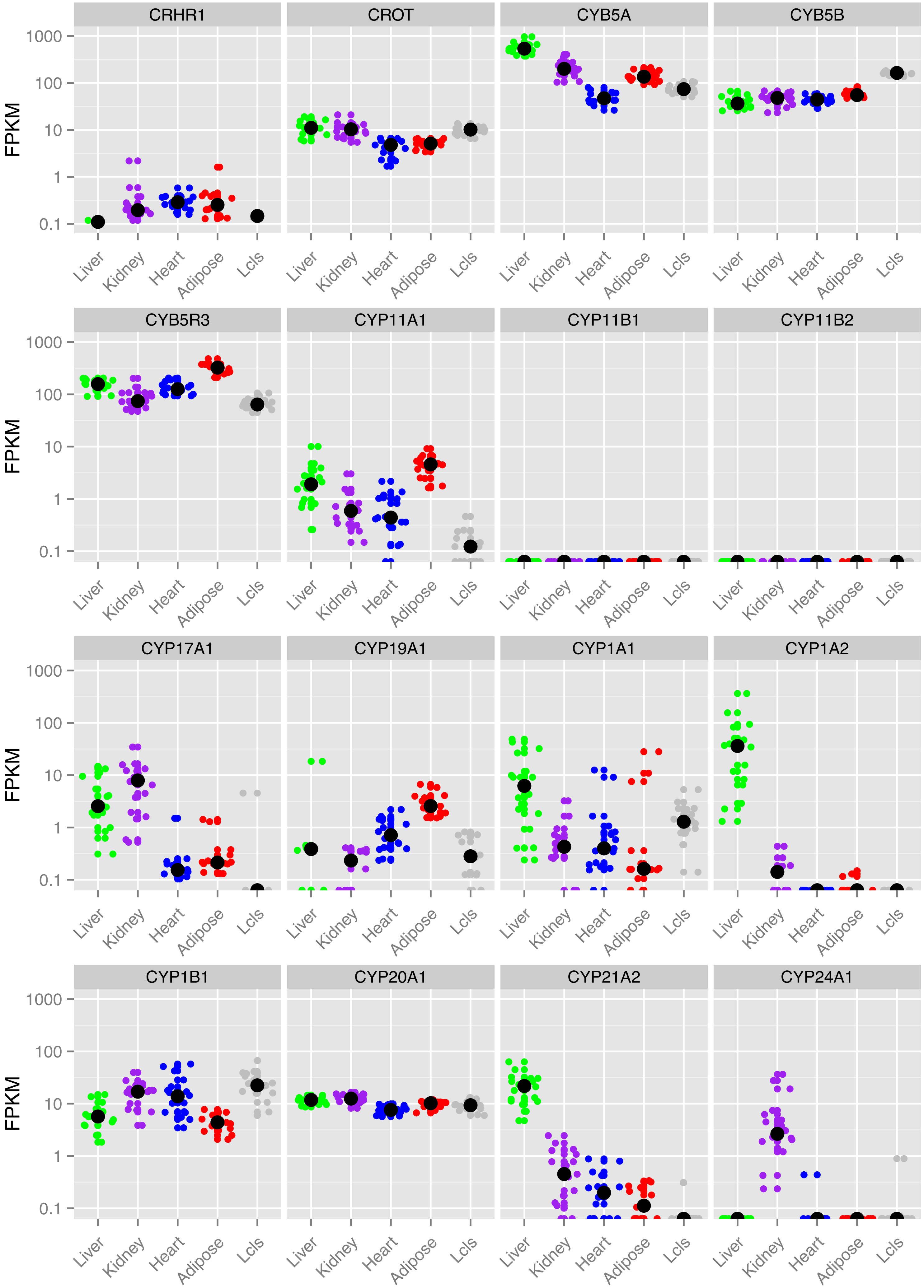

CRHR1
CROT
CYB5A
CYB5B
CYB5R3
CYP11A1
CYP11B1
CYP11B2
CYP17A1
CYP19A1
CYP1A1
CYP1A2
CYP1B1
CYP20A1
CYP21A2
CYP24A1
FPKM
1000
100
10
1
0.1
Liver
Kidney
Heart
Adipose
Lcls

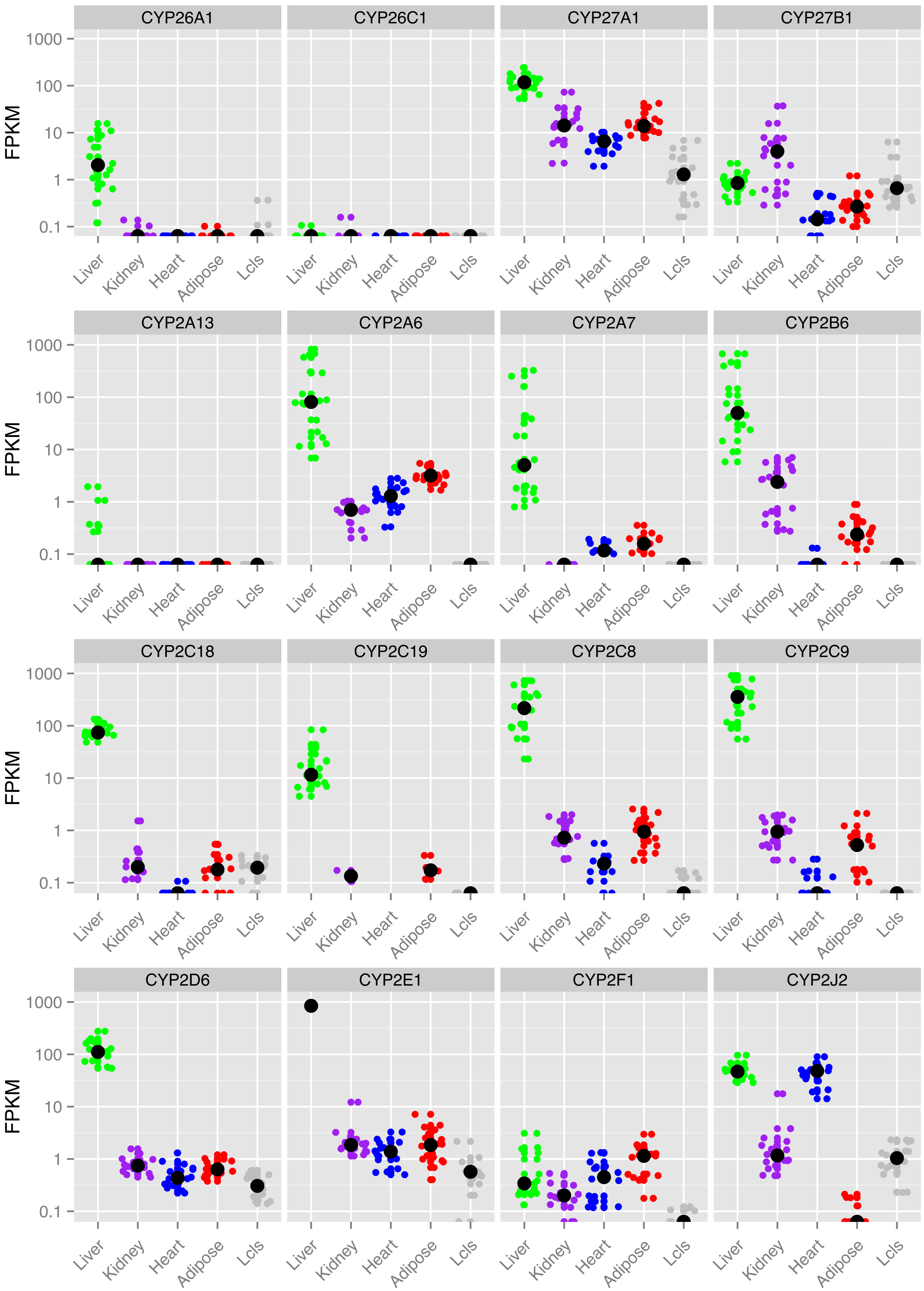
CYP26A1
CYP26C1
CYP27A1
CYP27B1
CYP2A13
CYP2A6
CYP2A7
CYP2B6
CYP2C18
CYP2C19
CYP2C8
CYP2C9
CYP2D6
CYP2E1
CYP2F1
CYP2J2
FPKM
1000
100
10
1
0.1
Liver
Kidney
Heart
Adipose
Lcls

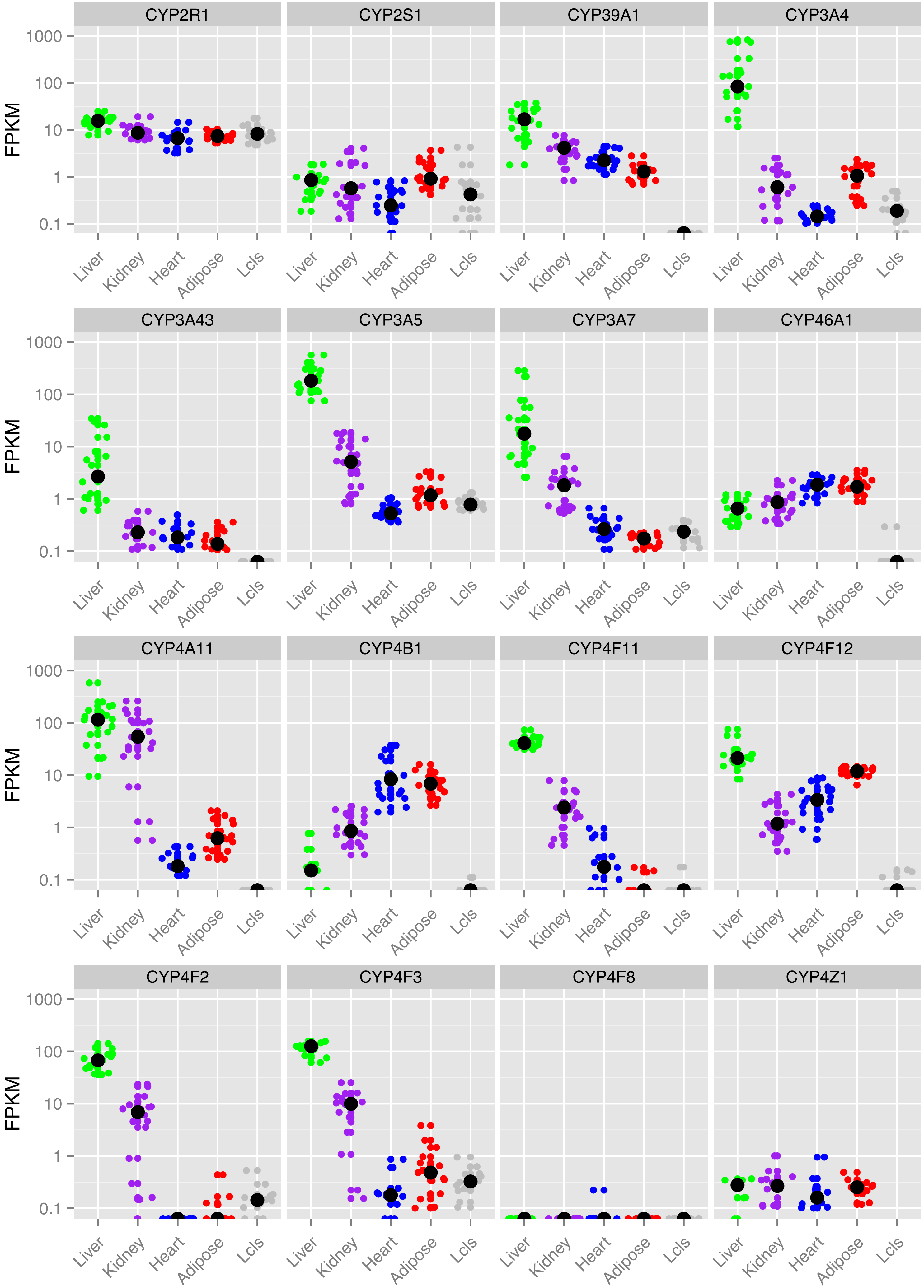
CYP2R1
CYP2S1
CYP39A1
CYP3A4
CYP3A43
CYP3A5
CYP3A7
CYP46A1
CYP4A11
CYP4B1
CYP4F11
CYP4F12
CYP4F2
CYP4F3
CYP4F8
CYP4Z1
FPKM
1000
100
10
1
0.1
Liver
Kidney
Heart
Adipose
Lcls

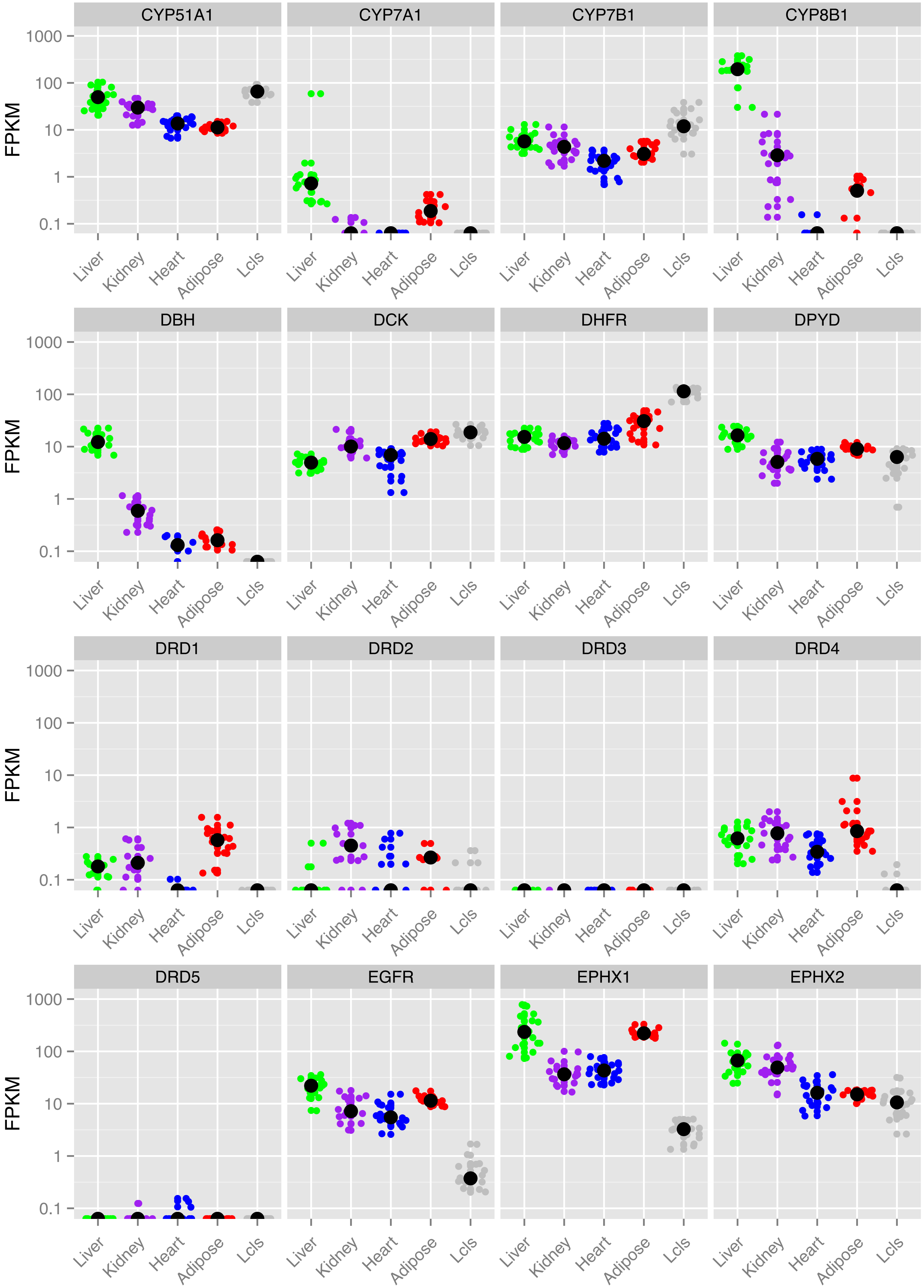

CYP51A1
CYP7A1
CYP7B1
CYP8B1
DBH
DCK
DHFR
DPYD
DRD1
DRD2
DRD3
DRD4
DRD5
EGFR
EPHX1
EPHX2
FPKM
1000
100
10
1
0.1
Liver
Kidney
Heart
Adipose
Lcls

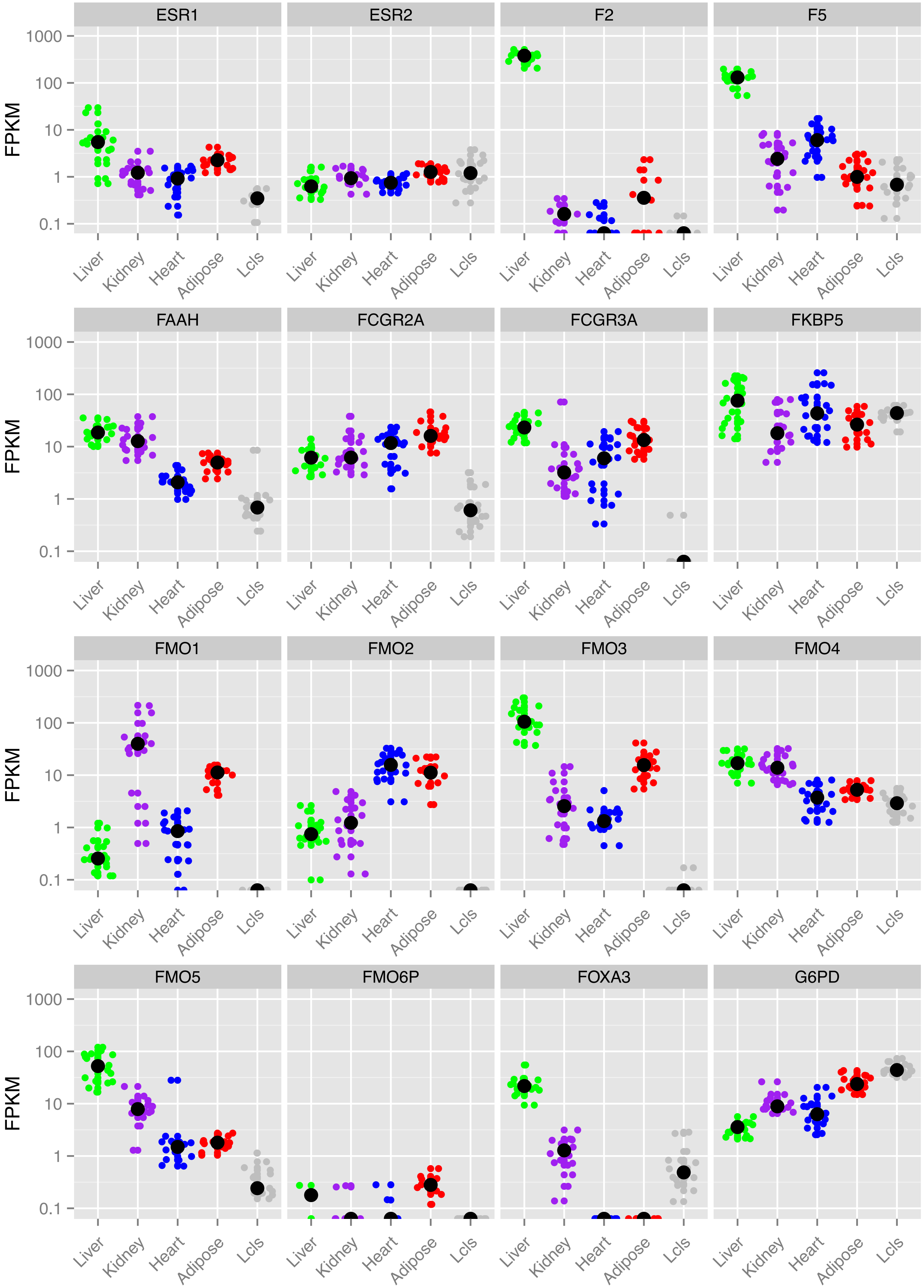
ESR1
ESR2
F2
F5
FAAH
FCGR2A
FCGR3A
FKBP5
FMO1
FMO2
FMO3
FMO4
FMO5
FMO6P
FOXA3
G6PD
FPKM
1000
100
10
1
0.1
Liver
Kidney
Heart
Adipose
Lcls

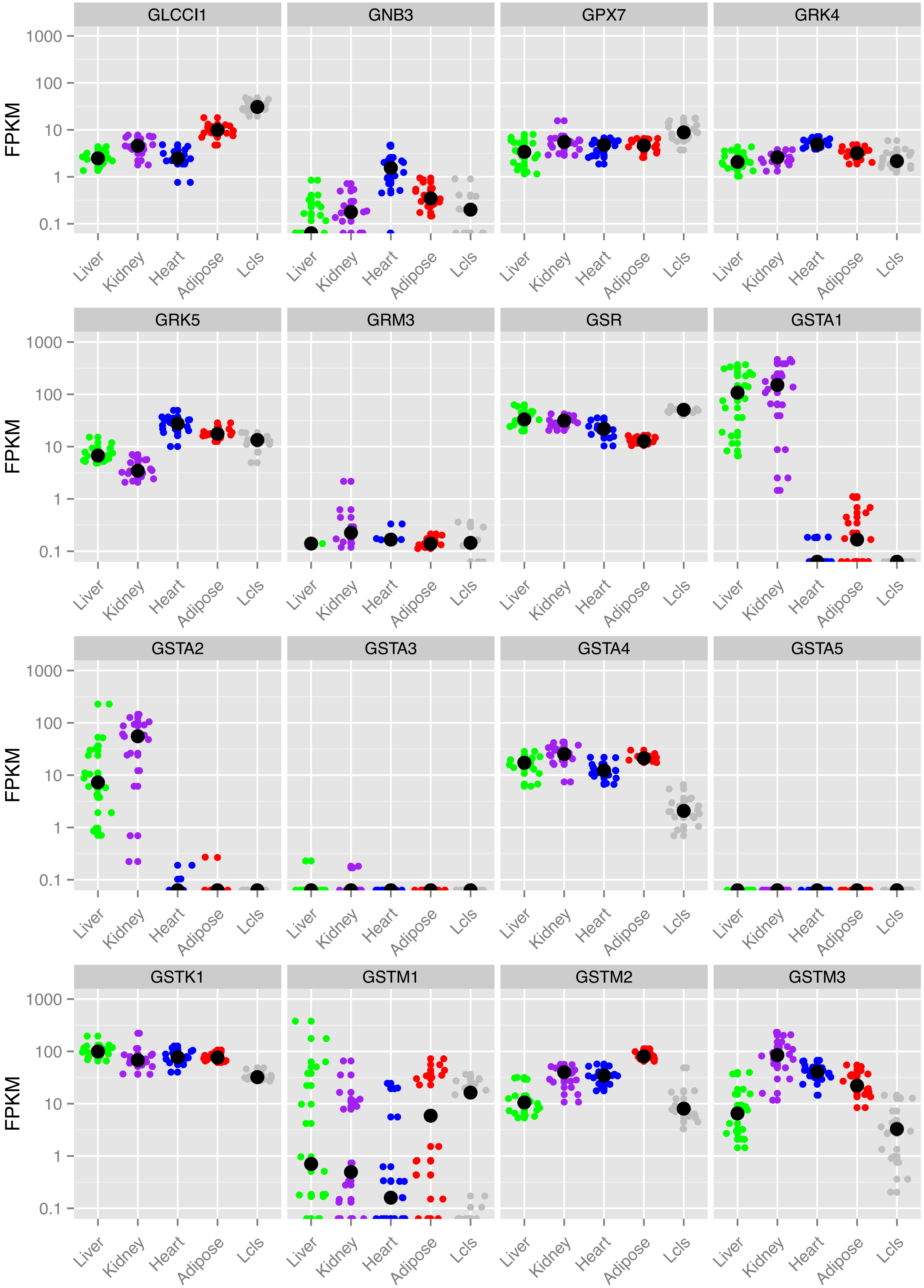
GLCCI1
GNB3
GPX7
GRK4
GRK5
GRM3
GSR
GSTA1
GSTA2
GSTA3
GSTA4
GSTA5
GSTK1
GSTM1
GSTM2
GSTM3
FPKM
1000
100
10
1
0.1
Liver
Kidney
Heart
Adipose
Lcls

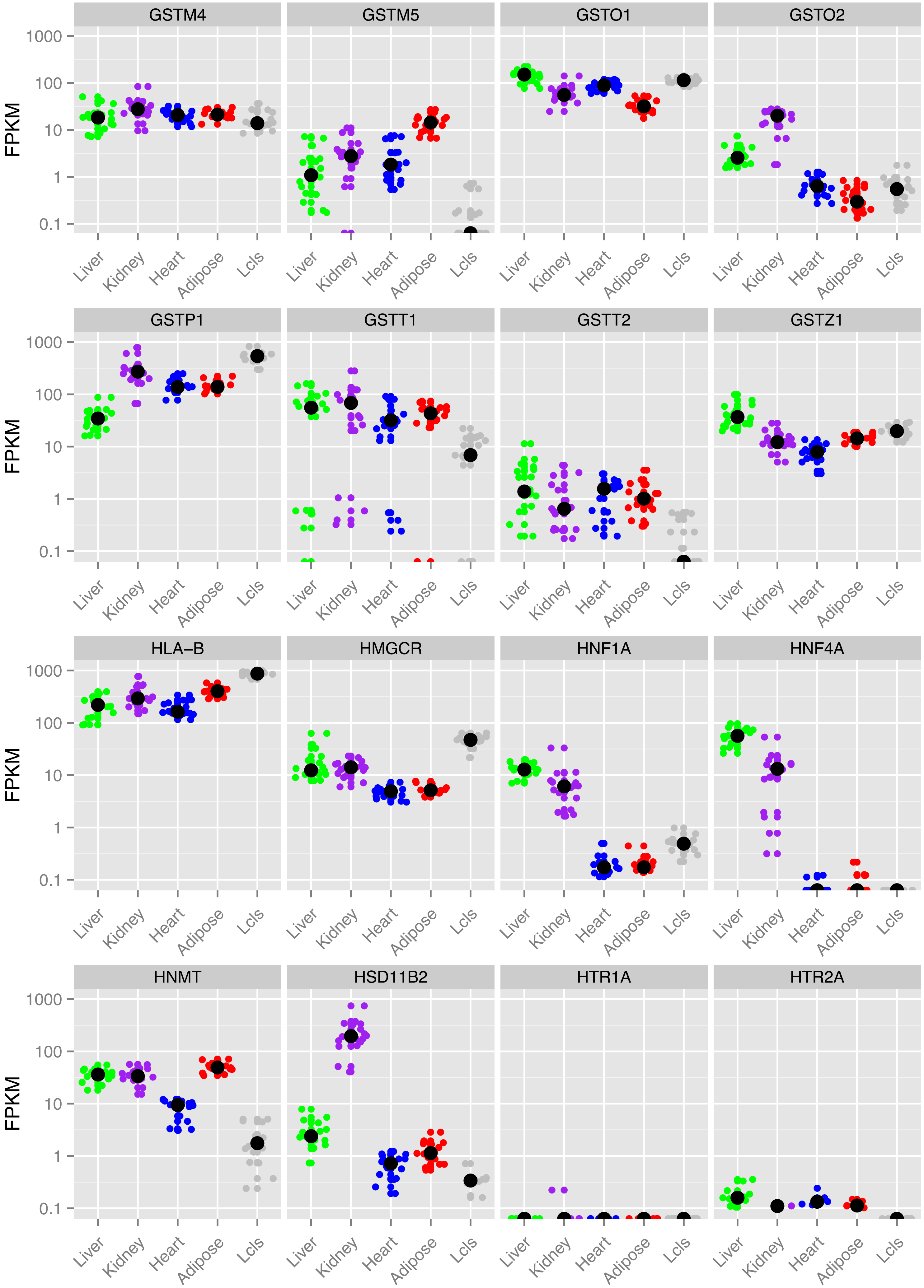

GSTM4
GSTM5
GSTO1
GSTO2
GSTP1
GSTT1
GSTT2
GSTZ1
HLA–B
HMGCR
HNF1A
HNF4A
HNMT
HSD11B2
HTR1A
HTR2A
FPKM
1000
100
10
1
0.1
Liver
Kidney
Heart
Adipose
Lcls

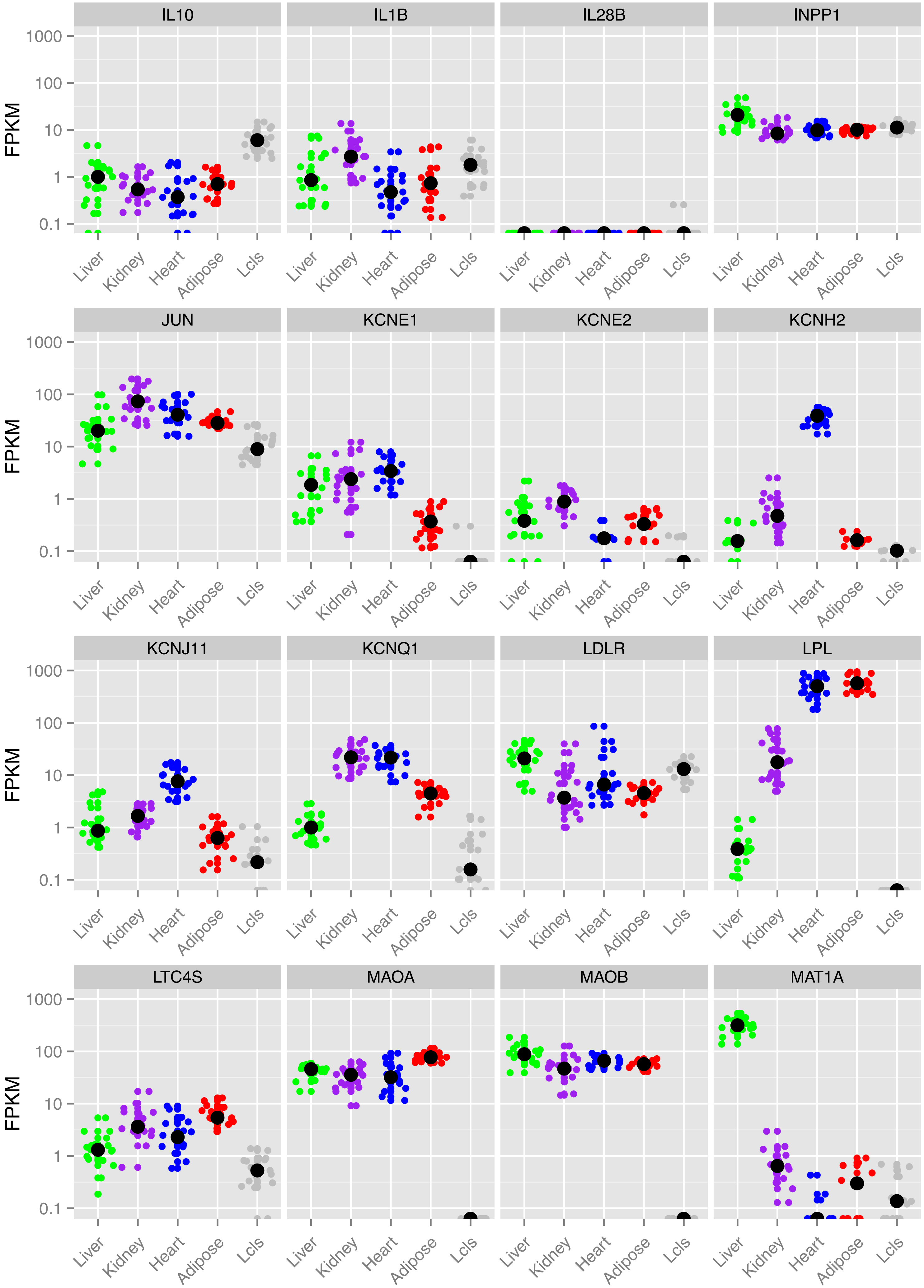
IL10
IL1B
IL28B
INPP1
JUN
KCNE1
KCNE2
KCNH2
KCNJ11
KCNQ1
LDLR
LPL
LTC4S
MAOA
MAOB
MAT1A
FPKM
1000
100
10
1
0.1
Liver
Kidney
Heart
Adipose
Lcls

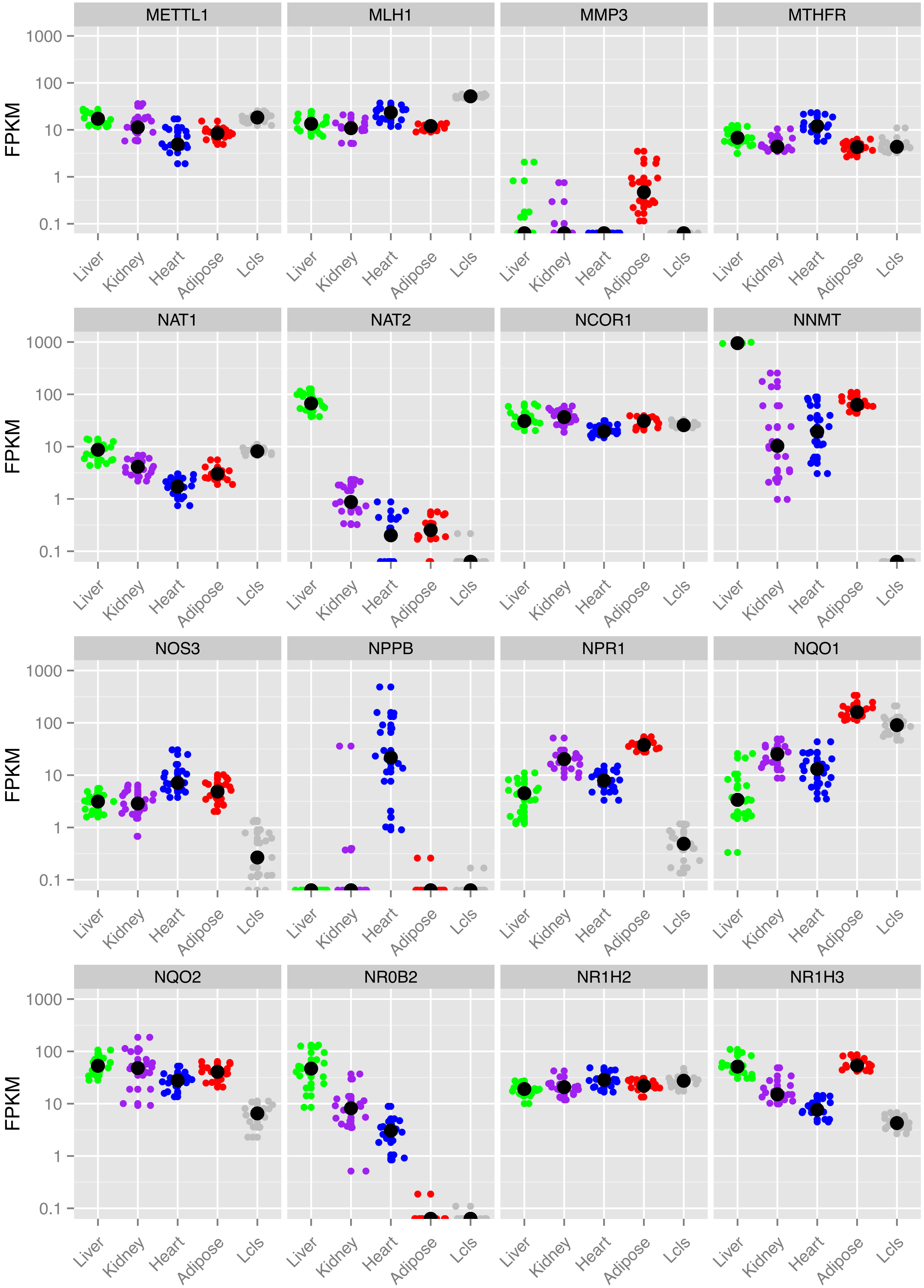
METTL1
MLH1
MMP3
MTHFR
NAT1
NAT2
NCOR1
NNMT
NOS3
NPPB
NPR1
NQO1
NQO2
NR0B2
NR1H2
NR1H3
FPKM
1000
100
10
1
0.1
Liver
Kidney
Heart
Adipose
Lcls

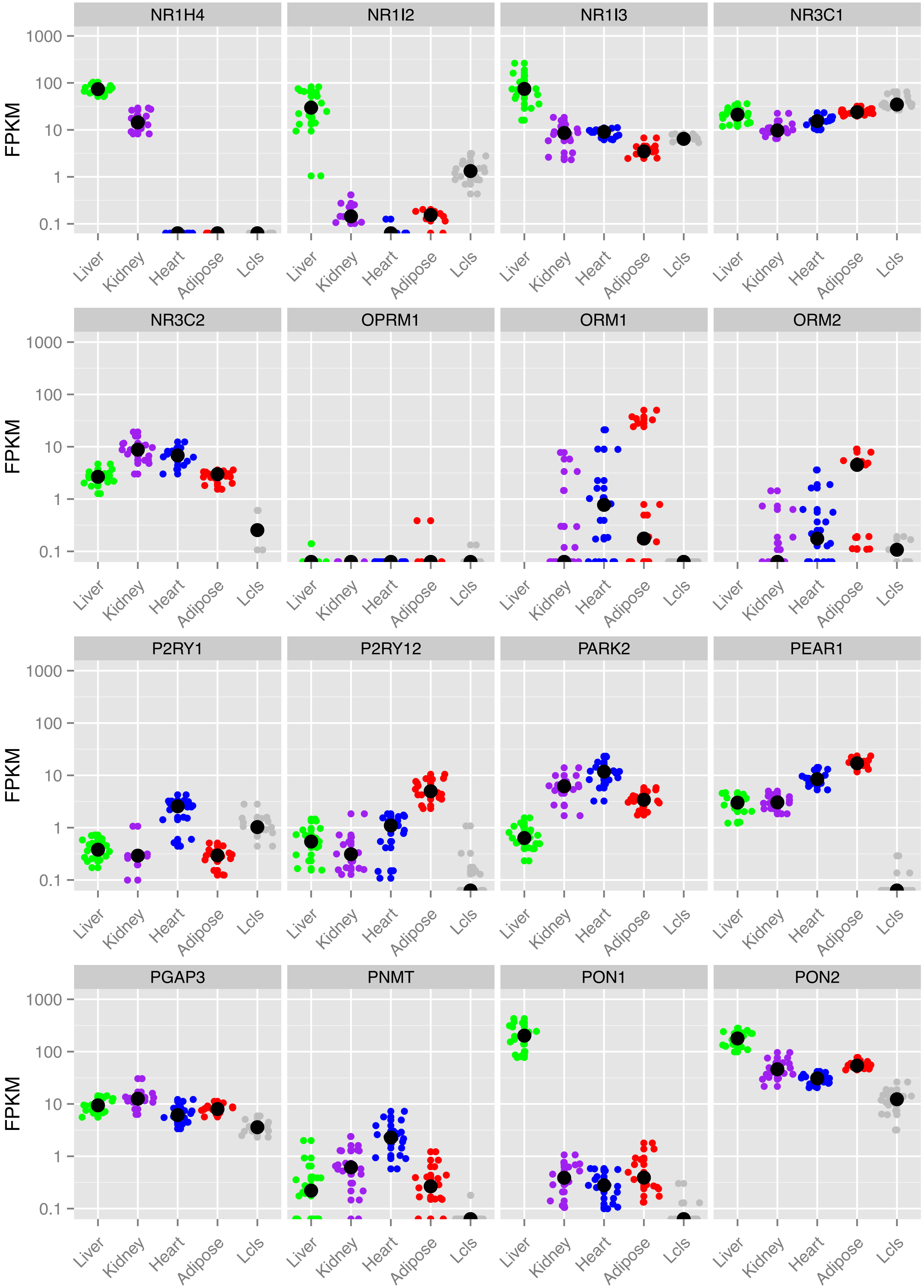
NR1H4
NR1I2
NR1I3
NR3C1
NR3C2
OPRM1
ORM1
ORM2
P2RY1
P2RY12
PARK2
PEAR1
PGAP3
PNMT
PON1
PON2
FPKM
1000
100
10
1
0.1
Liver
Kidney
Heart
Adipose
Lcls

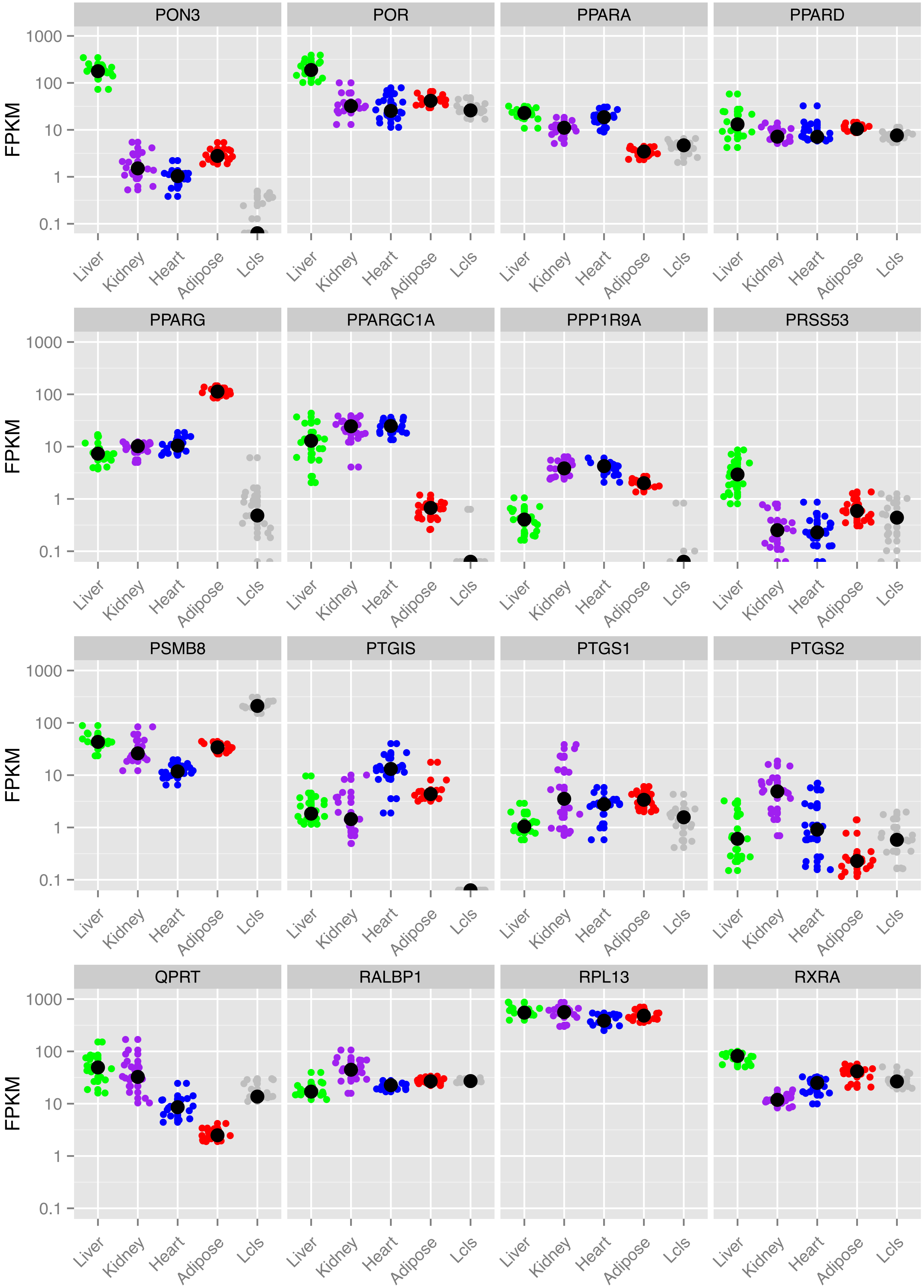
PON3
POR
PPARA
PPARD
PPARG
PPARGC1A
PPP1R9A
PRSS53
PSMB8
PTGIS
PTGS1
PTGS2
QPRT
RALBP1
RPL13
RXRA
FPKM
1000
100
10
1
0.1
Liver
Kidney
Heart
Adipose
Lcls

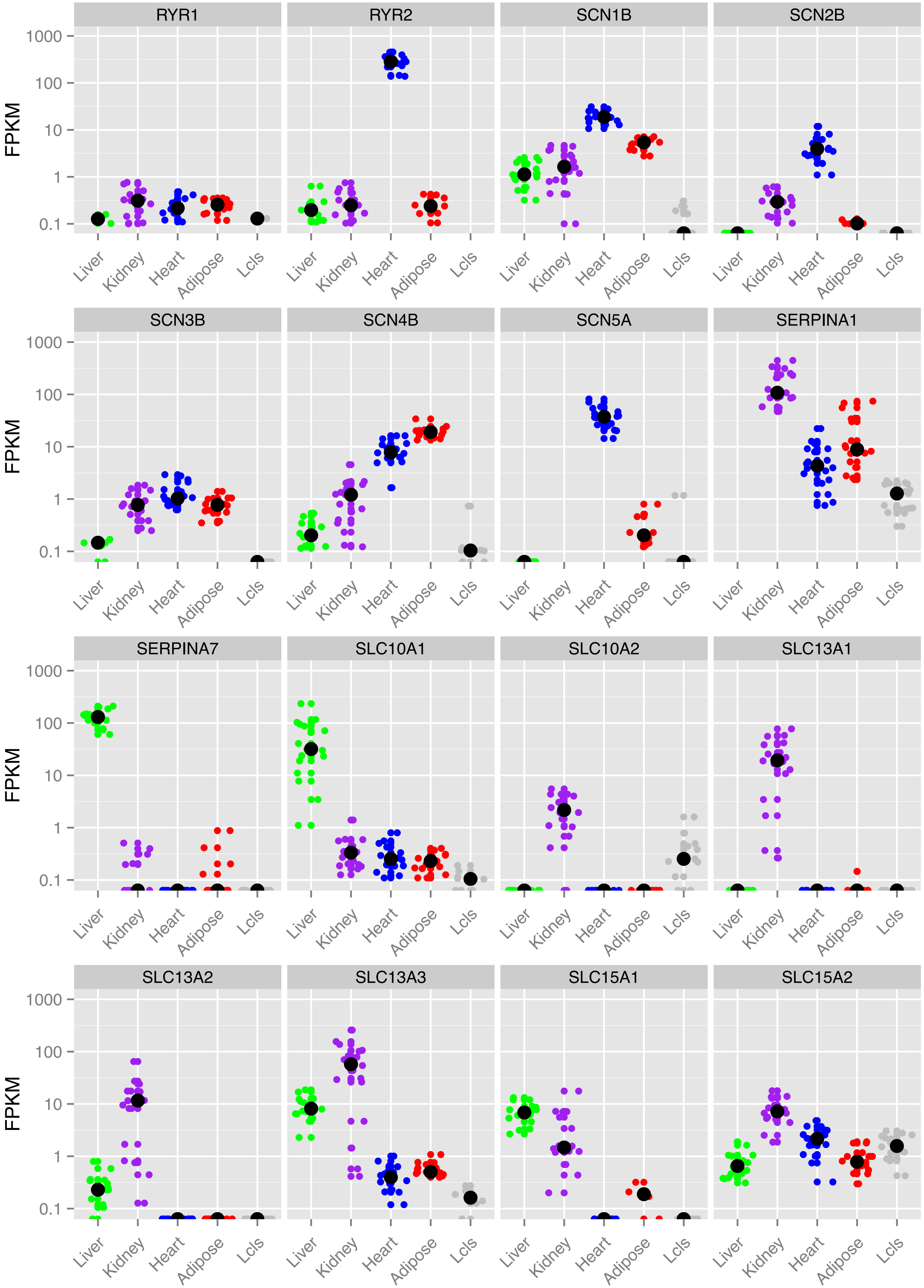

RYR1
RYR2
SCN1B
SCN2B
SCN3B
SCN4B
SCN5A
SERPINA1
SERPINA7
SLC10A1
SLC10A2
SLC13A1
SLC13A2
SLC13A3
SLC15A1
SLC15A2
FPKM
1000
100
10
1
0.1
Liver
Kidney
Heart
Adipose
Lcls

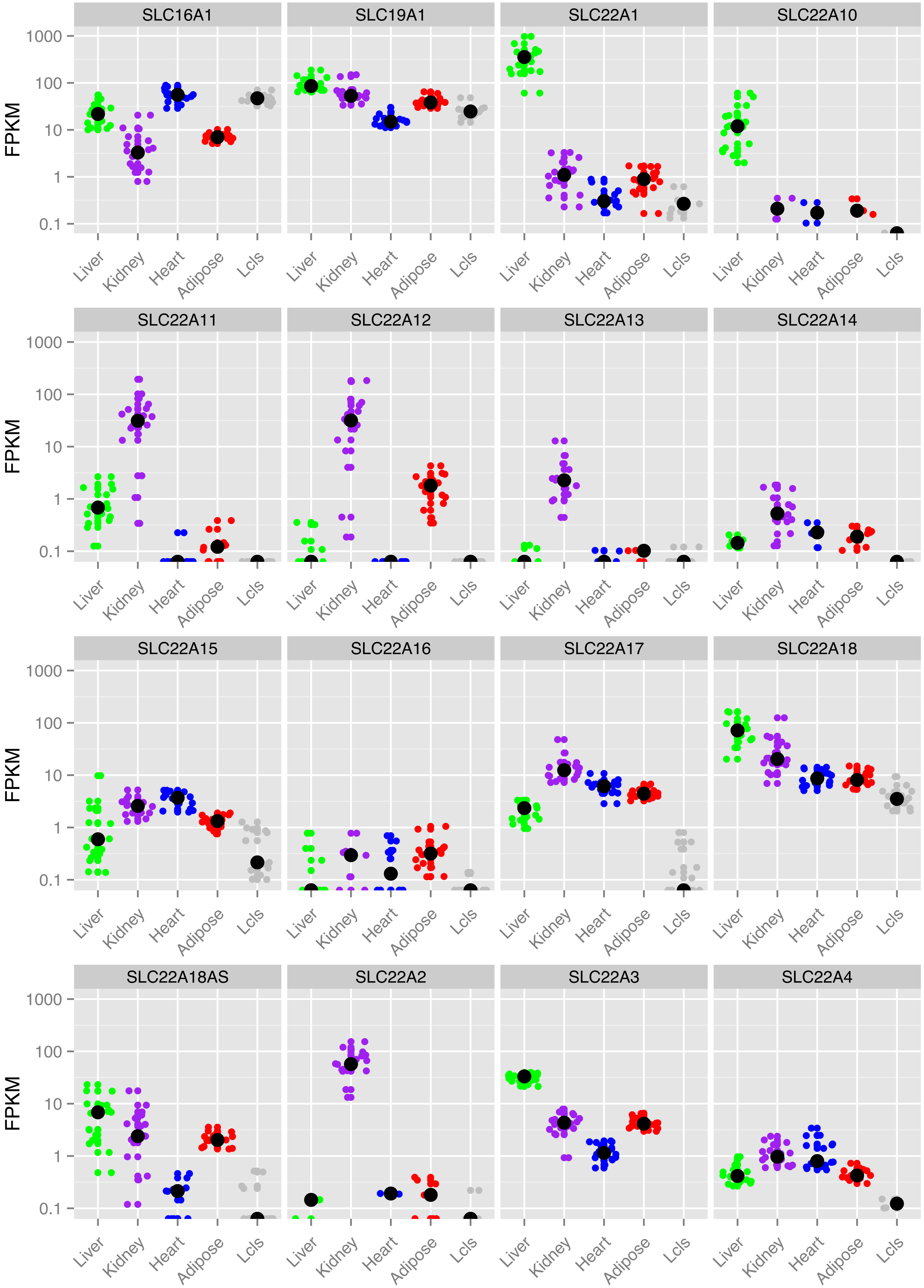

SLC16A1
SLC19A1
SLC22A1
SLC22A10
SLC22A11
SLC22A12
SLC22A13
SLC22A14
SLC22A15
SLC22A16
SLC22A17
SLC22A18
SLC22A18AS
SLC22A2
SLC22A3
SLC22A4
FPKM
1000
100
10
1
0.1
Liver
Kidney
Heart
Adipose
Lcls

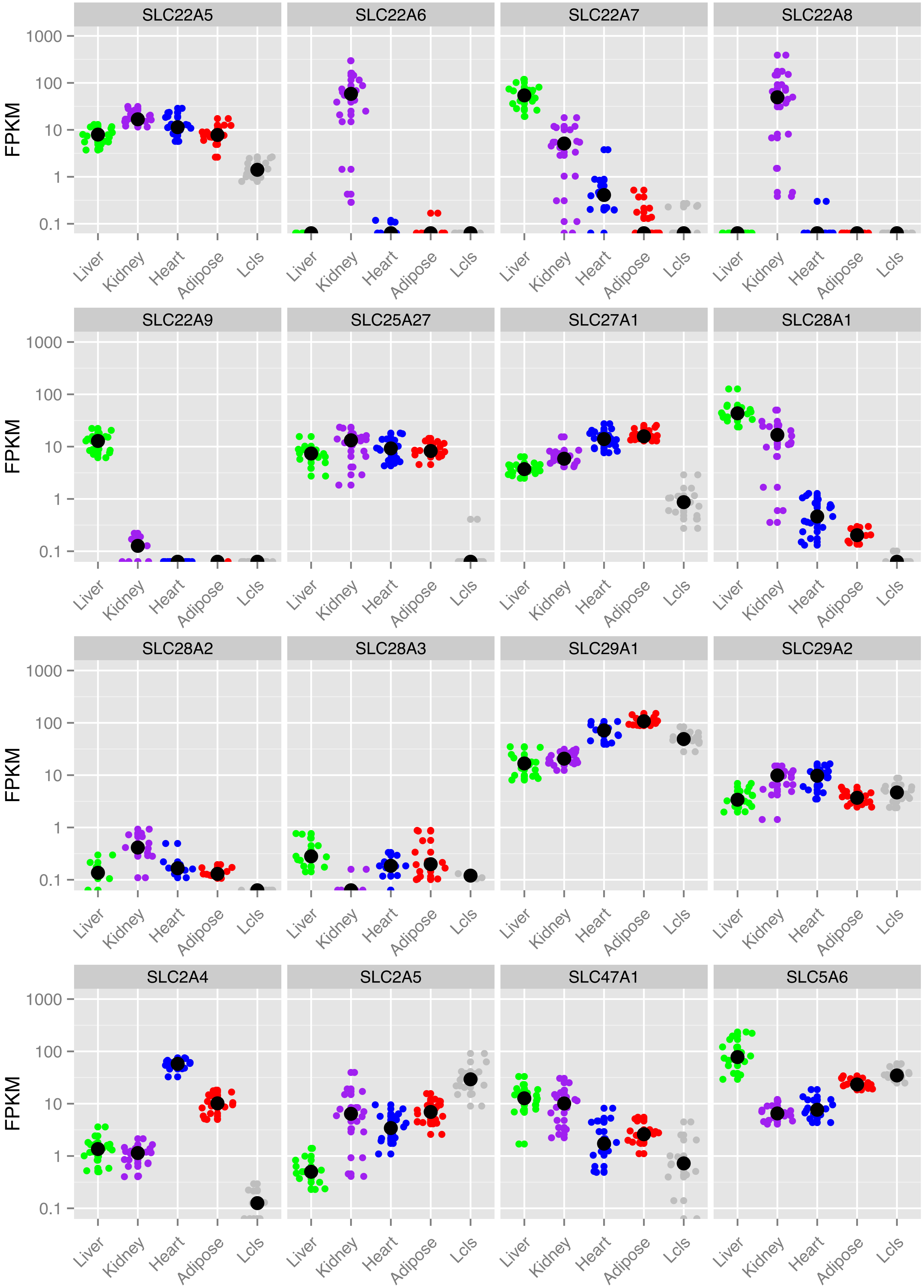
SLC22A5
SLC22A6
SLC22A7
SLC22A8
SLC22A9
SLC25A27
SLC27A1
SLC28A1
SLC28A2
SLC28A3
SLC29A1
SLC29A2
SLC2A4
SLC2A5
SLC47A1
SLC5A6
FPKM
1000
100
10
1
0.1
Liver
Kidney
Heart
Adipose
Lcls

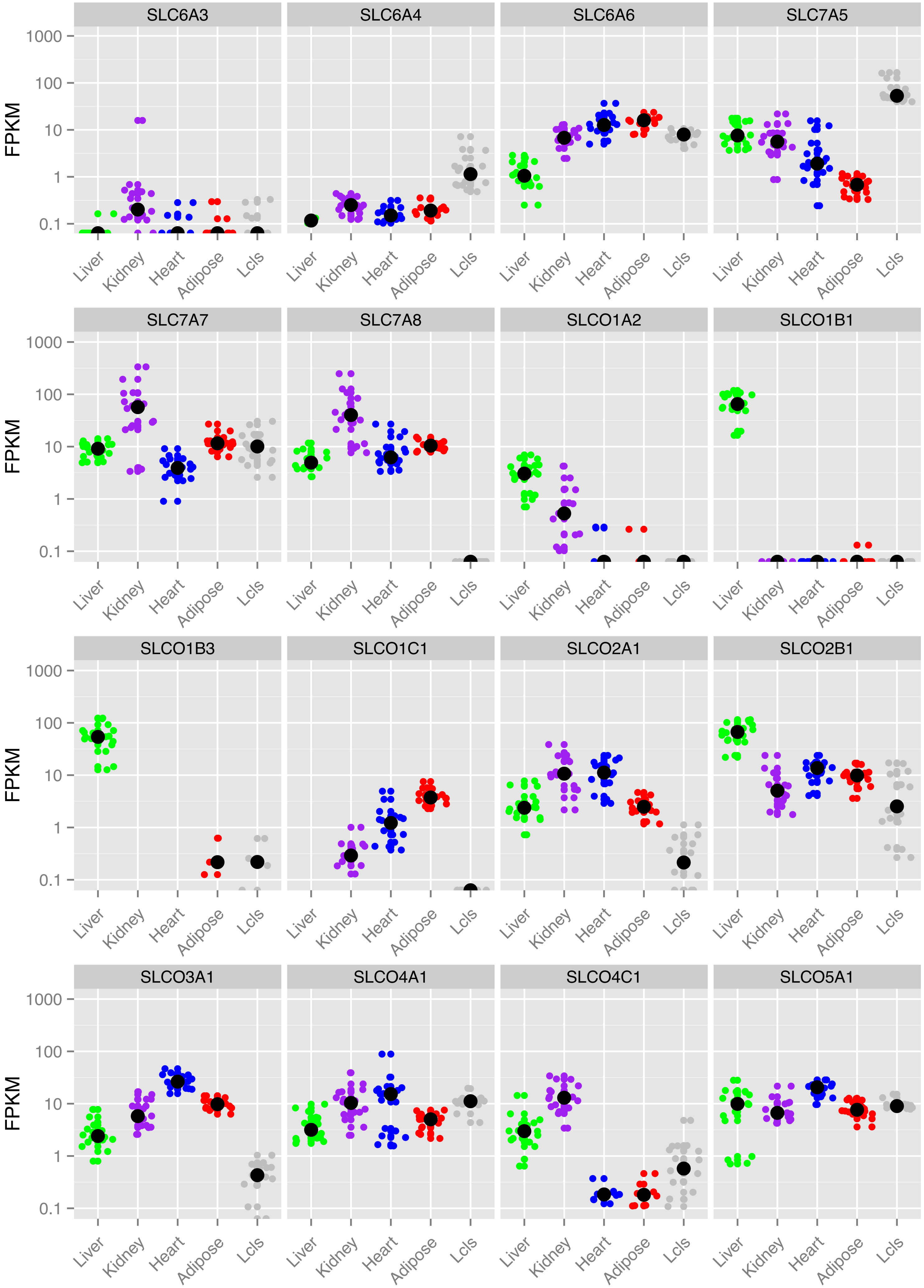
SLC6A3
SLC6A4
SLC6A6
SLC7A5
SLC7A7
SLC7A8
SLCO1A2
SLCO1B1
SLCO1B3
SLCO1C1
SLCO2A1
SLCO2B1
SLCO3A1
SLCO4A1
SLCO4C1
SLCO5A1
FPKM
1000
100
10
1
0.1
Liver
Kidney
Heart
Adipose
Lcls

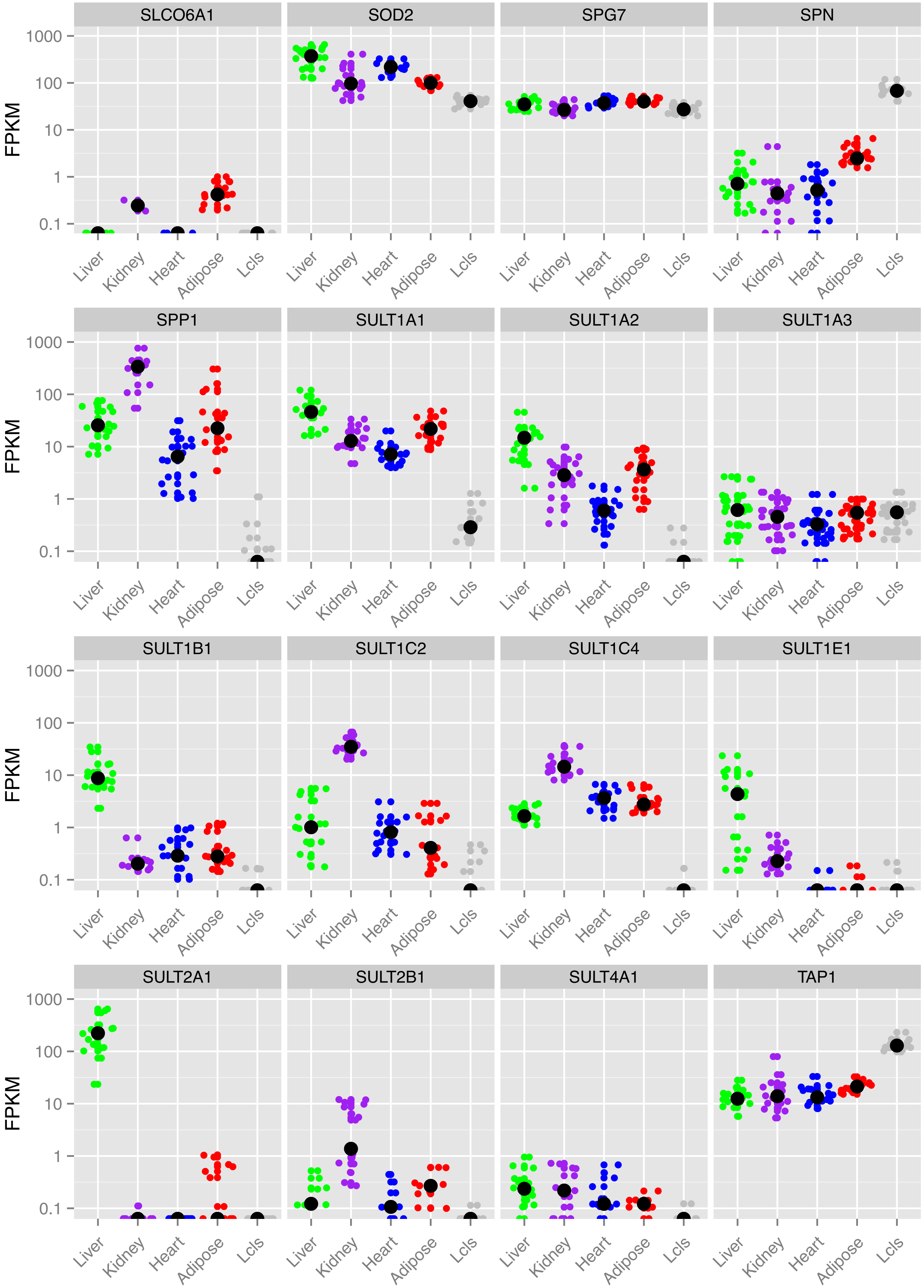
SLCO6A1
SOD2
SPG7
SPN
SPP1
SULT1A1
SULT1A2
SULT1A3
SULT1B1
SULT1C2
SULT1C4
SULT1E1
SULT2A1
SULT2B1
SULT4A1
TAP1
FPKM
1000
100
10
1
0.1
Liver
Kidney
Heart
Adipose
Lcls

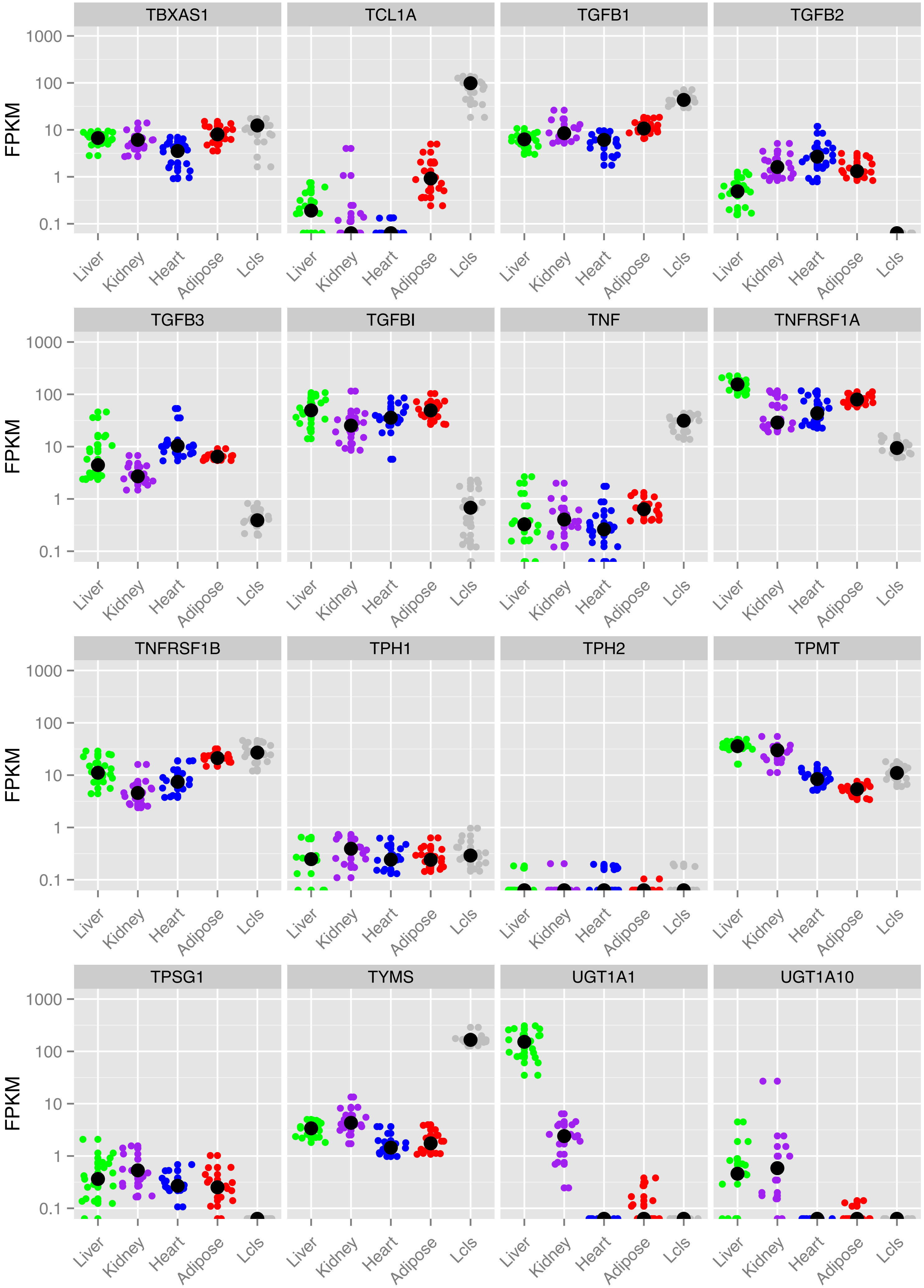

TBXAS1
TCL1A
TGFB1
TGFB2
TGFB3
TGFBI
TNF
TNFRSF1A
TNFRSF1B
TPH1
TPH2
TPMT
TPSG1
TYMS
UGT1A1
UGT1A10
FPKM
1000
100
10
1
0.1
Liver
Kidney
Heart
Adipose
Lcls

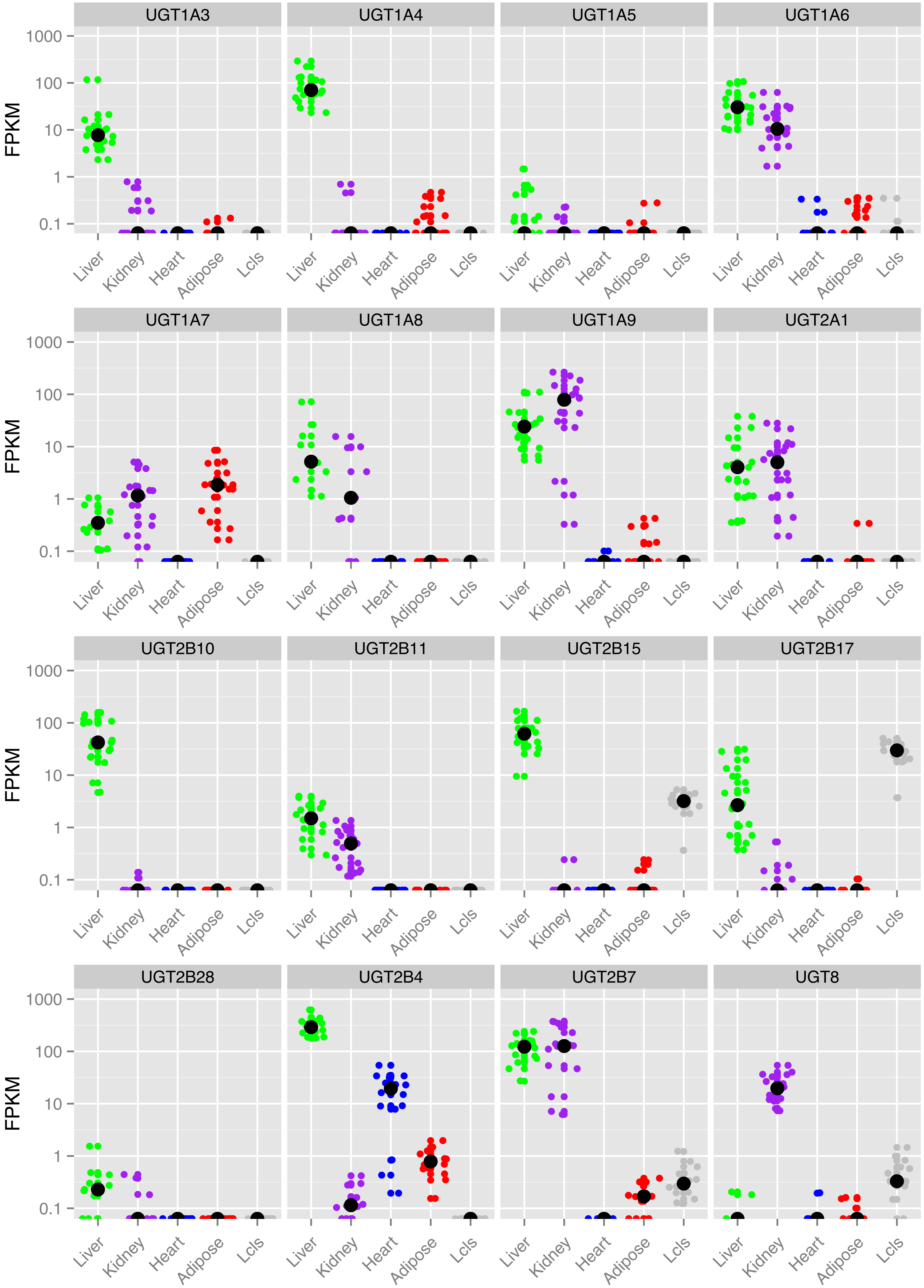

UGT1A3
UGT1A4
UGT1A5
UGT1A6
UGT1A7
UGT1A8
UGT1A9
UGT2A1
UGT2B10
UGT2B11
UGT2B15
UGT2B17
UGT2B28
UGT2B4
UGT2B7
UGT8
FPKM
1000
100
10
1
0.1
Liver
Kidney
Heart
Adipose
Lcls

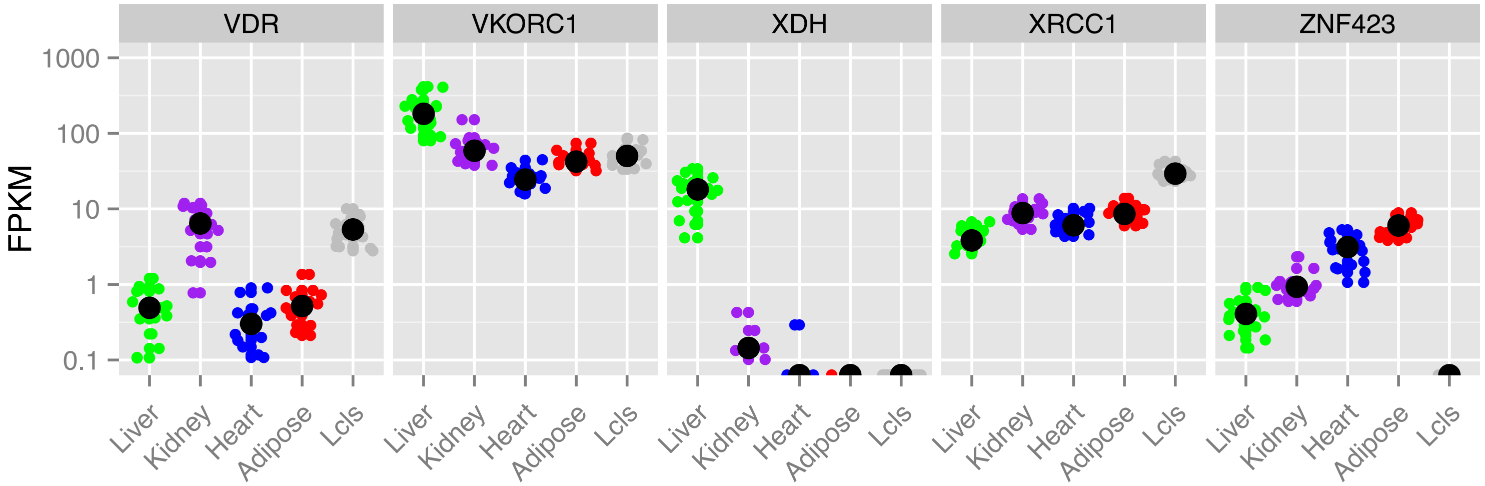

VDR
VKORC1
XDH
XRCC1
ZNF423
FPKM
1000
100
10
1
0.1
Liver
Kidney
Heart
Adipose
Lcls

S4

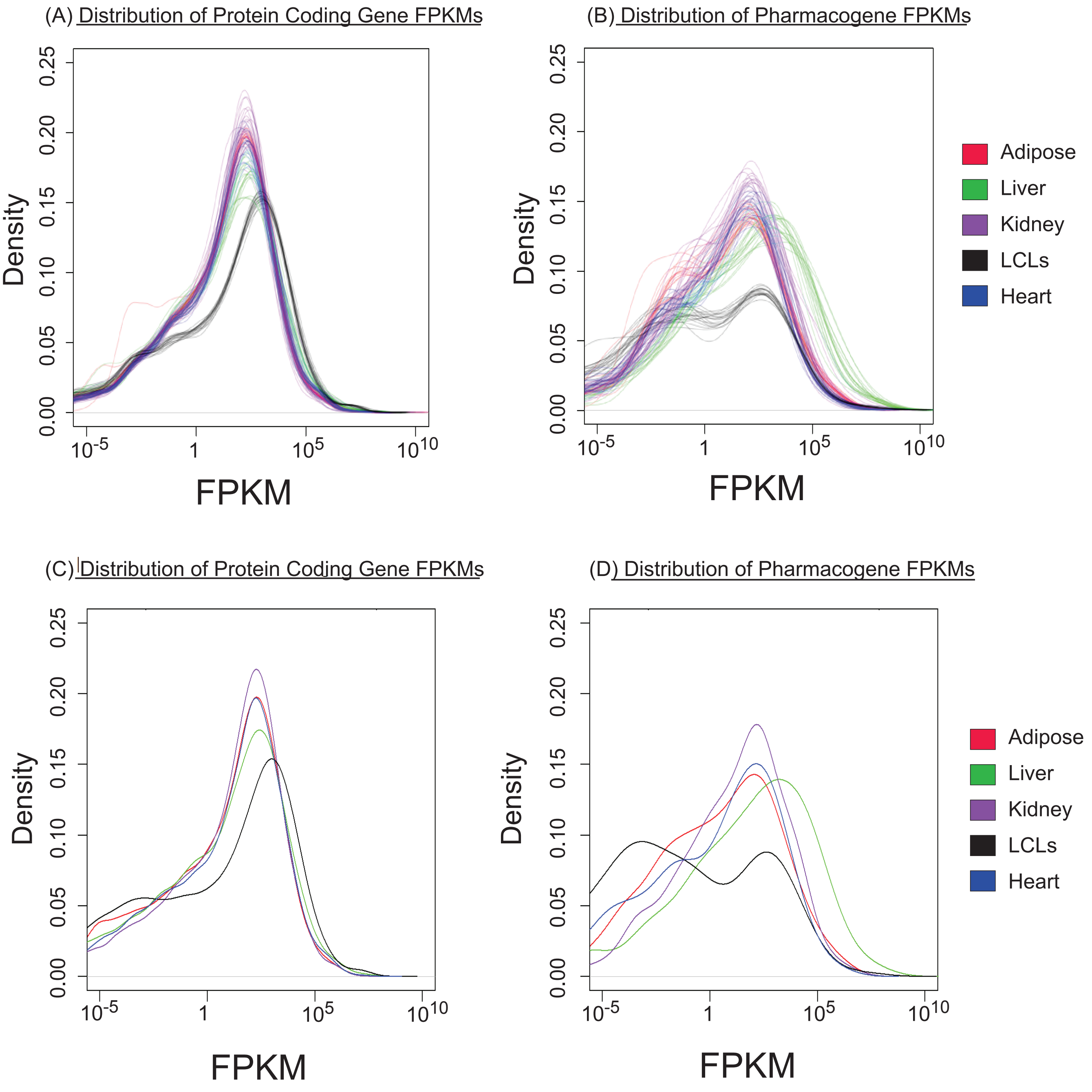
(A) Distribution of Protein Coding Gene FPKMs
(B) Distribution of Pharmacogene FPKMs
(C) Distribution of Protein Coding Gene FPKMs
(D) Distribution of Pharmacogene FPKMs
Density
FPKM
0.00
0.05
0.10
0.15
0.20
0.25
10-5
1
105
1010
Adipose
Liver
Kidney
LCLs
Heart

S5A

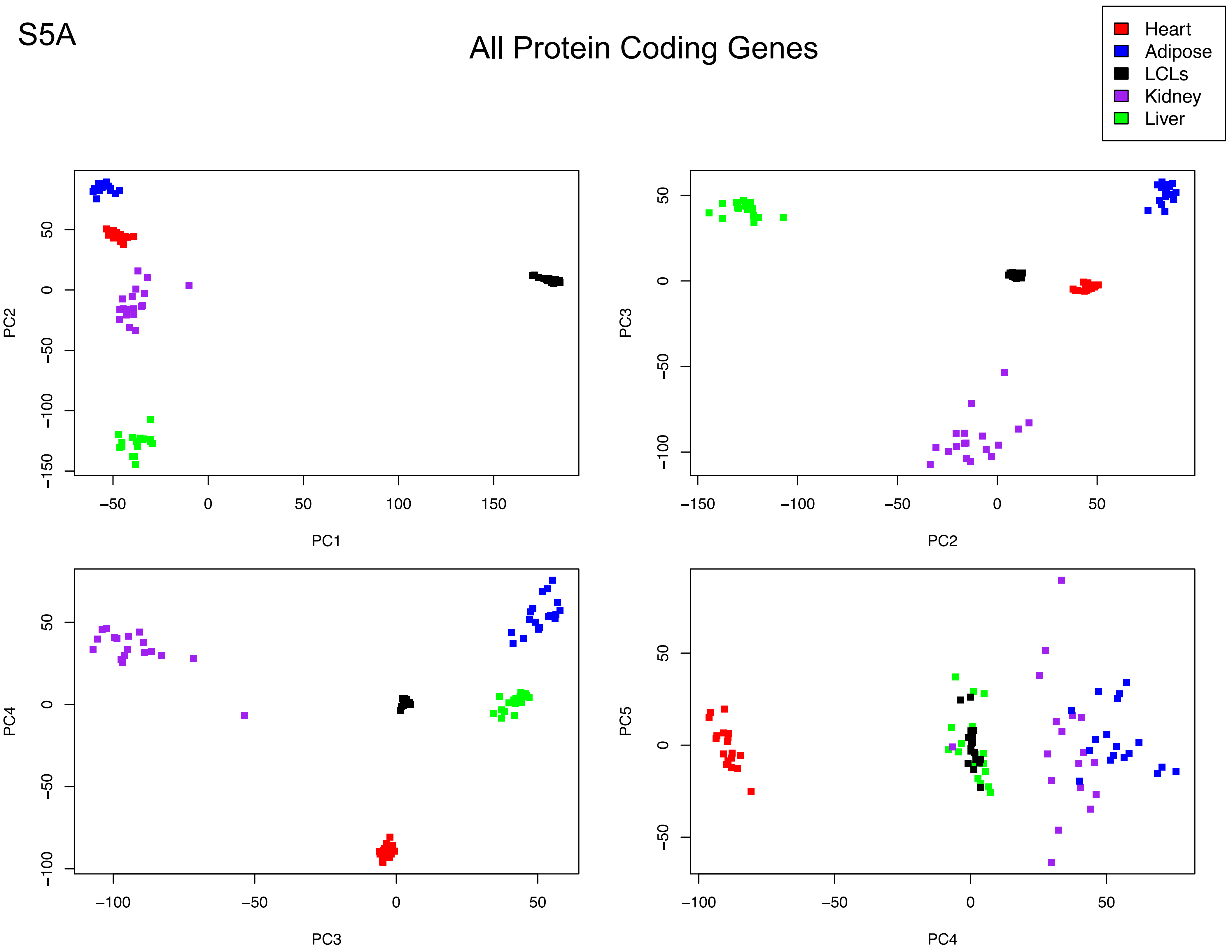
All Protein Coding Genes
Heart
Adipose
LCLs
Kidney
Liver
PC1
PC2
PC2
PC3
PC3
PC4
PC4
PC5
−50
0
50
100
150
−150
−100
−50
0
50

S5B

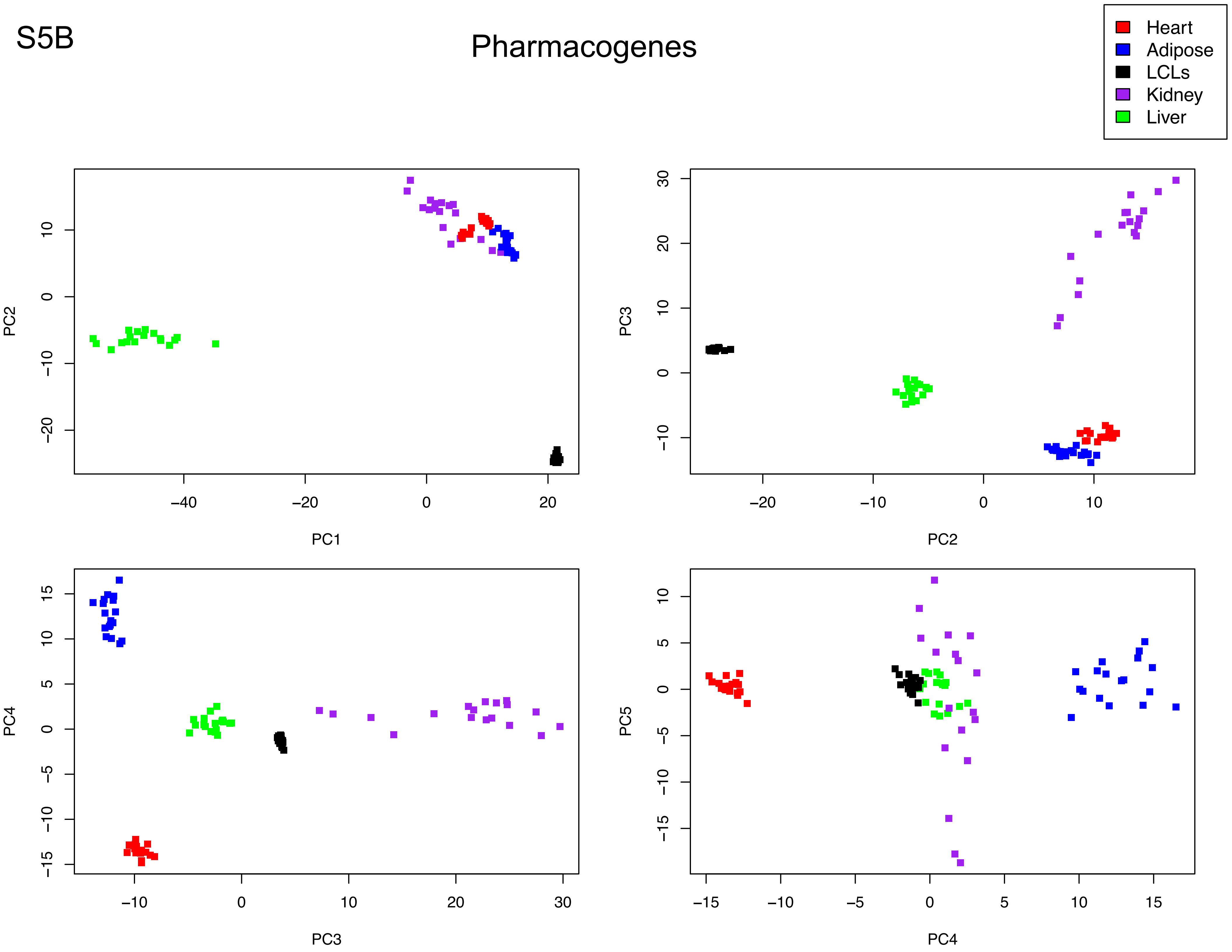
Pharmacogenes
Heart
Adipose
LCLs
Kidney
Liver
PC1
PC2
PC3
PC4
PC5

S6

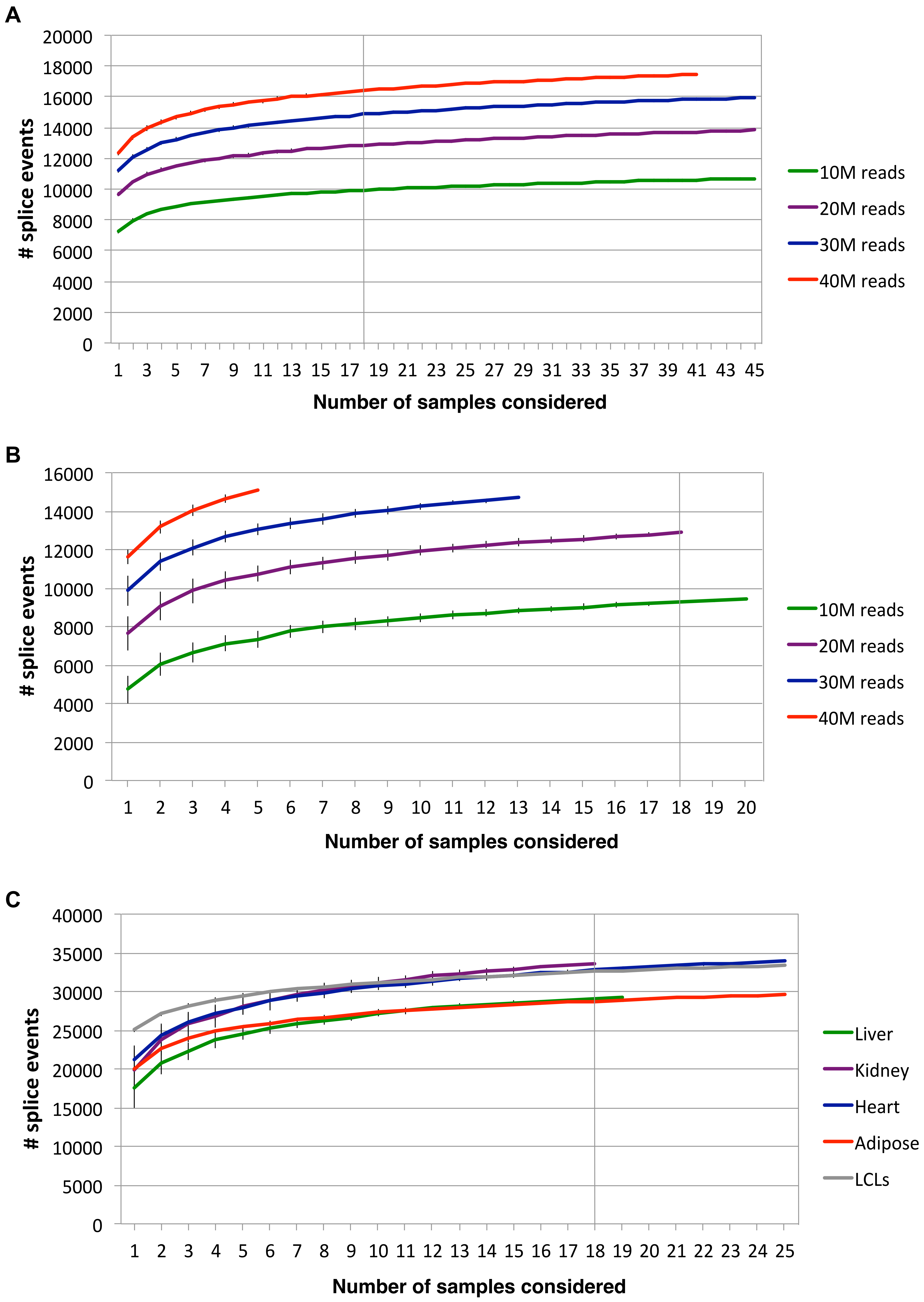
A
20000
18000
16000
14000
12000
10000
8000
6000
4000
2000
0
splice events
1 3 5 7 9 11 13 15 17 19 21 23 25 27 29 31 33 35 37 39 41 43 45
Number of samples considered
10M reads
20M reads
30M reads
40M reads
B
16000
14000
12000
10000
8000
6000
4000
2000
0
splice events
1 2 3 4 5 6 7 8 9 10 11 12 13 14 15 16 17 18 19 20
Number of samples considered
10M reads
20M reads
30M reads
40M reads
C
40000
35000
30000
25000
20000
15000
10000
5000
0
splice events
1 2 3 4 5 6 7 8 9 10 11 12 13 14 15 16 17 18 19 20 21 22 23 24 25
Number of samples considered
Liver
Kidney
Heart
Adipose
LCLs

S7

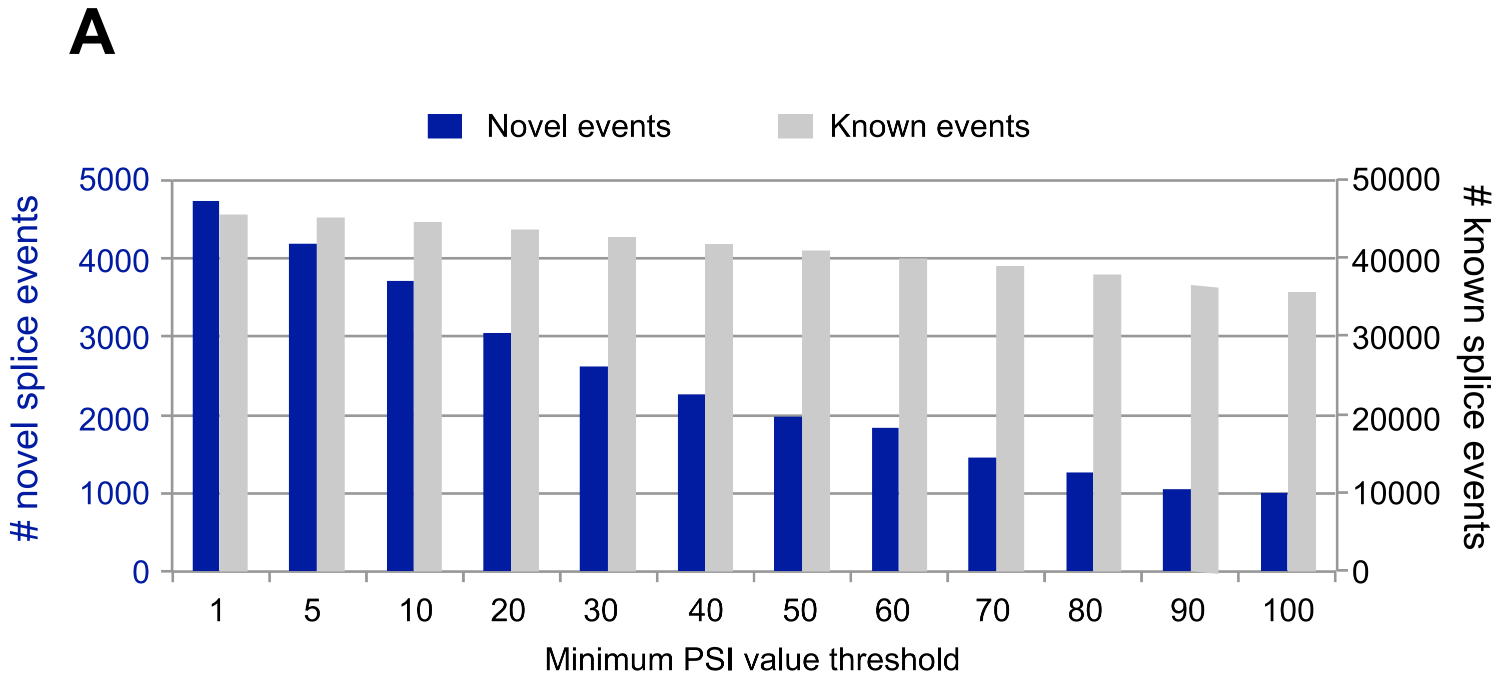
A
Novel events
Known events
5000
4000
3000
2000
1000
0
novel splice events
50000
40000
30000
20000
10000
0
known splice events
1
5
10
20
30
40
50
60
70
80
90
100
Minimum PSI value threshold

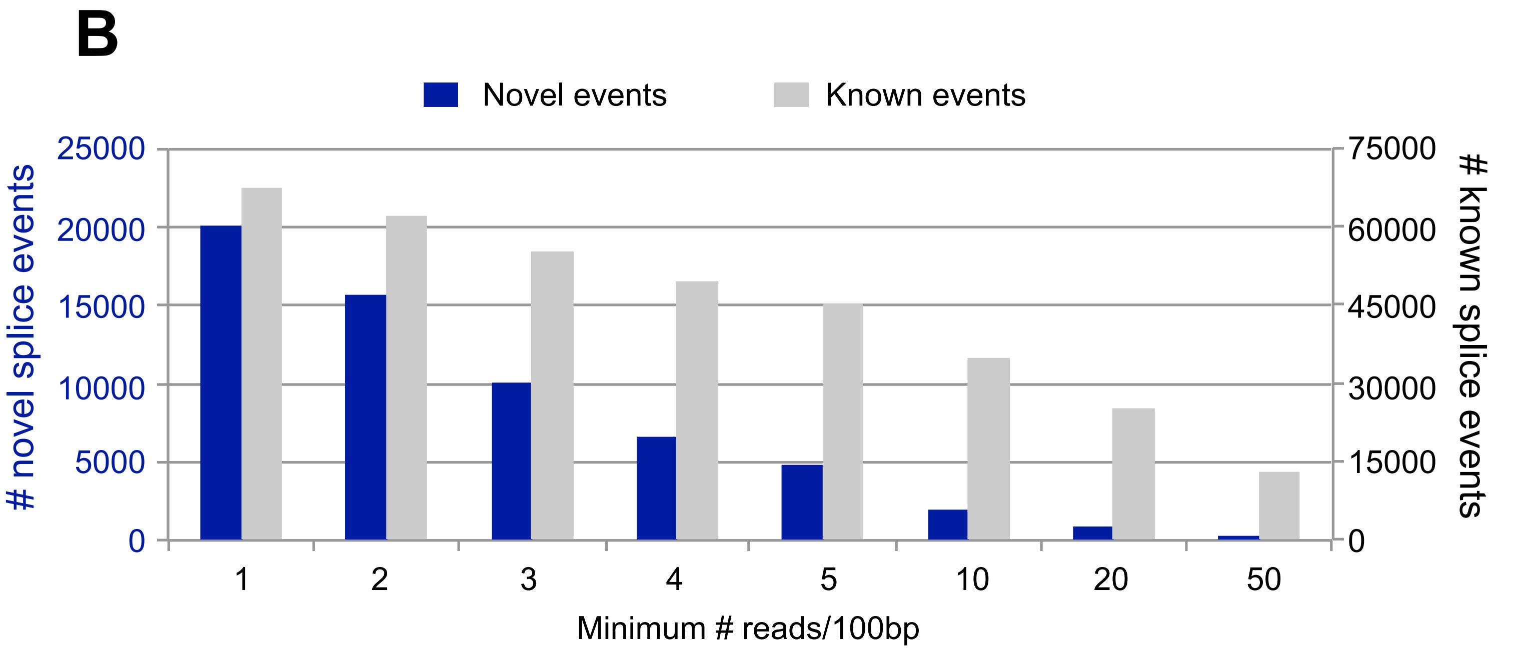
B
Novel events
Known events
25000
20000
15000
10000
5000
0
novel splice events
75000
60000
45000
30000
15000
0
known splice events
1
2
3
4
5
10
20
50
Minimum # reads/100bp

S8

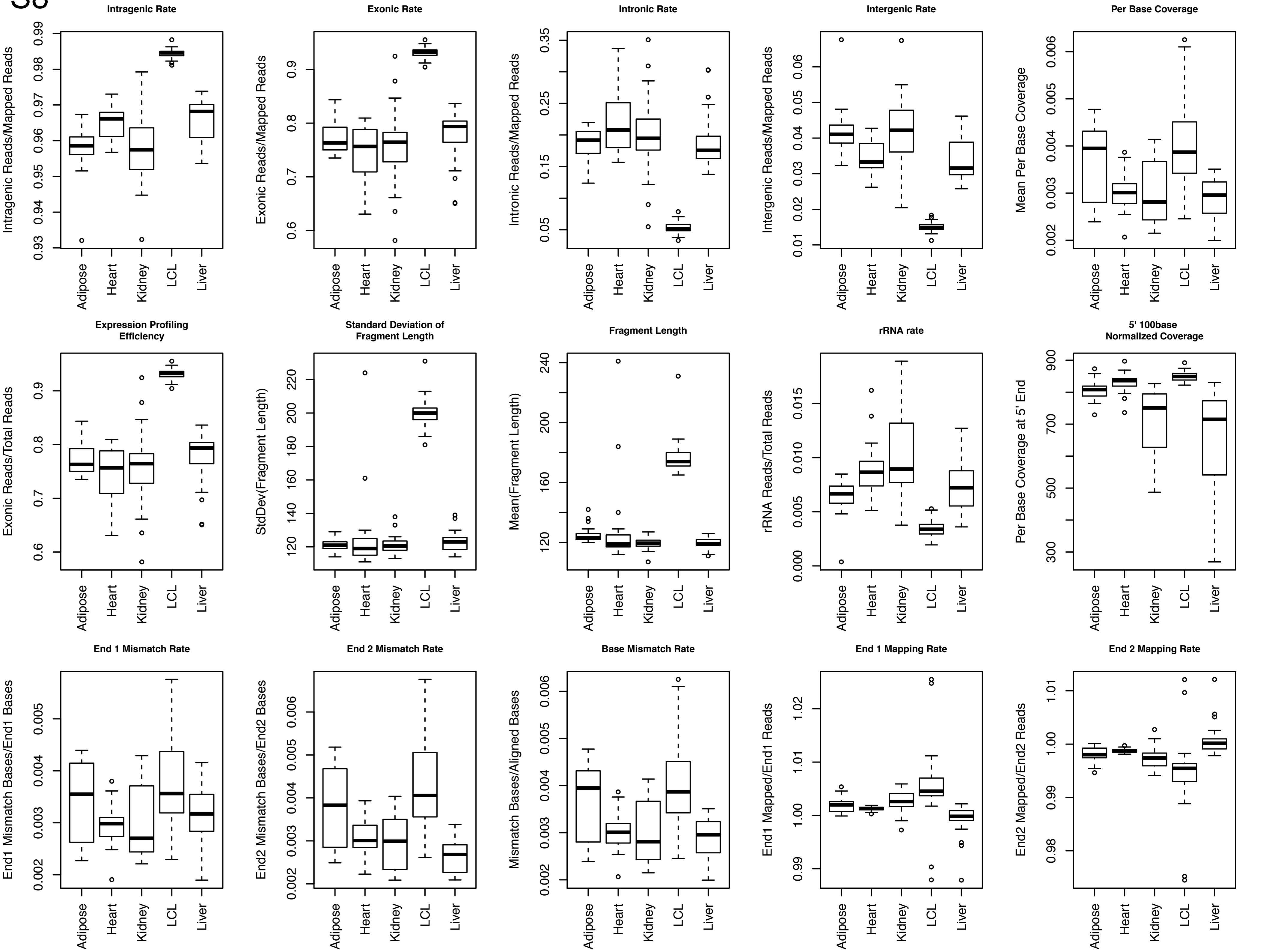

Intragenic Rate
Intragenic Reads/Mapped Reads
0.93 0.94 0.95 0.96 0.97 0.98 0.99
Exonic Rate
Exonic Reads/Mapped Reads
0.6 0.7 0.8 0.9
Intronic Rate
Intronic Reads/Mapped Reads
0.05 0.15 0.25 0.35
Intergenic Rate
Intergenic Reads/Mapped Reads
0.01 0.02 0.03 0.04 0.05 0.06
Per Base Coverage
Mean Per Base Coverage
0.002 0.003 0.004 0.005 0.006
Expression Profiling Efficiency
Exonic Reads/Total Reads
0.6 0.7 0.8 0.9
Standard Deviation of Fragment Length
StdDev(Fragment Length)
120 140 160 180 200 220
Fragment Length
Mean(Fragment Length)
120 160 200 240
rRNA rate
rRNA Reads/Total Reads
0.000 0.005 0.010 0.015
5' 100base Normalized Coverage
Per Base Coverage at 5' End
300 500 700 900
End 1 Mismatch Rate
End1 Mismatch Bases/End1 Bases
0.002 0.003 0.004 0.005
End 2 Mismatch Rate
End2 Mismatch Bases/End2 Bases
0.002 0.003 0.004 0.005 0.006
Base Mismatch Rate
Mismatch Bases/Aligned Bases
0.002 0.003 0.004 0.005 0.006
End 1 Mapping Rate
End1 Mapped/End1 Reads
0.99 1.00 1.01 1.02
End 2 Mapping Rate
End2 Mapped/End2 Reads
0.98 0.99 1.00 1.01
Adipose
Heart
Kidney
LCL
Liver

S9

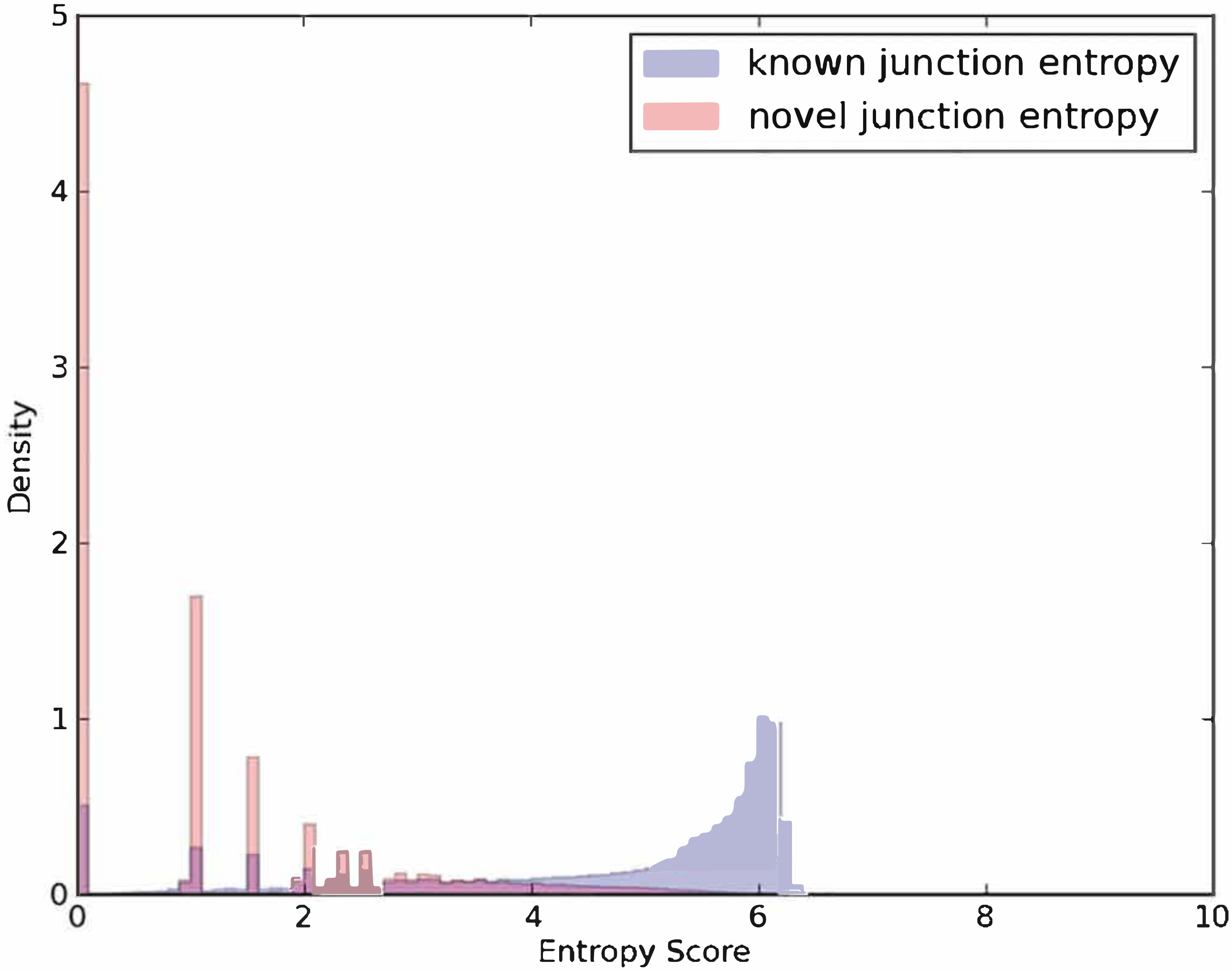

known junction entropy
novel junction entropy
Density
Entropy Score
0
1
2
3
4
5
0
2
4
6
8
10

S10

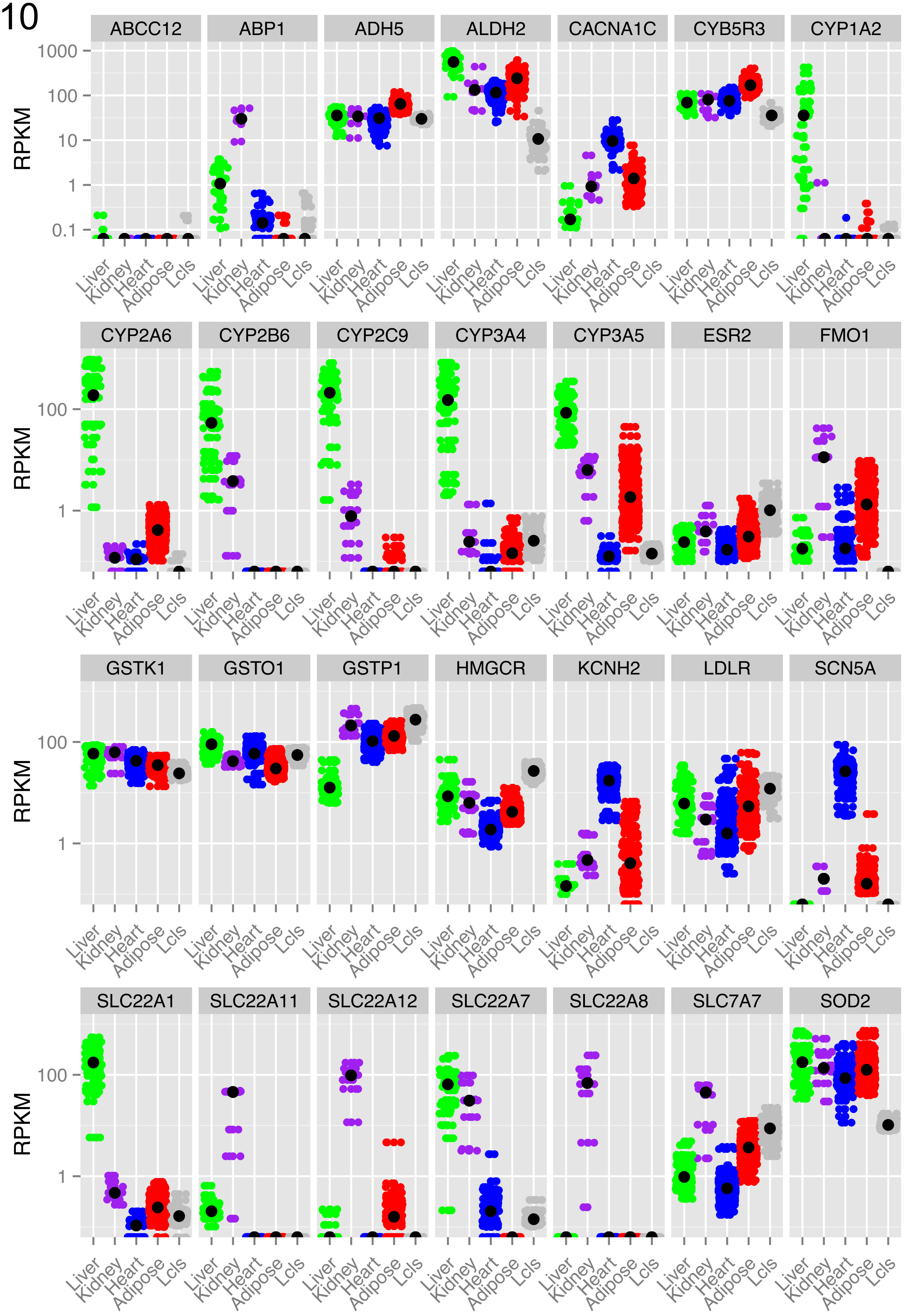
ABCC12
ABP1
ADH5
ALDH2
CACNA1C
CYB5R3
CYP1A2
CYP2A6
CYP2B6
CYP2C9
CYP3A4
CYP3A5
ESR2
FMO1
GSTK1
GSTO1
GSTP1
HMGCR
KCNH2
LDLR
SCN5A
SLC22A1
SLC22A11
SLC22A12
SLC22A7
SLC22A8
SLC7A7
SOD2
RPKM
1000
100
10
1
0.1
Liver
Kidney
Heart
Adipose
Lcls

Supplemental Table S1. List of 389 pharmacogenes and their categories based on their functions. Annotation for drug target is compiled from Rask-Anderson, M et al. The druggable genome: Evaluation of drug targets in clinical trials suggests major shifts in molecular class and indication. Annu Rev Pharmacol Toxicol. 54, 9-26 (2014).

Number	Gene	Ensembl Gene ID	Category	Drug Target	GPCR as Drug Target	Enzyme as Drug Target	Ion Channel as Drug Target
1	ABCA1	ENSG00000165029.11	ABC_Transporter	N	N	N	N
2	ABCA4	ENSG00000198691.6	ABC_Transporter	Y	N	N	N
3	ABCB1	ENSG00000085563.8	ABC_Transporter	Y	N	N	N
4	ABCB10	ENSG00000135776.4	ABC_Transporter	N	N	N	N
5	ABCB11	ENSG00000073734.8	ABC_Transporter	N	N	N	N
6	ABCB4	ENSG00000005471.11	ABC_Transporter	N	N	N	N
7	ABCB5	ENSG00000004846.12	ABC_Transporter	N	N	N	N
8	ABCB6	ENSG00000115657.7	ABC_Transporter	N	N	N	N
9	ABCB7	ENSG00000131269.10	ABC_Transporter	N	N	N	N
10	ABCB8	ENSG00000197150.7	ABC_Transporter	N	N	N	N
11	ABCB9	ENSG00000150967.12	ABC_Transporter	N	N	N	N
12	ABCC1	ENSG00000103222.13	ABC_Transporter	Y	N	N	N
13	ABCC10	ENSG00000124574.9	ABC_Transporter	N	N	N	N
14	ABCC12	ENSG00000140798.10	ABC_Transporter	N	N	N	N
15	ABCC2	ENSG00000023839.6	ABC_Transporter	Y	N	N	N
16	ABCC3	ENSG00000108846.11	ABC_Transporter	N	N	N	N
17	ABCC4	ENSG00000125257.9	ABC_Transporter	N	N	N	N
18	ABCC5	ENSG00000114770.11	ABC_Transporter	N	N	N	N
19	ABCC6	ENSG00000091262.9	ABC_Transporter	N	N	N	N
20	ABCC8	ENSG00000006071.6	ABC_Transporter	Y	N	N	N
21	ABCC9	ENSG00000069431.6	ABC_Transporter	N	N	N	N
22	ABCG2	ENSG00000118777.6	ABC_Transporter	N	N	N	N
23	ABCG8	ENSG00000143921.6	ABC_Transporter	N	N	N	N
24	ABP1	ENSG00000002726.14	Other_Metabolism	N	N	N	N
25	AC008537.2	ENSG00000256612.1	Other	N	N	N	N
26	ACE	ENSG00000159640.8	Other	Y	N	Y	N
27	ADD1	ENSG00000087274.11	Other	N	N	N	N
28	ADH1A	ENSG00000187758.3	ADH_Metabolism	Y	N	Y	N
29	ADH1B	ENSG00000196616.7	ADH_Metabolism	Y	N	Y	N
30	ADH1C	ENSG00000248144.1	ADH_Metabolism	Y	N	Y	N
31	ADH4	ENSG00000198099.4	ADH_Metabolism	N	N	Y	N
32	ADH5	ENSG00000197894.6	ADH_Metabolism	N	N	Y	N
33	ADH6	ENSG00000172955.13	ADH_Metabolism	N	N	Y	N
34	ADH7	ENSG00000196344.7	ADH_Metabolism	N	N	Y	N
35	ADRA1B	ENSG00000170214.3	Receptor	Y	Y	N	N
36	ADRA2C	ENSG00000184160.6	Receptor	Y	Y	N	N
37	ADRB1	ENSG00000043591.4	Receptor	Y	Y	N	N

38	ADRB2	ENSG00000169252.4	Receptor	Y	Y	N	N
39	ADRB3	ENSG00000188778.3	Receptor	Y	Y	N	N
40	AGTR1	ENSG00000144891.13	Receptor	Y	Y	N	N
41	AHR	ENSG00000106546.8	Nuclear Receptor/Transcription Factor	N	N	N	N
42	AKAP9	ENSG00000127914.11	Other	N	N	N	N
43	ALB	ENSG00000163631.11	Other	N	N	N	N
44	ALDH1A1	ENSG00000165092.7	ALDH_Metabolism	N	N	Y	N
45	ALDH2	ENSG00000111275.6	ALDH_Metabolism	Y	N	Y	N
46	ALDH3A1	ENSG00000108602.12	ALDH_Metabolism	N	N	Y	N
47	ALDH3A2	ENSG00000072210.12	ALDH_Metabolism	N	N	Y	N
48	ALDH4A1	ENSG00000159423.11	ALDH_Metabolism	N	N	Y	N
49	ALDH5A1	ENSG00000112294.8	ALDH_Metabolism	N	N	Y	N
50	ALDH6A1	ENSG00000119711.8	ALDH_Metabolism	N	N	Y	N
51	ALOX5	ENSG00000012779.5	Other	Y	N	N	N
52	AOX1	ENSG00000138356.9	Other_Metabolism	N	N	N	N
53	APOA1	ENSG00000118137.5	Other	Y	N	N	N
54	APOA2	ENSG00000158874.6	Other	N	N	N	N
55	APOA4	ENSG00000110244.5	Other	N	N	N	N
56	APOB	ENSG00000084674.8	Other	Y	N	N	N
57	APOE	ENSG00000130203.4	Other	N	N	N	N
58	ARID5B	ENSG00000150347.10	Other	N	N	N	N
59	ARNT	ENSG00000143437.14	Nuclear Receptor/Transcription Factor	N	N	N	N
60	ARSA	ENSG00000100299.12	Other	N	N	N	N
61	ATP7A	ENSG00000165240.12	Other_Transporter	N	N	N	N
62	ATP7B	ENSG00000123191.9	Other_Transporter	N	N	N	N
63	BDKRB2	ENSG00000168398.5	Receptor	Y	Y	N	N
64	BDNF	ENSG00000176697.12	Other	N	N	N	N
65	BRCA1	ENSG00000012048.13	Other	N	N	N	N
66	BRCA2	ENSG00000139618.9	Other	N	N	N	N
67	CACNA1C	ENSG00000151067.14	Channel	Y	N	N	Y
68	CACNA1S	ENSG00000081248.6	Channel	Y	N	N	Y
69	CACNB2	ENSG00000165995.14	Channel	Y	N	N	Y
70	CBR1	ENSG00000159228.8	Other_Metabolism	N	N	N	N
71	CBR3	ENSG00000159231.5	Other_Metabolism	N	N	N	N
72	CCDC101	ENSG00000176476.4	Other	N	N	N	N
73	CCND1	ENSG00000110092.2	Other	Y	N	N	N
74	CDA	ENSG00000158825.5	Other_Metabolism	N	N	N	N
75	CEBPA	ENSG00000245848.2	Nuclear Receptor/Transcription Factor	N	N	N	N
76	CEBPB	ENSG00000172216.4	Nuclear Receptor/Transcription Factor	N	N	N	N

77	CES1	ENSG00000198848.7	Other_Metabolism	N	N	N	N
78	CES2	ENSG00000172831.6	Other_Metabolism	N	N	N	N
79	CETP	ENSG00000087237.6	Other	Y	N	N	N
80	CFTR	ENSG00000001626	ABC_Transporter	Y	Y	N	N
81	CHRM3	ENSG00000133019.7	Receptor	Y	Y	N	N
82	CHST1	ENSG00000175264.3	Other	N	N	N	N
83	CHST10	ENSG00000115526.6	Other	N	N	N	N
84	CHST11	ENSG00000171310.6	Other	N	N	N	N
85	CHST13	ENSG00000180767.4	Other	N	N	N	N
86	CHST2	ENSG00000175040.4	Other	N	N	N	N
87	CHST3	ENSG00000122863.5	Other	N	N	N	N
88	CHST4	ENSG00000140835.7	Other	N	N	N	N
89	CHST5	ENSG00000135702.10	Other	N	N	N	N
90	CHST6	ENSG00000183196.4	Other	N	N	N	N
91	CHST7	ENSG00000147119.3	Other	N	N	N	N
92	CHST8	ENSG00000124302.5	Other	N	N	N	N
93	CHST9	ENSG00000154080.7	Other	N	N	N	N
94	COL1A1	ENSG00000108821.8	Other	Y	N	N	N
95	COMT	ENSG00000093010.7	Other_Metabolism	Y	N	Y	N
96	CR1	ENSG00000203710.5	Receptor	N	N	N	N
97	CRHR1	ENSG00000120088.9	Receptor	Y	Y	N	N
98	CROT	ENSG00000005469.7	Other	Y	N	N	N
99	CYB5A	ENSG00000166347.12	Other_Metabolism	N	N	N	N
100	CYB5B	ENSG00000103018.11	Other_Metabolism	N	N	N	N
101	CYB5R3	ENSG00000100243.15	Other_Metabolism	N	N	N	N
102	CYP11A1	ENSG00000140459.11	CYP_Metabolism	N	N	N	N
103	CYP11B1	ENSG00000160882.7	CYP_Metabolism	Y	N	Y	N
104	CYP11B2	ENSG00000179142.2	CYP_Metabolism	N	N	N	N
105	CYP17A1	ENSG00000148795.4	CYP_Metabolism	Y	N	Y	N
106	CYP19A1	ENSG00000137869.8	CYP_Metabolism	Y	N	Y	N
107	CYP1A1	ENSG00000140465.8	CYP_Metabolism	N	N	N	N
108	CYP1A2	ENSG00000140505.6	CYP_Metabolism	N	N	N	N
109	CYP1B1	ENSG00000138061.7	CYP_Metabolism	Y	N	Y	N
110	CYP20A1	ENSG00000119004.9	CYP_Metabolism	N	N	N	N
111	CYP21A2	ENSG00000231852.2	CYP_Metabolism	N	N	N	N
112	CYP24A1	ENSG00000019186.5	CYP_Metabolism	Y	N	Y	N
113	CYP26A1	ENSG00000095596.7	CYP_Metabolism	Y	N	Y	N
114	CYP26C1	ENSG00000187553.5	CYP_Metabolism	Y	N	Y	N
115	CYP27A1	ENSG00000135929.4	CYP_Metabolism	N	N	N	N
116	CYP27B1	ENSG00000111012.4	CYP_Metabolism	N	N	N	N
117	CYP2A13	ENSG00000197838.3	CYP_Metabolism	N	N	N	N
118	CYP2A6	ENSG00000255974.1	CYP_Metabolism	N	N	N	N
119	CYP2A7	ENSG00000198077.5	CYP_Metabolism	N	N	N	N

120	CYP2B6	ENSG00000197408.3	CYP_Metabolism	N	N	N	N
121	CYP2C18	ENSG00000108242.7	CYP_Metabolism	N	N	N	N
122	CYP2C19	ENSG00000165841.5	CYP_Metabolism	N	N	N	N
123	CYP2C8	ENSG00000138115.7	CYP_Metabolism	N	N	N	N
124	CYP2C9	ENSG00000138109.8	CYP_Metabolism	N	N	N	N
125	CYP2D6	ENSG00000100197.15	CYP_Metabolism	N	N	N	N
126	CYP2E1	ENSG00000130649.5	CYP_Metabolism	N	N	N	N
127	CYP2F1	ENSG00000197446.3	CYP_Metabolism	N	N	N	N
128	CYP2J2	ENSG00000134716.5	CYP_Metabolism	N	N	N	N
129	CYP2R1	ENSG00000186104.6	CYP_Metabolism	N	N	N	N
130	CYP2S1	ENSG00000167600.7	CYP_Metabolism	N	N	N	N
131	CYP39A1	ENSG00000146233.3	CYP_Metabolism	N	N	N	N
132	CYP3A4	ENSG00000160868.9	CYP_Metabolism	Y	N	Y	N
133	CYP3A43	ENSG00000021461.11	CYP_Metabolism	N	N	N	N
134	CYP3A5	ENSG00000106258.9	CYP_Metabolism	Y	N	Y	N
135	CYP3A7	ENSG00000160870.7	CYP_Metabolism	Y	N	Y	N
136	CYP46A1	ENSG00000036530.4	CYP_Metabolism	Y	N	Y	N
137	CYP4A11	ENSG00000187048.8	CYP_Metabolism	N	N	N	N
138	CYP4B1	ENSG00000142973.8	CYP_Metabolism	N	N	N	N
139	CYP4F11	ENSG00000171903.10	CYP_Metabolism	N	N	N	N
140	CYP4F12	ENSG00000186204.8	CYP_Metabolism	N	N	N	N
141	CYP4F2	ENSG00000186115.5	CYP_Metabolism	N	N	N	N
142	CYP4F3	ENSG00000186529.7	CYP_Metabolism	N	N	N	N
143	CYP4F8	ENSG00000186526.6	CYP_Metabolism	N	N	N	N
144	CYP4Z1	ENSG00000186160.4	CYP_Metabolism	N	N	N	N
145	CYP51A1	ENSG00000001630.10	CYP_Metabolism	N	N	N	N
146	CYP7A1	ENSG00000167910.3	CYP_Metabolism	N	N	N	N
147	CYP7B1	ENSG00000172817.3	CYP_Metabolism	N	N	N	N
148	CYP8B1	ENSG00000180432.4	CYP_Metabolism	N	N	N	N
149	DBH	ENSG00000123454.5	Other_Metabolism	Y	N	Y	N
150	DCK	ENSG00000156136.5	Other_Metabolism	Y	N	Y	N
151	DHFR	ENSG00000228716.2	Other_Metabolism	Y	N	Y	N
152	DPYD	ENSG00000188641.8	Other_Metabolism	Y	N	Y	N
153	DRD1	ENSG00000184845.2	Receptor	Y	Y	N	N
154	DRD2	ENSG00000149295.9	Receptor	Y	Y	N	N
155	DRD3	ENSG00000151577.6	Receptor	Y	Y	N	N
156	DRD4	ENSG00000069696.6	Receptor	Y	Y	N	N
157	DRD5	ENSG00000169676.4	Receptor	Y	Y	N	N
158	EGFR	ENSG00000146648.10	Receptor	Y	N	N	N
159	EPHX1	ENSG00000143819.8	Other_Metabolism	Y	N	Y	N
160	EPHX2	ENSG00000120915.8	Other_Metabolism	Y	N	Y	N
161	ESR1	ENSG00000091831.15	Receptor	Y	N	N	N
162	ESR2	ENSG00000140009.14	Receptor	Y	N	N	N

163	F2	ENSG00000180210.9	Other	Y	N	N	N
164	F5	ENSG00000198734.6	Other	Y	N	N	N
165	FAAH	ENSG00000117480.10	Other_Metabolism	Y	N	Y	N
166	FCGR2A	ENSG00000143226.8	Receptor	N	N	N	N
167	FCGR3A	ENSG00000203747.5	Receptor	N	N	N	N
168	FKBP5	ENSG00000096060.8	Other	N	N	N	N
169	FMO1	ENSG00000010932.11	Other_Metabolism	N	N	N	N
170	FMO2	ENSG00000094963.9	Other_Metabolism	N	N	N	N
171	FMO3	ENSG00000007933.8	Other_Metabolism	N	N	N	N
172	FMO4	ENSG00000076258.5	Other_Metabolism	N	N	N	N
173	FMO5	ENSG00000131781.8	Other_Metabolism	N	N	N	N
174	FMO6P	ENSG00000117507.3	Other_Metabolism	N	N	N	N
175	FOXA3	ENSG00000170608.1	Nuclear Receptor/Transcription Factor	N	N	N	N
176	G6PD	ENSG00000160211.10	Other_Metabolism	N	N	N	N
177	GLCCI1	ENSG00000106415.8	Other	N	N	N	N
178	GNB3	ENSG00000111664.6	Other	N	N	N	N
179	GPX7	ENSG00000116157.5	Other_Metabolism	N	N	N	N
180	GRK4	ENSG00000125388.14	Other	N	N	N	N
181	GRK5	ENSG00000198873.8	Other	N	N	N	N
182	GRM3	ENSG00000198822.6	Receptor	Y	Y	N	N
183	GSR	ENSG00000104687.8	Other_Metabolism	Y	N	Y	N
184	GSTA1	ENSG00000243955.1	GST_Metabolism	N	N	N	N
185	GSTA2	ENSG00000244067.1	GST_Metabolism	N	N	N	N
186	GSTA3	ENSG00000174156.7	GST_Metabolism	N	N	N	N
187	GSTA4	ENSG00000170899.6	GST_Metabolism	N	N	N	N
188	GSTA5	ENSG00000182793.5	GST_Metabolism	N	N	N	N
189	GSTK1	ENSG00000197448.9	GST_Metabolism	N	N	N	N
190	GSTM1	ENSG00000134184.7	GST_Metabolism	N	N	N	N
191	GSTM2	ENSG00000213366.6	GST_Metabolism	N	N	N	N
192	GSTM3	ENSG00000134202.6	GST_Metabolism	N	N	N	N
193	GSTM4	ENSG00000168765.10	GST_Metabolism	N	N	N	N
194	GSTM5	ENSG00000134201.5	GST_Metabolism	N	N	N	N
195	GSTO1	ENSG00000148834.8	GST_Metabolism	N	N	N	N
196	GSTO2	ENSG00000065621.8	GST_Metabolism	N	N	N	N
197	GSTP1	ENSG00000084207.11	GST_Metabolism	Y	N	Y	N
198	GSTT1	ENSG00000184674.7	GST_Metabolism	N	N	N	N
199	GSTT2	ENSG00000099984.6	GST_Metabolism	N	N	N	N
200	GSTZ1	ENSG00000100577.14	GST_Metabolism	N	N	N	N
201	HLA-B	ENSG00000234745.3	Other	N	N	N	N
202	HMGCR	ENSG00000113161.10	Other	Y	N	N	N
203	HNF1A	ENSG00000135100.9	Nuclear Receptor/Transcription Factor	N	N	N	N

204	HNF4A	ENSG00000101076.10	Nuclear Receptor/Transcription Factor	N	N	N	N
205	HNMT	ENSG00000150540.9	Other_Metabolism	N	N	N	N
206	HSD11B2	ENSG00000176387.5	Other_Metabolism	N	N	N	N
207	HTR1A	ENSG00000178394.3	Receptor	Y	Y	N	N
208	HTR2A	ENSG00000102468.5	Receptor	Y	Y	N	N
209	IL10	ENSG00000136634.5	Other	N	N	N	N
210	IL1B	ENSG00000125538.7	Other	Y	N	N	N
211	IL28B	ENSG00000197110	Other	N	N	N	N
212	INPP1	ENSG00000151689.8	Other	N	N	N	N
213	JUN	ENSG00000177606.5	Nuclear Receptor/Transcription Factor	Y	N	N	N
214	KCNE1	ENSG00000180509.7	Channel	Y	N	N	Y
215	KCNE2	ENSG00000159197.2	Channel	N	N	N	N
216	KCNH2	ENSG00000055118.9	Channel	Y	N	N	Y
217	KCNJ11	ENSG00000187486.5	Channel	Y	N	N	Y
218	KCNQ1	ENSG00000053918.10	Channel	Y	N	N	Y
219	LDLR	ENSG00000130164.6	Receptor	N	N	N	N
220	LPL	ENSG00000175445.9	Other	Y	N	N	N
221	LTC4S	ENSG00000213316.5	Other	N	N	N	N
222	MAOA	ENSG00000189221.5	Other_Metabolism	Y	N	Y	N
223	MAOB	ENSG00000069535.11	Other_Metabolism	Y	N	Y	N
224	MAT1A	ENSG00000151224.7	Other	N	N	N	N
225	METTL1	ENSG00000037897.12	Other	N	N	N	N
226	MLH1	ENSG00000076242.9	Other	N	N	N	N
227	MMP3	ENSG00000149968.7	Other	Y	N	N	N
228	MTHFR	ENSG00000177000.6	Other_Metabolism	N	N	N	N
229	NAT1	ENSG00000171428.9	Other_Metabolism	N	N	N	N
230	NAT2	ENSG00000156006.4	Other_Metabolism	N	N	N	N
231	NCOR1	ENSG00000141027.13	Nuclear Receptor/Transcription Factor	N	N	N	N
232	NNMT	ENSG00000166741.3	Other_Metabolism	Y	N	Y	N
233	NOS3	ENSG00000164867.5	Other_Metabolism	Y	N	Y	N
234	NPPB	ENSG00000120937.7	Other	N	N	N	N
235	NPR1	ENSG00000169418.7	Receptor	Y	Y	N	N
236	NQO1	ENSG00000181019.8	Other_Metabolism	Y	N	Y	N
237	NQO2	ENSG00000124588.13	Other_Metabolism	Y	N	Y	N
238	NR0B2	ENSG00000131910.4	Nuclear Receptor/Transcription Factor	N	N	N	N
239	NR1H2	ENSG00000131408.7	Nuclear Receptor/Transcription Factor	N	N	N	N
240	NR1H3	ENSG00000025434.14	Nuclear Receptor/Transcription Factor	N	N	N	N

241	NR1H4	ENSG00000012504.9	Nuclear Receptor/Transcription Factor	Y	N	N	N
242	NR1I2	ENSG00000144852.12	Nuclear Receptor/Transcription Factor	N	N	N	N
243	NR1I3	ENSG00000143257.7	Nuclear Receptor/Transcription Factor	N	N	N	N
244	NR3C1	ENSG00000113580.9	Nuclear Receptor/Transcription Factor	Y	N	N	N
245	NR3C2	ENSG00000151623.9	Nuclear Receptor/Transcription Factor	Y	N	N	N
246	OPRM1	ENSG00000112038.12	Receptor	Y	Y	N	N
247	ORM1	ENSG00000229314.4	Other	N	N	N	N
248	ORM2	ENSG00000228278.2	Other	N	N	N	N
249	P2RY1	ENSG00000169860.4	Receptor	Y	Y	N	N
250	P2RY12	ENSG00000169313.8	Receptor	Y	Y	N	N
251	PARK2	ENSG00000185345.13	Other	N	N	N	N
252	PEAR1	ENSG00000187800.9	Receptor	N	N	N	N
253	PGAP3	ENSG00000161395.7	Other	N	N	N	N
254	PNMT	ENSG00000141744.2	Other_Metabolism	N	N	N	N
255	PON1	ENSG00000005421.4	Other_Metabolism	N	N	N	N
256	PON2	ENSG00000105854.7	Other_Metabolism	N	N	N	N
257	PON3	ENSG00000105852.6	Other_Metabolism	N	N	N	N
258	POR	ENSG00000127948.9	Other_Metabolism	N	N	N	N
259	PPARA	ENSG00000186951.11	Nuclear Receptor/Transcription Factor	Y	N	N	N
260	PPARD	ENSG00000112033.9	Nuclear Receptor/Transcription Factor	Y	N	N	N
261	PPARG	ENSG00000132170.15	Nuclear Receptor/Transcription Factor	Y	N	N	N
262	PPARGC1A	ENSG00000109819.4	Nuclear Receptor/Transcription Factor	N	N	N	N
263	PPP1R9A	ENSG00000158528.7	Other	N	N	N	N
264	PRSS53	ENSG00000151006.7	Other	N	N	N	N
265	PSMB8	ENSG00000204264.4	Other	N	N	N	N
266	PTGIS	ENSG00000124212.3	Other_Metabolism	Y	N	Y	N
267	PTGS1	ENSG00000095303.10	Other_Metabolism	Y	N	Y	N
268	PTGS2	ENSG00000073756.7	Other_Metabolism	Y	N	Y	N
269	QPRT	ENSG00000103485.12	Other_Metabolism	Y	N	Y	N
270	RALBP1	ENSG00000017797.6	Other_Transporter	N	N	N	N
271	RPL13	ENSG00000167526.9	Other	N	N	N	N
272	RXRA	ENSG00000186350.8	Nuclear Receptor/Transcription Factor	Y	N	N	N

273	RYR1	ENSG00000196218.5	Channel	Y	N	N	Y
274	RYR2	ENSG00000198626.9	Channel	N	N	N	N
275	SCN1B	ENSG00000105711.5	Channel	Y	N	N	Y
276	SCN2B	ENSG00000149575.5	Channel	Y	N	N	Y
277	SCN3B	ENSG00000166257.4	Channel	Y	N	N	Y
278	SCN4B	ENSG00000177098.4	Channel	Y	N	N	Y
279	SCN5A	ENSG00000183873.10	Channel	Y	N	N	Y
280	SERPINA1	ENSG00000197249.8	Other	N	N	N	N
281	SERPINA7	ENSG00000123561.10	Other	N	N	N	N
282	SLC10A1	ENSG00000100652.4	SLC_Transporter	N	N	N	N
283	SLC10A2	ENSG00000125255.5	SLC_Transporter	N	N	N	N
284	SLC13A1	ENSG00000081800.4	SLC_Transporter	N	N	N	N
285	SLC13A2	ENSG00000007216.9	SLC_Transporter	N	N	N	N
286	SLC13A3	ENSG00000158296.9	SLC_Transporter	N	N	N	N
287	SLC15A1	ENSG00000088386.9	SLC_Transporter	N	N	N	N
288	SLC15A2	ENSG00000163406.5	SLC_Transporter	N	N	N	N
289	SLC16A1	ENSG00000155380.7	SLC_Transporter	N	N	N	N
290	SLC19A1	ENSG00000173638.13	SLC_Transporter	N	N	N	N
291	SLC22A1	ENSG00000175003.8	SLC_Transporter	N	N	N	N
292	SLC22A10	ENSG00000184999.7	SLC_Transporter	N	N	N	N
293	SLC22A11	ENSG00000168065.11	SLC_Transporter	Y	N	N	N
294	SLC22A12	ENSG00000197891.7	SLC_Transporter	Y	N	N	N
295	SLC22A13	ENSG00000172940.7	SLC_Transporter	N	N	N	N
296	SLC22A14	ENSG00000144671.5	SLC_Transporter	N	N	N	N
297	SLC22A15	ENSG00000163393.8	SLC_Transporter	N	N	N	N
298	SLC22A16	ENSG00000004809.9	SLC_Transporter	N	N	N	N
299	SLC22A17	ENSG00000092096.10	SLC_Transporter	N	N	N	N
300	SLC22A18	ENSG00000110628.9	SLC_Transporter	N	N	N	N
301	SLC22A18AS	ENSG00000254827.1	SLC_Transporter	N	N	N	N
302	SLC22A2	ENSG00000112499.7	SLC_Transporter	N	N	N	N
303	SLC22A3	ENSG00000146477.4	SLC_Transporter	N	N	N	N
304	SLC22A4	ENSG00000197208.5	SLC_Transporter	Y	N	N	N
305	SLC22A5	ENSG00000197375.7	SLC_Transporter	Y	N	N	N
306	SLC22A6	ENSG00000197901.6	SLC_Transporter	N	N	N	N
307	SLC22A7	ENSG00000137204.10	SLC_Transporter	N	N	N	N
308	SLC22A8	ENSG00000149452.10	SLC_Transporter	Y	N	N	N
309	SLC22A9	ENSG00000149742.5	SLC_Transporter	N	N	N	N
310	SLC25A27	ENSG00000153291.8	SLC_Transporter	N	N	N	N
311	SLC27A1	ENSG00000130304.10	SLC_Transporter	N	N	N	N
312	SLC28A1	ENSG00000156222.7	SLC_Transporter	N	N	N	N
313	SLC28A2	ENSG00000137860.7	SLC_Transporter	N	N	N	N
314	SLC28A3	ENSG00000197506.6	SLC_Transporter	N	N	N	N
315	SLC29A1	ENSG00000112759.11	SLC_Transporter	N	N	N	N

316	SLC29A2	ENSG00000174669.7	SLC_Transporter	N	N	N	N
317	SLC2A4	ENSG00000181856.10	SLC_Transporter	Y	N	N	N
318	SLC2A5	ENSG00000142583.11	SLC_Transporter	Y	N	N	N
319	SLC47A1	ENSG00000142494.7	SLC_Transporter	N	N	N	N
320	SLC5A6	ENSG00000138074.9	SLC_Transporter	N	N	N	N
321	SLC6A3	ENSG00000142319.14	SLC_Transporter	Y	N	N	N
322	SLC6A4	ENSG00000108576.4	SLC_Transporter	Y	N	N	N
323	SLC6A6	ENSG00000131389.12	SLC_Transporter	N	N	N	N
324	SLC7A5	ENSG00000103257.4	SLC_Transporter	N	N	N	N
325	SLC7A7	ENSG00000155465.13	SLC_Transporter	N	N	N	N
326	SLC7A8	ENSG00000092068.13	SLC_Transporter	N	N	N	N
327	SLCO1A2	ENSG00000084453.12	SLC_Transporter	N	N	N	N
328	SLCO1B1	ENSG00000134538.2	SLC_Transporter	N	N	N	N
329	SLCO1B3	ENSG00000111700.8	SLC_Transporter	N	N	N	N
330	SLCO1C1	ENSG00000139155.4	SLC_Transporter	N	N	N	N
331	SLCO2A1	ENSG00000174640.8	SLC_Transporter	N	N	N	N
332	SLCO2B1	ENSG00000137491.9	SLC_Transporter	N	N	N	N
333	SLCO3A1	ENSG00000176463.8	SLC_Transporter	N	N	N	N
334	SLCO4A1	ENSG00000101187.10	SLC_Transporter	N	N	N	N
335	SLCO4C1	ENSG00000173930.8	SLC_Transporter	N	N	N	N
336	SLCO5A1	ENSG00000137571.5	SLC_Transporter	N	N	N	N
337	SLCO6A1	ENSG00000205359.5	SLC_Transporter	N	N	N	N
338	SOD2	ENSG00000112096.11	Other_Metabolism	N	N	N	N
339	SPG7	ENSG00000197912.8	Other	N	N	N	N
340	SPN	ENSG00000197471.6	Other	N	N	N	N
341	SPP1	ENSG00000118785.8	Other	N	N	N	N
342	SULT1A1	ENSG00000196502.7	SULT_Metabolism	N	N	N	N
343	SULT1A2	ENSG00000197165.6	SULT_Metabolism	N	N	N	N
344	SULT1A3	ENSG00000261052.1	SULT_Metabolism	N	N	N	N
345	SULT1B1	ENSG00000173597.3	SULT_Metabolism	N	N	N	N
346	SULT1C2	ENSG00000198203.5	SULT_Metabolism	N	N	N	N
347	SULT1C4	ENSG00000198075.5	SULT_Metabolism	N	N	N	N
348	SULT1E1	ENSG00000109193.6	SULT_Metabolism	N	N	N	N
349	SULT2A1	ENSG00000105398.3	SULT_Metabolism	N	N	N	N
350	SULT2B1	ENSG00000088002.6	SULT_Metabolism	N	N	N	N
351	SULT4A1	ENSG00000130540.9	SULT_Metabolism	N	N	N	N
352	TAP1	ENSG00000168394.9	Other_Transporter	N	N	N	N
353	TBXAS1	ENSG00000059377.10	Other_Metabolism	Y	N	Y	N
354	TCL1A	ENSG00000100721.6	Other	N	N	N	N
355	TGFB1	ENSG00000105329.4	Other	N	N	N	N
356	TGFB2	ENSG00000092969.7	Other	Y	N	N	N
357	TGFB3	ENSG00000119699.3	Other	N	N	N	N
358	TGFBI	ENSG00000120708.9	Other	N	N	N	N

359	TNF	ENSG00000232810.3	Other	Y	N	N	N
360	TNFRSF1A	ENSG00000067182.3	Other	Y	N	N	N
361	TNFRSF1B	ENSG00000028137.11	Other	Y	N	N	N
362	TPH1	ENSG00000129167.5	Other_Metabolism	Y	N	Y	N
363	TPH2	ENSG00000139287.7	Other_Metabolism	N	N	N	N
364	TPMT	ENSG00000137364.4	Other_Metabolism	N	N	N	N
365	TPSG1	ENSG00000116176.6	Other	N	N	N	N
366	TYMS	ENSG00000176890.10	Other_Metabolism	Y	N	Y	N
367	UGT1A1	ENSG00000241635.2	UGT_Metabolism	N	N	N	N
368	UGT1A10	ENSG00000242515.1	UGT_Metabolism	N	N	N	N
369	UGT1A3	ENSG00000243135.1	UGT_Metabolism	N	N	N	N
370	UGT1A4	ENSG00000244474.1	UGT_Metabolism	N	N	N	N
371	UGT1A5	ENSG00000240224.1	UGT_Metabolism	N	N	N	N
372	UGT1A6	ENSG00000167165.11	UGT_Metabolism	N	N	N	N
373	UGT1A7	ENSG00000244122.2	UGT_Metabolism	N	N	N	N
374	UGT1A8	ENSG00000242366.1	UGT_Metabolism	N	N	N	N
375	UGT1A9	ENSG00000241119.1	UGT_Metabolism	N	N	N	N
376	UGT2A1	ENSG00000173610.6	UGT_Metabolism	N	N	N	N
377	UGT2B10	ENSG00000109181.7	UGT_Metabolism	N	N	N	N
378	UGT2B11	ENSG00000213759.4	UGT_Metabolism	N	N	N	N
379	UGT2B15	ENSG00000196620.4	UGT_Metabolism	N	N	N	N
380	UGT2B17	ENSG00000197888.2	UGT_Metabolism	N	N	N	N
381	UGT2B28	ENSG00000135226.12	UGT_Metabolism	N	N	N	N
382	UGT2B4	ENSG00000156096.8	UGT_Metabolism	N	N	N	N
383	UGT2B7	ENSG00000171234.9	UGT_Metabolism	N	N	N	N
384	UGT8	ENSG00000174607.6	UGT_Metabolism	N	N	N	N
385	VDR	ENSG00000111424.6	Nuclear Receptor/Transcription Factor	Y	N	N	N
386	VKORC1	ENSG00000167397.10	Other	Y	N	N	N
387	XDH	ENSG00000158125.5	Other_Metabolism	Y	N	Y	N
388	XRCC1	ENSG00000073050.5	Other	N	N	N	N
389	ZNF423	ENSG00000102935.7	Nuclear Receptor/Transcription Factor	N	N	N	N

Supplemental Table S2. The population demographic information of the human (i) liver tissues, (ii) kidney tissues, (iii) heart atrial tissues, (iv) adipose tissue and (v) lymphoblastoid cell lines

(i) Human liver tissues

Number	Tissue	Age	Sex	Race	Tissue Source	Selected 18 samples for subsampling analysis	Number of Raw Reads	Number of Unique Mapped Reads
1	Liver	75	Female	Caucasian	These samples were normal postmortem human liver that were obtained through the Liver Tissue Procurement and Distribution System (University of Pittsburgh, Pennsylvania). Ref: Schadt, EE et al. Mapping the genetic architecture of gene expression in human liver. PLoS Biol. 6(5), e107 (2008).	Y	74067026	34956769
2	Liver	72	Male	Caucasian		Y	84596342	45728515
3	Liver	68	Female	Caucasian		N	104114760	17720652
4	Liver	62	Male	Caucasian		Y	68202500	35937587
5	Liver	53	Male	Caucasian		Y	80599340	44339114
6	Liver	28	Male	Caucasian		N	59695274	15933223
7	Liver	45	Male	Caucasian		Y	77870534	28870565
8	Liver	54	Male	Caucasian		Y	94573624	45599306
9	Liver	60	Male	Caucasian		N	63973708	14390113
10	Liver	60	Male	Caucasian		N	70802816	21585499
11	Liver	67	Male	Caucasian		Y	67874018	35489635
12	Liver	15	Female	Caucasian		Y	73733946	38910060
13	Liver	66	Female	Caucasian		Y	69714872	32125879
14	Liver	57	Male	Caucasian		Y	71821034	35512918
15	Liver	49	Male	Caucasian		Y	91979674	53216324
16	Liver	68	Male	Caucasian		Y	71825386	43402609
17	Liver	16	Female	Caucasian		Y	62880672	26062674
18	Liver	75	Female	Caucasian		N	51671476	9583751
19	Liver	30	Female	Caucasian		N	93870408	45110798
20	Liver	47	Female	Caucasian		Y	87331202	52582049
21	Liver	53	Male	Caucasian		Y	97121978	44788608
22	Liver	56	Male	Caucasian		Y	91811956	42282929
23	Liver	47	Female	Caucasian		Y	110671808	51553038
24	Liver	70	Male	Caucasian		Y	84448060	44540884

(ii) Human kidney tissues

Number	Tissue	Age	Sex	Race	Tissue Source	Selected 18 samples for subsampling analysis	Number of Raw Reads	Number of Unique Mapped Reads
1	Kidney Cortex	56	Female	Caucasian		Y	62779252	45253435
3	Kidney Cortex	46	Female	Caucasian		Y	58792770	46005702
5	Kidney Cortex	69	Female	Caucasian		N	59355760	22159488
6	Kidney Cortex	55	Male	Caucasian		Y	55527738	38728138
7	Kidney Cortex	55	Male	Caucasian		Y	53197840	26905312
9	Kidney Cortex	52	Male	Caucasian		Y	64374362	26904786

10	Kidney Cortex	90	Female	Caucasian	The samples were normal postmortom or biospy kidney samples (renal cortex) purchased from Asterand (Detroit, USA) or Captial Biosciences (Rockville, USA). Ref: Dahlin, A et al. Gene expression profiling of transporters in the solute carrere and ATP-binding cassette superfamilies in human eye substructures. Mol Pharm. 10(2), 650-63 (2013).	Y	93557570	52642042
11	Kidney Cortex	38	Female	Caucasian		Y	89809126	64068201
13	Kidney Cortex	4	Female	Caucasian		Y	52480948	43258580
17	Kidney Cortex	53	Male	Caucasian		Y	73674810	40695337
18	Kidney Cortex	8	Male	Caucasian		Y	60115224	27734510
19	Kidney Cortex	45	Male	Caucasian		Y	60233332	40856985
21	Kidney Cortex	4	Female	Caucasian		Y	52559678	35445455
24	Kidney Cortex	43	Male	Caucasian		Y	64779326	38887558
25	Kidney Cortex	6	Female	Caucasian		Y	61298068	31392534
28	Kidney Cortex	52	Female	Caucasian		Y	72942822	37827074
29	Kidney Cortex	46	Male	Caucasian		Y	54346380	35303709
30	Kidney Cortex	62	Male	Caucasian		Y	48339104	29641115
31	Kidney Cortex	79	Female	Caucasian		Y	71638562	34751600
32	Kidney Cortex	68	Male	Caucasian		N	45190234	19613545

(iii) Human heart ventricle tissues

Number	Tissue	Age	Sex	Race	Tissue Source	Selected 18 samples for subsampling analysis	Number of Raw Reads	Number of Unique Mapped Reads
1	Heart ventricle	34	M	Caucasian	National Disease Research Interchange (NDRI, Philadelphia, PA, USA) or National Institute of Child Health & Human Development Brain and Tissue Bank (NICHD, Baltimore, MD, USA). All Specimens were collected from Caucasian individuals without cardiac disorder.	Y	89986426	57496576
2	Heart ventricle	27	F	Caucasian		Y	84793502	55413159
3	Heart ventricle	15	F	Caucasian		N	70404508	41813523
4	Heart ventricle	42	F	Caucasian		Y	71134668	35079948
5	Heart ventricle	54	F	Caucasian		Y	86216410	54037908
6	Heart ventricle	18	M	Caucasian		N	171225890	97410738
7	Heart ventricle	60	M	Caucasian		Y	84675428	55369577
8	Heart ventricle	56	M	Caucasian		N	83193276	49752663
9	Heart ventricle	41	M	Caucasian		Y	73803614	45942077
10	Heart ventricle	57	F	Caucasian		Y	96611370	58603837
11	Heart ventricle	20	M	Caucasian		Y	67776840	40658757
12	Heart ventricle	39	F	Caucasian		Y	89552672	48026027
13	Heart ventricle	50	M	Caucasian		Y	67834636	40110806
14	Heart ventricle	48	F	Caucasian		Y	80153734	44163960
15	Heart ventricle	52	M	Caucasian		Y	152250688	78898295

16	Heart ventricle	38	M	Caucasian		Y	69862644	35314685
17	Heart ventricle	21	M	Caucasian		Y	70506838	42647395
18	Heart ventricle	40	F	Caucasian		N	83527236	54878424
19	Heart ventricle	63	F	Caucasian		N	77684194	49045169
20	Heart ventricle	54	M	Caucasian		Y	81806578	49297686
21	Heart ventricle	58	M	Caucasian		Y	82028022	52460405
22	Heart ventricle	41	M	Caucasian		Y	86796980	56908512
23	Heart ventricle	32	F	Caucasian		Y	75019736	50086176
24	Heart ventricle	25	F	Caucasian		N	71450592	49037737
25	Heart ventricle	19	M	Caucasian		N	74759838	49510692

(iv) Human adipose tissues

Number	Tissue	Age	Sex	Race	Tissue Source	Selected 18 samples for subsampling analysis	Number of Raw Reads	Number of Unique Mapped Reads
1	Adipose	55	Female	White	These adipose tissue biopsy specimens were collected from human participants in a dietary intervention clinical trial during the control diet (55% carbohydrate, 30% fat). Participants were ≥ 18 years old, non-smoking, and overweight to obese with BMI between 25 and 40 kg/m2 but otherwise healthy. Ref: Chiu, S et al. Diets high in protein or saturated fat do not affect insulin sensitivity or plamsa concentrations of lipids and lipoproteins in overweight and obese adults. J Nutr. 144(11), 1753-9 (2014).	Y	85736534	59519669
2	Adipose	36	Female	White		Y	83640366	59602281
3	Adipose	47	Male	African America		N	49447548	34783714
4	Adipose	31	Female	African America		N	73916270	54369960
5	Adipose	34	Female	Asian		N	88304362	63241541
6	Adipose	56	Female	White		Y	93083806	64193681
7	Adipose	56	Female	White		Y	82543362	55664950
8	Adipose	23	Female	African America		N	53586144	39623296
9	Adipose	42	Female	unknown		Y	61709462	45082859
10	Adipose	23	Female	unknown		Y	67271848	46964491
11	Adipose	57	Female	White		Y	66104928	43979935
12	Adipose	25	Female	White		Y	49547238	34909751
13	Adipose	45	Female	White		Y	50875584	37640312
14	Adipose	41	Female	White		Y	60135280	40939107
15	Adipose	41	Male	White		Y	65089562	46673558
16	Adipose	35	Male	African America		N	67916144	46874501
17	Adipose	24	Female	Asian		N	50115470	36854289
18	Adipose	28	Female	White		Y	61975066	44723046
19	Adipose	52	Male	White		Y	80692410	56394164
20	Adipose	59	Female	unknown		Y	70007138	46842617
21	Adipose	52	Female	unknown		Y	62487308	42074173
22	Adipose	29	Female	White		Y	102082378	67896823
23	Adipose	43	Male	White		Y	58759486	46246447
24	Adipose	30	Female	unknown		Y	55097874	42835016
25	Adipose	36	Female	Asian		N	54637216	42445588

(v) Lymphoblastoid cell lines

Number	Tissue	Age	Sex	Race	Source	Selected 18 samples for subsampling analysis	Number of Raw Reads	Number of Unique Mapped Reads
1	Cells - EBV transformed lymphocytes	32.78	Male	Caucasian	Immortalized lymphoblastoid cell lines (LCLs) were derived from blood samples isolated from participants of the Cholesterol and Pharmacogenetics (CAP) clinical trial, and grown at 37°C with 5% CO2 in RPMI 1640 media supplemented with 10% FBS, 500 U/ml penicillin/streptomycin, and 2 nmol/L GlutaMAX (Life Technologies). Ref: Simon, JA. Phenotypic predictors of response to simvastatin therapy among African-Americans and Caucasians: the Cholesterol and Pharmacogenetics (CAP) Study. 97(6), 843-50 (2006).	Y	69972926	46443636
2	Cells - EBV transformed lymphocytes	39.79	Male	Caucasian		Y	75594480	53463208
3	Cells - EBV transformed lymphocytes	36.61	Female	Caucasian		N	74795574	52297675
4	Cells - EBV transformed lymphocytes	60.16	Female	Caucasian		N	85471302	48592209
5	Cells - EBV transformed lymphocytes	49.17	Male	Caucasian		Y	92783426	64415559
6	Cells - EBV transformed lymphocytes	75.38	Female	Caucasian		N	58268928	43472851
7	Cells - EBV transformed lymphocytes	68.27	Male	Caucasian		N	55648310	36045052
8	Cells - EBV transformed lymphocytes	55.52	Female	Caucasian		Y	68522488	51089785
9	Cells - EBV transformed lymphocytes	48.39	Female	Caucasian		N	58630148	38834141

10	Cells - EBV transformed lymphocytes	48.11	Male	Caucasian		Y	84444016	56091455
11	Cells - EBV transformed lymphocytes	54.97	Male	Caucasian		N	61357514	43939195
12	Cells - EBV transformed lymphocytes	30.14	Male	Caucasian		Y	101160604	71356057
13	Cells - EBV transformed lymphocytes	57.2	Female	Caucasian		Y	115794752	74586887
14	Cells - EBV transformed lymphocytes	58.95	Female	Caucasian		N	72885284	47346008
15	Cells - EBV transformed lymphocytes	35.15	Male	Caucasian		Y	152314160	96570426
16	Cells - EBV transformed lymphocytes	47.5	Female	Caucasian		N	82053168	53286669
17	Cells - EBV transformed lymphocytes	43.72	Male	Caucasian		N	109555426	72306303
18	Cells - EBV transformed lymphocytes	47.9	Male	Caucasian		N	85930840	56313704
19	Cells - EBV transformed lymphocytes	61.61	Male	Caucasian		N	51594666	39295502

20	Cells - EBV transformed lymphocytes	39.38	Female	Caucasian	Y	76508342	49645812
21	Cells - EBV transformed lymphocytes	55.3	Male	Caucasian	Y	60053104	44091975
22	Cells - EBV transformed lymphocytes	34.79	Female	Caucasian	N	66652646	45631941
23	Cells - EBV transformed lymphocytes	55.34	Male	Caucasian	Y	74609996	53514458
24	Cells - EBV transformed lymphocytes	56.59	Female	Caucasian	N	102911154	75177432
25	Cells - EBV transformed lymphocytes	55.14	Male	Caucasian	N	65205334	49505425
26	Cells - EBV transformed lymphocytes	54.86	Female	Caucasian	N	55295558	40638125
27	Cells - EBV transformed lymphocytes	66.82	Female	Caucasian	Y	129585604	72749855
28	Cells - EBV transformed lymphocytes	62.46	Female	Caucasian	Y	122101926	74173449
29	Cells - EBV transformed lymphocytes	53.09	Female	Caucasian	N	87917948	61659547

30	Cells - EBV transformed lymphocytes	61.57	Male	Caucasian		Y	109350692	73321404
31	Cells - EBV transformed lymphocytes	53.37	Female	Caucasian		N		65485414
32	Cells - EBV transformed lymphocytes	59.04	Male	Caucasian		N		53225501
33	Cells - EBV transformed lymphocytes	37.86	Male	Caucasian		Y	64469612	43366971
34	Cells - EBV transformed lymphocytes	42.07	Male	Caucasian		Y	62572380	45370734
35	Cells - EBV transformed lymphocytes	50.87	Male	Caucasian		N	69861558	48770638
36	Cells - EBV transformed lymphocytes	53.45	Female	Caucasian		N	60815498	45235275
37	Cells - EBV transformed lymphocytes	68.18	Male	Caucasian		N		56501103
38	Cells - EBV transformed lymphocytes	66.82	Female	Caucasian		N	72643362	51300794
39	Cells - EBV transformed lymphocytes	52.41	Male	Caucasian		N	89283478	60251207

40	Cells - EBV transformed lymphocytes	39.83	Female	Caucasian	N	90255134	56119858
41	Cells - EBV transformed lymphocytes	60.42	Male	Caucasian	N	66256126	47099855
42	Cells - EBV transformed lymphocytes	62.29	Female	Caucasian	N	72326344	48102041
43	Cells - EBV transformed lymphocytes	54.01	Male	Caucasian	Y	78219746	56276095
44	Cells - EBV transformed lymphocytes	35.22	Male	Caucasian	Y	86976208	60067824
45	Cells - EBV transformed lymphocytes	44.24	Male	Caucasian	N	84188582	54902701

Supplemental Table S3. Summary of gene expression for the tissues

(a) PHARMACOGENES	LCL	Liver	Kidney	Adipose	Heart	Intersection	Union
Total Number of Genes Mapped and Analyzed	389	387*	389	389	389	389	389
Ubiquitous[1]	116	225	167	190	166	87	291
Total Expressed[2]	188	320	315	274	255	161	364
Total Undetected[3]	17	3	5	1	5	0	22
Number of Specific Genes[3]	3	39	14	3	9	NA	NA
% Ubiquitous (Ubiquitous/Total)	29.80%	58.10%	42.90%	48.80%	42.70%	22.40%	74.81%
% Ubiquitous (Ubiquitous/Total Expressed)	61.70%	70.30%	53.00%	69.30%	65.10%	54.00%	79.95%
% Specific (Specific/Total Expressed)	2.10%	14.00%	5.30%	1.30%	4.00%	NA	NA
(b) ALL PROTEIN CODING	LCL	Liver	Kidney	Adipose	Heart	Intersection	Union
Total Number of Genes Mapped and Analyzed	20025	20012*	20025	20025	20025	20025	20025
Ubiquitous[1]	9685	10375	10838	11274	10574	7845	13509
Total Expressed[2]	12772	14247	15416	14251	11223	11223	16888
Total Undetected[3]	1643	1132	866	725	1242	388	2183
Number of Specific Genes[4]	418	305	494	248	290	NA	NA
% Ubiquitous (Ubiquitous/Total)	48.40%	51.80%	54.10%	56.30%	52.80%	39.20%	67.46%
% Ubiquitous (Ubiquitous/Total Expressed)	75.80%	72.80%	70.30%	79.10%	94.20%	69.90%	79.99%
% Specific (Specific/Total Expressed)	3.20%	2.10%	3.10%	1.70%	2.00%	NA	NA

* Fewer number of genes mapped and analyzed in liver samples. For example in the liver, two pharmacogenes, ALB (albumin) and SERPINA1 (serpin peptidase inhibitor, clade A) were not able to be accurately quantified due to their high expression, and so these genes were excluded from liver analysis.

[1] Genes that have FPKM values ≥ 1 in all 18 individuals of each tissues or LCL.

[2] Total Expressed genes refers to genes that have FPKM ≥ 1 in any 18 individuals of each tissues or LCL.

[3] Total undetected genes refers to genes that have FPKM = 0 in all 18 individuals of each tissues or LCL.

[4] Number of specific genes refers to genes that have FPKM values ≥ 1 in one tissue only and other tissues have FPKM values < 1. List of these genes in Supplementary Table 5.

Supplemental Table S4: Top 10 most variable gene sets (Top 20 for LCLs) (among protein coding genes with median FPKM >1) by tissue type. Enrichment of variability in gene expression in gene sets was calculated using the Gene Set Enrichment Analysis tool (GSEA, Subramanian A. et al. *PNAS* 2005;102(43):15545h 15550.), with coefficient of variation of each gene in a particular tissue type used as gene ranking. Gene sets tested include all gene ontology biological process sets with 15 to 500 genes, and 'PGRN pharmacogenes'. Significance of enrichment was calculated by permuting geneh rank associations 1000 times. Raw enrichment scores, normalized enrichment scores (normalized by mean of enrichment scores over permutations), nominal ph value from permutation, and false discovery rate qh value over all gene sets shown below.

Set Name	Enrichment Score	Normalized Enrichment Score	Nominal Pvalue	Fale Discovery Rate Qvalue
**Heart**				
CELL_SURFACE_RECEPTOR_LINKED_SIGNAL_TRANSDUCTION_GO_0007166	0.24	5.24	0	0
RESPONSE_TO_EXTERNAL_STIMULUS	0.33	5.12	0	0
G_PROTEIN_COUPLED_RECEPTOR_PROTEIN_SIGNALING_PATHWAY	0.36	5.04	0	0
IMMUNE_SYSTEM_PROCESS	0.29	4.93	0	0
IMMUNE_RESPONSE	0.36	4.92	0	0
DEFENSE_RESPONSE	0.36	4.88	0	0
CELL_CELL_SIGNALING	0.28	4.40	0	0
PGRN PHARMACOGENES	0.25	4.19	0	0
RESPONSE_TO_WOUNDING	0.34	4.17	0	0
LOCOMOTORY_BEHAVIOR	0.52	4.04	0	0
**Kidney**				
PGRN PHARMACOGENES	0.35	6.49	0	0
DEFENSE_RESPONSE	0.43	5.87	0	0
RESPONSE_TO_EXTERNAL_STIMULUS	0.35	5.44	0	0
RESPONSE_TO_WOUNDING	0.42	5.24	0	0
IMMUNE_RESPONSE	0.37	4.99	0	0
IMMUNE_SYSTEM_PROCESS	0.30	4.95	0	0
INFLAMMATORY_RESPONSE	0.45	4.72	0	0
CELL_SURFACE_RECEPTOR_LINKED_SIGNAL_TRANSDUCTION_GO_0007166	0.21	4.60	0	0
G_PROTEIN_COUPLED_RECEPTOR_PROTEIN_SIGNALING_PATHWAY	0.31	4.43	0	0
SYSTEM_PROCESS	0.22	4.21	0	0
**Adipose Tissue**				
IMMUNE_SYSTEM_PROCESS	0.30	5.54	0	0
IMMUNE_RESPONSE	0.35	5.37	0	0

DEFENSE_RESPONSE	0.37	5.14	0	0
BEHAVIOR	0.45	4.84	0	0
G_PROTEIN_COUPLED_RECEPTOR_PROTEIN_SIGNALING_PATHWAY	0.32	4.50	0	0
RESPONSE_TO_EXTERNAL_STIMULUS	0.28	4.44	0	0
LOCOMOTORY_BEHAVIOR	0.49	4.38	0	0
PGRN PHARMACOGENES	0.25	4.14	0	0
CELL_SURFACE_RECEPTOR_LINKED_SIGNAL_TRANSDUCTION_GO_0007166	0.19	4.13	0	0
CELLULAR_DEFENSE_RESPONSE	0.61	4.01	0	0
**Liver**				
PGRN PHARMACOGENES	0.27	4.94	0	0
CELL_CELL_SIGNALING	0.28	4.14	0	0
IMMUNE_RESPONSE	0.27	3.91	0	0
CELL_SURFACE_RECEPTOR_LINKED_SIGNAL_TRANSDUCTION_GO_0007166	0.18	3.82	0	0
IMMUNE_SYSTEM_PROCESS	0.22	3.77	0	0
DEFENSE_RESPONSE	0.23	3.35	0	0
DIGESTION	0.64	3.34	0	0
G_PROTEIN_COUPLED_RECEPTOR_PROTEIN_SIGNALING_PATHWAY	0.26	3.33	0	0
SYSTEM_DEVELOPMENT	0.13	3.23	0	0
SYSTEM_PROCESS	0.18	3.17	0	0
**LCLs**				
G_PROTEIN_COUPLED_RECEPTOR_PROTEIN_SIGNALING_PATHWAY	0.45	5.11	0	0
CELL_SURFACE_RECEPTOR_LINKED_SIGNAL_TRANSDUCTION_GO_0007166	0.28	5.06	0	0
RESPONSE_TO_EXTERNAL_STIMULUS	0.29	3.89	0	0
CELL_CELL_SIGNALING	0.31	3.87	0	0
IMMUNE_RESPONSE	0.25	3.52	0	0
MULTICELLULAR_ORGANISMAL_DEVELOPMENT	0.14	3.51	0	0
BEHAVIOR	0.40	3.48	0	0
RESPONSE_TO_WOUNDING	0.33	3.44	0	0
SYSTEM_PROCESS	0.22	3.30	0	0
DEFENSE_RESPONSE	0.26	3.29	0	0
IMMUNE_SYSTEM_PROCESS	0.20	3.23	0	0
ANATOMICAL_STRUCTURE_DEVELOPMENT	0.14	3.20	0	0
LOCOMOTORY_BEHAVIOR	0.42	3.09	0	0
SYSTEM_DEVELOPMENT	0.14	2.98	0	6.49E-05
INFLAMMATORY_RESPONSE	0.33	2.95	0	6.06E-05
PGRN PHARMACOGENES	0.22	2.92	0	1.17E-04

Supplemental Table S5A. List of PGRN pharmacogenes that are expressed at higher levels in a single tissue or LCLs in the dataset. These genes are expressed with FPKM at least 10 times greater than the FPKM in all the other tissues. Tissue-specific gene expression values were calculated by summing FPKM values for all isoforms for a given gene in a given tissue (FPKM values per tissue were calculated by Cuffdiff, one per tissue using 18 samples for that tissue).

LCL	Liver	Kidney	Adipose	Heart
CHST11	ABCB11	ABP1	PPARG	CACNA1C
SPN	ABCB4	CYP24A1		CACNB2
TCL1A	ABCC2	HSD11B2		KCNH2
TNF	ABCG8	SERPINA1		NPPB
TYMS	AC008537.2	SLC13A1		RYR2
	ADH1A	SLC13A2		SCN2B
	ADH1C	SLC22A11		SCN5A
	ADH4	SLC22A12		
	AOX1	SLC22A13		
	APOA1	SLC22A2		
	APOA2	SLC22A6		
	APOA4	SLC22A8		
	APOB	SLC6A3		
	CHST13	SPP1		
	CYP1A2	SULT1C2		
	CYP21A2	SULT2B1		
	CYP26A1	UGT8		
	CYP2A13			
	CYP2A6			
	CYP2A7			
	CYP2B6			
	CYP2C18			
	CYP2C19			
	CYP2C8			
	CYP2C9			
	CYP2D6			
	CYP2E1			
	CYP3A4			
	CYP3A43			
	CYP3A5			
	CYP3A7			
	CYP4F11			
	CYP4F3			

CYP7A1
CYP8B1
DBH
F2
F5
FOXA3
MAT1A
NAT2
NNMT
NR1I2
NR1I3
ORM1
ORM2
PON1
PON3
SERPINA7
SLC10A1
SLC22A1
SLC22A10
SLC22A9
SLCO1B1
SLCO1B3
SULT1B1
SULT1E1
SULT2A1
UGT1A1
UGT1A3
UGT1A4
UGT2B10
UGT2B15
UGT2B4
XDH

Supplemental Table S5B. List of PGRN pharmacogenes that are expressed at lower levels in only one tissue of the dataset. These genes are expressed with FPKM at least 10 times lower in one tissue relative to all other tissues or LCL analyzed in the dataset, and FPKM in other tissues greater than or equal to 1. Tissue-specific gene expression values were calculated by summing FPKM values for all isoforms for a given gene in a given tissue (FPKM values per tissue were calculated by Cuffdiff, one per tissue using 18 samples for that tissue).

LCL	Liver	Kidney	Adipose	Heart
EPHX1			CYP2J2	
COL1A1				
TGFBI				
APOE				
EGFR				
SULT1A1				
SLCO2A1				
CDA				
SLC22A17				
GSTM5				
ATP7B				
AOX1				
SPP1				
ADH1C				
NR3C2				
SLC2A4				
SCN1B				
ADRA1B				
ABCA1				
CYP4F12				
PEAR1				
FCGR3A				
SLC22A3				
SULT1C4				
SLC25A27				
ALDH1A1				
FMO3				
SLC7A8				
CYP39A1				
MAOA				
ORM1				
ADH1B				
AGTR1				
CES1				
PTGIS				
MAOB				
NNMT				

Supplemental Table S6. A total of 20 pairwise comparisons were performed to detect the genes differentially expressed between LCLs and tissues for all protein coding genes (red) and pharmacogenes (blue). The **bold** numbers are the gene expression (FPKM) Spearman correlations between two tissues for tested genes. The numbers inside the parentheses refer to the number of genes differentially expressed between pairs of tissues (U: higher in row tissue/D: higher in column tissue). Genes are considered to be differentially expressed when q<0.10 in both DESeq and Cuffdiff and >2 fold difference in expression. Darker shading indicates higher correlation.

Pharmacogenes

	Liver	Kidney	Adipose	Heart	LCL
**Liver**		**0.40** (U:119/D:69)	**0.43** (U:134/D:48)	**0.31** (U:141/D:58)	**0.27** (U:173/D:38)
**Kidney**	**0.87** (U:3540/D:3023)		**0.64** (U:94/D:51)	**0.61** (U:94/D:48)	**0.51** (U:151/D:39)
**Adipose**	**0.85** (U:3707/D:3862)	**0.87** (U:2940/D:3576)		**0.83** (U:59/D:46)	**0.62** (U:117/D:38)
**Heart**	**0.82** (U:3828/D:3802)	**0.85** (U:2966/D:3335)	**0.9** (U:3118/D:3275)		**0.62** (U:115/D:39)
**LCL**	**0.76** (U:5113/D:3713)	**0.76** (U:5198/D:4265)	**0.77** (U:5848/D:3781)	**0.75** (U:5360/D:3907)	

Protein-coding genes

Supplemental Table S7. Percent of PGRN pharmacogenes that are alternatively spliced by class. Alternatively spliced genes have multiple mutually exclusive junctions each with at least 1 read/100bp and PSI>5 in at least one of the 90 samples. Genes reported as not alternatively spliced were not reported to have any evidence of mutually exclusive junctions in any sample, and have gene FPKM>10 in at least one sample. For other genes, it's unclear because the PSI may be below 5 or the gene may be low expressed and so some alternative splice events may not be observed.

class	#genes	alt. spliced genes	not alt. spliced genes	unclear	% alt. spliced	% not alt. spliced
Other_Transp	4	4	0	0	100.0%	0.0%
ADH_Metabo	7	6	0	1	85.7%	0.0%
Other_Metab	54	45	5	4	83.3%	9.3%
ABC_Transpor	24	20	1	3	83.3%	4.2%
UGT_Metabol	18	15	1	2	83.3%	5.6%
SULT_Metabc	10	8	0	2	80.0%	0.0%
SLC_Transpor	56	43	5	8	76.8%	8.9%
Other	77	55	9	13	71.4%	11.7%
ALDH_Metab	7	5	0	2	71.4%	0.0%
GST_Metabol	17	12	1	4	70.6%	5.9%
Nuclear Rece	24	16	7	1	66.7%	29.2%
CYP_Metaboli	47	30	6	11	63.8%	12.8%
Receptor	29	13	5	11	44.8%	17.2%
Channel	15	6	5	4	40.0%	33.3%
**Total**	**389**	**278**	**45**	**66**	**71.5%**	**11.6%**

Supplemental Table S8. A total of 20 pairwise comparisons were performed to detect the genes differentially alternativel spliced between LCLs and tissues for all protein coding genes (red) and pharmacogenes (blue). Reported are the number of genes (**bold**) with a significantly differentially spliced event (in parentheses is the percent of tested events that were significant). Significant splicing events were determined by a Wilcoxon test on the 'percent spliced in' (PSI) values between two tissues (Benjamini-Hochberg corrected p-value <0.05) and the difference in median PSI between the tissues had to be >5. To be tested, an event must have a coverage of at least 10 reads/100bp in at least half the samples for each of the two tissues. Darker shading indicates a smaller percentage of events are differentially spliced.

Pharmacogenes

	Liver	Kidney	Adipose	Heart	LCL
**Liver**		**18** (6.3%)	**14** (8.2%)	**10** (8.8%)	**14** (15%)
**Kidney**	**590** (9.2%)		**12** (10%)	**8** (6.1%)	**14** (16%)
**Adipose**	**849** (14%)	**826** (12%)		**12** (13%)	**19** (23%)
**Heart**	**787** (15%)	**743** (12%)	**1021** (16%)		**16** (21%)
**LCL**	**1246** (21%)	**1408** (19%)	**1538** (21%)	**1403** (21%)	

Protein-coding genes

Supplemental Table S9: The number of alternative splicing events (A) or genes with alternative splicing events (B) with at least 1 read/100bp and PSI>5 in one tissue and no coverage in any of the other four tissues. The potentially alternatively spliced region must have read coverage of at least 20 reads/100bp in at least 5 samples of each of the other four tissues to control for gene expression differences between tissues.

A Number of splice events observed in only one tissue (# of events in regions with read coverage >20reads/10bbp in at least 5 samples of all four other tissues)

Tissue	Protein-Coding Genes	Pharmacogenes
**Adipose**	20 (of 3718, 0.5%)	2 (of 52, 3.8%)
**Heart**	43 (of 3862, 1.1%)	1 (of 54, 1.9%)
**Liver**	20 (of 4061, 0.5%)	1 (of 53, 1.9%)
**Kidney**	24 (of 3582, 0.7%)	1 (of 48, 2.1%)
**LCLs**	63 (of 3874, 1.6%)	2 (of 75, 2.7%)

B Number of genes with a splice event observed in only one tissue (# of genes with an event in a region with read coverage >20reads/10bbp in at least 5 samples of all four other tissues)

Tissue	Protein-Coding Genes	Pharmacogenes
**Adipose**	22 (of 2152, 1.0%)	1 (of 21, 4.8%)
**Heart**	51 (of 2264, 2.3%)	0 (of 23, 0.0%)
**Liver**	22 (of 2285, 1.0%)	0 (of 21, 0.0%)
**Kidney**	29 (of 2083, 1.4%)	0 (of 19, 0.0%)
**LCLs**	89 (of 2269, 3.9%)	1 (of 31, 3.2%)

Supplemental Table S10. Differences between tissues for QC metrics calculated by RNASeQC by Tukey HSD test. Difference in means between tissues and p-value for test shown below.

	Intragenic.Rate		Exonic.Rate	
**Tissue Comparison**	**Difference**	**Pvalue**	**Difference**	**Pvalue**
**Heart-Adipose**	0.0069	2.40E-03	-0.0279	1.79E-01
**Kidney-Adipose**	-0.0011	-5.65E-03	-0.0179	6.23E-01
**Kidney-Heart**	-0.0080	-1.25E-02	0.0100	9.34E-01
**Liver-Adipose**	0.0078	3.46E-03	0.0022	1.00E+00
**Liver-Heart**	0.0009	-3.42E-03	0.0301	1.06E-01
**Liver-Kidney**	0.0089	4.53E-03	0.0201	4.85E-01
**LCL-Adipose**	0.0262	2.22E-02	0.1577	0.00E+00
**LCL-Heart**	0.0193	1.53E-02	0.1856	0.00E+00
**LCL-Kidney**	0.0273	2.33E-02	0.1756	0.00E+00
**LCL-Liver**	-0.0183	-2.22E-02	-0.1555	0.00E+00

	Intronic.Rate		Intergenic.Rate	
**Tissue Comparison**	**Difference**	**Pvalue**	**Difference**	**Pvalue**
**Heart-Adipose**	0.0348	2.08E-02	-0.0071	2.22E-04
**Kidney-Adipose**	0.0168	5.83E-01	0.0011	9.65E-01
**Kidney-Heart**	-0.0180	5.15E-01	0.0082	1.70E-05
**Liver-Adipose**	0.0056	9.86E-01	-0.0078	2.63E-05
**Liver-Heart**	-0.0291	6.68E-02	-0.0006	9.95E-01
**Liver-Kidney**	-0.0111	8.54E-01	-0.0088	1.58E-06
**LCL-Adipose**	-0.1315	0.00E+00	-0.0264	0.00E+00
**LCL-Heart**	-0.1663	0.00E+00	-0.0193	0.00E+00
**LCL-Kidney**	-0.1483	0.00E+00	-0.0275	0.00E+00
**LCL-Liver**	0.1372	0.00E+00	0.0187	0.00E+00

	Mean.Per.Base.Cov.		Expression.Profiling.Efficiency	
**Tissue Comparison**	**Difference**	**Pvalue**	**Difference**	**Pvalue**
**Heart-Adipose**	0.1020	1.00E+00	-0.0279	1.79E-01
**Kidney-Adipose**	-11.5318	4.68E-03	-0.0179	6.23E-01
**Kidney-Heart**	-11.6338	4.21E-03	0.0100	9.34E-01
**Liver-Adipose**	-15.1393	3.26E-05	0.0022	1.00E+00
**Liver-Heart**	-15.2413	2.83E-05	0.0301	1.06E-01
**Liver-Kidney**	-3.6074	7.84E-01	0.0201	4.85E-01
**LCL-Adipose**	31.4712	0.00E+00	0.1577	0.00E+00
**LCL-Heart**	31.3692	0.00E+00	0.1856	0.00E+00
**LCL-Kidney**	43.0030	0.00E+00	0.1756	0.00E+00
**LCL-Liver**	-46.6105	0.00E+00	-0.1555	0.00E+00

	Fragment.Length.StdDev		Fragment.Length.Mean	
Tissue Comparison	Difference	Pvalue	Difference	Pvalue
Heart-Adipose	4.4400	6.13E-01	2.9600	9.29E-01
Kidney-Adipose	0.5750	1.00E+00	-5.6683	5.47E-01
Kidney-Heart	-3.8650	7.35E-01	-8.6283	1.44E-01
Liver-Adipose	2.3786	9.35E-01	-5.5671	5.27E-01
Liver-Heart	-2.0614	9.60E-01	-8.5271	1.25E-01
Liver-Kidney	1.8036	9.77E-01	0.1012	1.00E+00
LCL-Adipose	79.3111	0.00E+00	51.6622	0.00E+00
LCL-Heart	74.8711	0.00E+00	48.7022	0.00E+00
LCL-Kidney	78.7361	0.00E+00	57.3306	0.00E+00
LCL-Liver	-76.9325	0.00E+00	-57.2294	0.00E+00

	rRNA.rate		5prime100baseNorm	
Tissue Comparison	Difference	Pvalue	Difference	Pvalue
Heart-Adipose	0.0024	2.22E-03	0.0010	1.00E+00
Kidney-Adipose	0.0036	9.57E-07	-0.0327	8.99E-03
Kidney-Heart	0.0012	3.25E-01	-0.0337	6.44E-03
Liver-Adipose	0.0012	3.36E-01	-0.0510	2.44E-06
Liver-Heart	-0.0012	2.72E-01	-0.0520	1.48E-06
Liver-Kidney	-0.0025	1.26E-03	-0.0183	3.11E-01
LCL-Adipose	-0.0030	4.23E-06	0.0535	4.06E-08
LCL-Heart	-0.0054	0.00E+00	0.0525	7.32E-08
LCL-Kidney	-0.0066	0.00E+00	0.0862	0.00E+00
LCL-Liver	0.0042	2.99E-11	-0.1045	0.00E+00

	End.1.Mismatch.Rate		End.2.Mismatch.Rate	
Tissue Comparison	Difference	Pvalue	Difference	Pvalue
Heart-Adipose	-0.0005	8.54E-02	-0.0008	6.51E-03
Kidney-Adipose	-0.0004	2.37E-01	-0.0009	3.35E-04
Kidney-Heart	0.0001	9.91E-01	-0.0002	9.12E-01
Liver-Adipose	-0.0004	3.17E-01	-0.0012	7.75E-07
Liver-Heart	0.0001	9.53E-01	-0.0005	2.17E-01
Liver-Kidney	0.0000	9.99E-01	-0.0003	7.42E-01
LCL-Adipose	0.0003	4.87E-01	0.0004	2.66E-01
LCL-Heart	0.0008	1.29E-04	0.0011	2.26E-07
LCL-Kidney	0.0007	1.17E-03	0.0013	2.47E-09
LCL-Liver	-0.0007	1.58E-03	-0.0016	1.25E-13

	Base.Mismatch.Rate		End.1.Mapping.Rate	
Tissue Comparison	Difference	Pvalue	Difference	Pvalue
Heart-Adipose	-0.0006	1.45E-02	-0.0008	9.48E-01
Kidney-Adipose	-0.0007	7.36E-03	0.0007	9.70E-01
Kidney-Heart	0.0000	9.99E-01	0.0014	6.56E-01
Liver-Adipose	-0.0008	6.42E-04	-0.0027	6.84E-02

**Liver-Heart**	-0.0002	9.25E-01	-0.0019	3.29E-01
**Liver-Kidney**	-0.0001	9.81E-01	-0.0033	1.19E-02
**LCL-Adipose**	0.0003	3.06E-01	0.0035	1.83E-03
**LCL-Heart**	0.0010	1.30E-06	0.0043	7.22E-05
**LCL-Kidney**	0.0010	4.89E-07	0.0028	2.21E-02
**LCL-Liver**	-0.0011	4.70E-09	-0.0062	1.04E-09

	**End.2.Mapping.Rate**	
**Tissue Comparison**	**Difference**	**Pvalue**
**Heart-Adipose**	0.0008	9.48E-01
**Kidney-Adipose**	-0.0007	9.70E-01
**Kidney-Heart**	-0.0014	6.56E-01
**Liver-Adipose**	0.0027	6.84E-02
**Liver-Heart**	0.0019	3.29E-01
**Liver-Kidney**	0.0033	1.19E-02
**LCL-Adipose**	-0.0035	1.83E-03
**LCL-Heart**	-0.0043	7.22E-05
**LCL-Kidney**	-0.0028	2.21E-02
**LCL-Liver**	0.0062	1.04E-09

Supplemental Table S11. Housekeeping genes show low variability in FPKM values. (A) Between individuals: values shown are standard deviation of log2(FPKM) values across individuals for each tissue. (B) Between tissues: values shown are the ratio of tissue-specific gene expression/geometric mean of expression across tissues. Housekeeping genes and definitions of variability from Eisenberg E, Levanon EY. Human housekeeping genes, revisited. Trends in genetics : TIG. 2013;29(10):569-74.

A	C15orf24	C1orf43	CHMP2A	GPI	PSMB2	PSMB4	RAB7A	REEP5	SNRPD3	VCP	VPS29
Kidney	0.36	0.38	0.51	0.45	0.42	0.38	0.33	0.38	0.32	0.47	0.35
Heart	0.32	0.40	0.42	0.29	0.22	0.28	0.37	0.28	0.49	0.49	0.27
Adipose	0.22	0.19	0.22	0.22	0.14	0.19	0.17	0.14	0.30	0.23	0.15
Liver	0.23	0.35	0.35	0.45	0.27	0.34	0.32	0.21	0.28	0.49	0.19
LCL	0.15	0.15	0.28	0.32	0.17	0.28	0.25	0.34	0.26	0.29	0.16

B	C15orf24	C1orf43	CHMP2A	GPI	PSMB2	PSMB4	RAB7A	REEP5	SNRPD3	VCP	VPS29
Kidney	0.53	0.56	1.03	0.35	0.59	0.88	0.90	0.66	0.57	0.68	0.26
Heart	0.79	1.07	0.85	1.17	0.58	0.66	0.97	1.24	0.79	1.14	0.82
Adipose	0.95	0.82	0.91	0.75	0.72	0.85	0.98	0.96	0.51	0.77	1.08
Liver	1.38	1.34	1.08	0.83	1.46	1.11	0.86	1.10	1.21	1.02	0.94
LCL	0.88	0.92	1.09	2.09	2.72	2.13	1.47	0.52	2.66	1.90	1.44

Supplemental Table S12. Reported are the number of pharmacogenes with a significantly differentially spliced event between tissues. Significant splicing events were determined by a Wilcoxon test on the 'percent spliced in' (PSI) values between two tissues (Benjamini-Hochberg corrected p-value <0.05) and the difference in median PSI between the tissues had to be >5. To be tested, an event must have a coverage of at least 10 reads/100bp in at least half the samples for each of the two tissues.

Tissue 1	Tissue 2	Significantly differentially spliced in PGRN data	Also with sufficient coverage to be tested in GTEx data	Also significantly differentially spliced in GTEx data	% of tested in GTEx, sig in GTEx
Liver	Heart	10	6	4	67%
Liver	Kidney*	18	14	9	64%
Heart	Adipose	12	8	4	50%
Liver	Adipose	14	9	4	44%
Kidney*	Heart	8	5	2	40%
Adipose	LCLs	19	8	2	25%
Liver	LCLs	14	5	1	20%
Kidney*	LCLs	14	5	1	20%
Heart	LCLs	16	7	1	14%
Kidney*	Adipose	12	8	1	13%
		**137**	**75**	**29**	**39%**

*GTEx has only 8 kidney samples, so the power to find significant differential splicing is reduced

Supplemental Table S13. Splice events in PGRN pharmacogenes with PSI (percent spliced in) ≥5 and coverage ≥1 read/100bp in at least one sample of one tissue and no coverage in any of the four other tissues. The ones checked in the GTEx data and observed to also only be present in one tissue had ≥1 read/100bp in at least one sample of one tissue and no coverage in any of the four other tissues. For a number of events, matching the events between the PGRN and GTEx runs of JuncBASE was non-trivial, possibly due to lack of read coverage or increased splicing complexity in the GTEx data.

Tissue	Splice events observed in only one tissue in PGRN data	Checked in GTEx data	Also only observed in one tissue in GTEx	% of checked in GTEx, specific in GTEx
Liver	179	75	69	92%
Kidney	80	36	32	89%
Heart	96	25	16	64%
Adipose	38	5	5	100%
LCLs	63	16	12	75%
	**456**	**157**	**134**	**85%**
